# Molecular processes during fat cell development revealed by gene expression profiling and functional annotation

**DOI:** 10.1186/gb-2005-6-13-r108

**Published:** 2005-12-19

**Authors:** Hubert Hackl, Thomas Rainer Burkard, Alexander Sturn, Renee Rubio, Alexander Schleiffer, Sun Tian, John Quackenbush, Frank Eisenhaber, Zlatko Trajanoski

**Affiliations:** 1 Institute for Genomics and Bioinformatics and Christian Doppler Laboratory for Genomics and Bioinformatics, Graz University of Technology, Petersgasse 14, 8010 Graz, Austria; 2 Research Institute of Molecular Pathology, Dr Bohr-Gasse 7, 1030 Vienna, Austria; 3 Dana-Farber Cancer Institute, Department of Biostatistics and Computational Biology, 44 Binney Street, Boston, MA 02115

## Abstract

**Background:**

Large-scale transcription profiling of cell models and model organisms can identify novel molecular components involved in fat cell development. Detailed characterization of the sequences of identified gene products has not been done and global mechanisms have not been investigated. We evaluated the extent to which molecular processes can be revealed by expression profiling and functional annotation of genes that are differentially expressed during fat cell development.

**Results:**

Mouse microarrays with more than 27,000 elements were developed, and transcriptional profiles of 3T3-L1 cells (pre-adipocyte cells) were monitored during differentiation. In total, 780 differentially expressed expressed sequence tags (ESTs) were subjected to in-depth bioinformatics analyses. The analysis of 3'-untranslated region sequences from 395 ESTs showed that 71% of the differentially expressed genes could be regulated by microRNAs. A molecular atlas of fat cell development was then constructed by *de novo *functional annotation on a sequence segment/domain-wise basis of 659 protein sequences, and subsequent mapping onto known pathways, possible cellular roles, and subcellular localizations. Key enzymes in 27 out of 36 investigated metabolic pathways were regulated at the transcriptional level, typically at the rate-limiting steps in these pathways. Also, coexpressed genes rarely shared consensus transcription-factor binding sites, and were typically not clustered in adjacent chromosomal regions, but were instead widely dispersed throughout the genome.

**Conclusions:**

Large-scale transcription profiling in conjunction with sophisticated bioinformatics analyses can provide not only a list of novel players in a particular setting but also a global view on biological processes and molecular networks.

## Abstract 2

In-depth bioinformatics analyses of expressed sequence tags found to be differentially expressed during differentiation of 3T3-L1 pre-adipocyte cells were combined with de novo functional annotation and mapping onto known pathways to generate a molecular atlas of fat-cell development.

## Background

Obesity, the excess deposition of adipose tissue, is among the most pressing health problems both in the Western world and in developing countries. Growth of adipose tissue is the result of the development of new fat cells from precursor cells. This process of fat cell development, known as adipogenesis, leads to the accumulation of lipids and an increase in the number and size of fat cells. Adipogenesis has been extensively studied *in vitro *for more than 30 years using the 3T3-L1 preadipocyte cell line as a model. This cell line was derived from disaggregated mouse embryos and selected based on the propensity of these cells to differentiate into adipocytes in culture [1]. When exposed to the appropriate adipogenic cocktail containing dexamethasone, isobutylmethylxanthine, insulin, and fetal bovine serum, 3T3-L1 preadipocytes differentiate into adipocytes [2].

Experimental studies on adipogenesis have revealed many important molecular mechanisms. For example, two of the CCAAT/enhancer binding proteins (C/EBPs; specifically C/EBPβ and C/EBPδ) are induced in the early phase of differentiation. These factors mediate transcriptional activity of C/EBPα and peroxisome proliferator-activated receptor (PPAR)-gamma (PPARγ) [3,4]. Another factor, the basic helix-loop-helix (bHLH) transcription factor adipocyte determination and differentiation dependent factor 1/sterol regulatory element binding protein 1 (ADD1/SREBP1c), could potentially be involved in a mechanism that links lipogenesis and adipogenesis. ADD1/SREBP1c can activate a broad program of genes that are involved in fatty acid and triglyceride metabolism in both fat and liver, and can also accelerate adipogenesis [5]. Activation of the adipogenesis process by ADD1/SREBP1c could be effected via direct activation of PPARγ [6] or through generation of endogenous ligands for PPARγ [7].

Knowledge of the transcriptional network is far from complete. In order to identify new components involved in fat cell development, several studies using microarrays have been initiated. These studies have used early Affymetrix technology [8-14] or filters [15], and might have missed many genes that are important to the development of a fat cell. The problem of achieving broad coverage of the developmental transcriptome became evident in a mouse embryo expressed sequence tag (EST) project, which revealed that a significant fraction of the genes are not represented in the collections of genes previously available [16]. Moreover, earlier studies on adipogenesis [8-14] focused on gene discovery for further functional analyses and did not address global mechanisms.

We conducted the present study to evaluate the extent to which molecular processes underlying fat cell development can be revealed by expression profiling. To this end, we used a recently developed cDNA microarray with 27,648 ESTs [17], of which 15,000 are developmental ESTs representing 78% novel and 22% known genes [18]. We then assayed expression profiles from 3T3-L1 cells during differentiation using biological and technical replicates. Finally, we performed comprehensive bioinformatics analyses, including *de novo *functional annotation and curation of the generated data within the context of biological pathways. Using these methods we were able to develop a molecular atlas of fat cell development. We demonstrate the power of the atlas by highlighting selected genes and molecular processes. With this comprehensive approach, we show that key loci of transcriptional regulation are often enzymes that control the rate-limiting steps of metabolic pathways, and that coexpressed genes often do not share consensus promoter sequences or adjacent locations on the chromosome.

## Results

### Expression profiles during adipocyte differentiation

The 3T3-L1 cell line treated with a differentiation cocktail was used as a model to study gene expression profiles during adipogenesis. Three independent time series differentiation experiments were performed. RNA was isolated at the preconfluent stage (reference) and at eight time points after confluence (0, 6, 12, 24, 48 and 72 hours, and 7 and 14 days). Gene expression levels relative to the preconfluent state were determined using custom-designed microarrays with spotted polymerase chain reaction (PCR) products. The microarray developed here contains 27,648 spots with mouse cDNA clones representing 16,016 different genes (UniGene clusters). These include 15,000 developmental clones (the NIA cDNA clone set from the US National Institute of Aging of the National Institutes of Health NIH), 11,000 clones from different brain regions in the mouse (Brain Molecular Anatomy Project [BMAP]), and 627 clones for genes which were selected using the TIGR Mouse Gene Index, Build 5.0 [19].

All hybridizations were repeated with reversed dye assignment. The data were filtered, normalized, and averaged over biological replicates. Data processing and normalization are described in detail under Materials and methods (see below). Signals at all time points could be detected from 14,368 elements. From these microarray data, we identified 5205 ESTs that exhibited significant differential expression between time points and had a complete profile (*P *< 0.05, one-way analysis of variance [ANOVA]). Because ANOVA filters out ESTs with flat expression profiles, we used a fold change criteria to select the ESTs for further analysis. We focused on 780 ESTs that had a complete profile over all time points, and that were more than twofold upregulated or downregulated in at least four of those time points. These stringent criteria were necessary to select a subset of the ESTs for in-depth sequence analysis and for examination of the dynamics of the molecular processes. The overlap between the ANOVA and twofold filtered ESTs was 414. All of the data, together with annotations and other files used in the analyses, are available as Additional data files and on our website [20]. The analyses described in the following text were conducted in the set of 780 ESTs.

### Validation of expression data

Four lines of evidence support the quality of our data and its consistency with existing knowledge of fat cell biology. First, our array data are consistent with reverse transcriptase (RT)-PCR analysis. We compared the microarray data with quantitative RT-PCR for six different genes (*Pparg *[number 592, cluster 6], *Lpl *[number 14, cluster 6], *Myc *[number 224, cluster 11], *Dcn *[number 137, cluster 7], *Ccna2 *[number 26, cluster 5/8], and *Klf9 *[number 6, cluster 9]) at different time points (Additional data file 9 and on our website [20]). A high degree of correlation was found (r^2 ^= 0.87), confirming the validity of the microarray data.

Second, statistical analyses of the independent experiments showed that the reproducibility of the generated data is very high. The Pearson correlation coefficient between the replicates was between 0.73 and 0.97 at different time points. The mean coefficient of variation across all genes at each time point was between 0.11 and 0.27. The row data and the details of the statistical analyses can be found in Additional data file 10 and on our website [20].

Third, comparison between our data and the Gene Atlas V2 mouse data for adipose tissue [21] shows that the consistency of the two data sets increases with differentiation state (Additional data files 11 and 12, and our on website [20]). Therefore, this analysis supports the relevance of the chosen cell model to *in vivo *adipogenesis. Among the 382 transcriptionally modulated genes common in both data sets, 67% are regulated in the same direction at time point zero (confluent pre-adipocyte cell culture). At the final stage of differentiation, the correlation increases up to 72%. If the Gene Atlas expression data are restricted to strongly regulated genes (at least twofold and fourfold change respectively), then the consistency in mature adipocytes rises to 82% (135 genes) and 93% (42 genes), respectively. Out of all 60 tissues in the Gene Atlas V2 mouse, the adipose tissue describes the differentiated state of the 3T3-L1 cells best. Brown fat tissue is the second best match to the differentiated adipocytes (69% of the 382 genes), followed by adrenal gland (66%), kidney (65%), and heart (64%). At each time point in which cell cycle genes were not repressed (12 hours and 24 hours), all tissues had similar correlation to the data set (44-55% for 382 genes).

The fourth line of evidence supporting the quality of our data is that there is clear correspondence between our data and a previously published data set [8]. For a group of 153 genes shared among the two studies, the same upregulation or downregulation was found for 72-89% (depending on time point) of all genes (see Additional data file 13 and our website [20]). The highest identity (89%) was found for the stage terminally differentiated 3T3-L1 cells, for which the profile is less dependent on the precise extraction time. If the comparison is restricted to expression values that are strongly regulated in both experiments (at least twofold change at day 14, 96 ESTs), then the coincidence at every time point is greater than 90%. Comparisons with this [8] and two additional data sets [9,12], and the data pre-processing steps are given in Additional data files 13, 14, 15 and on our website [20]. Note that, because of the differences in the used microarray platforms, availability of the data, normalization methods, and annotations, only 96 genes are shared between all four studies. Of the 780 ESTs monitored in our work, 326 were not detected in the previous studies [8,9,12]: 106 RefSeqs (with prefix NM), 43 automatically generated RefSeqs (with prefix XM), and 173 ESTs (Additional data file 16). 

### Correspondence between transcriptional coexpression and gene function

**Figure 1:**
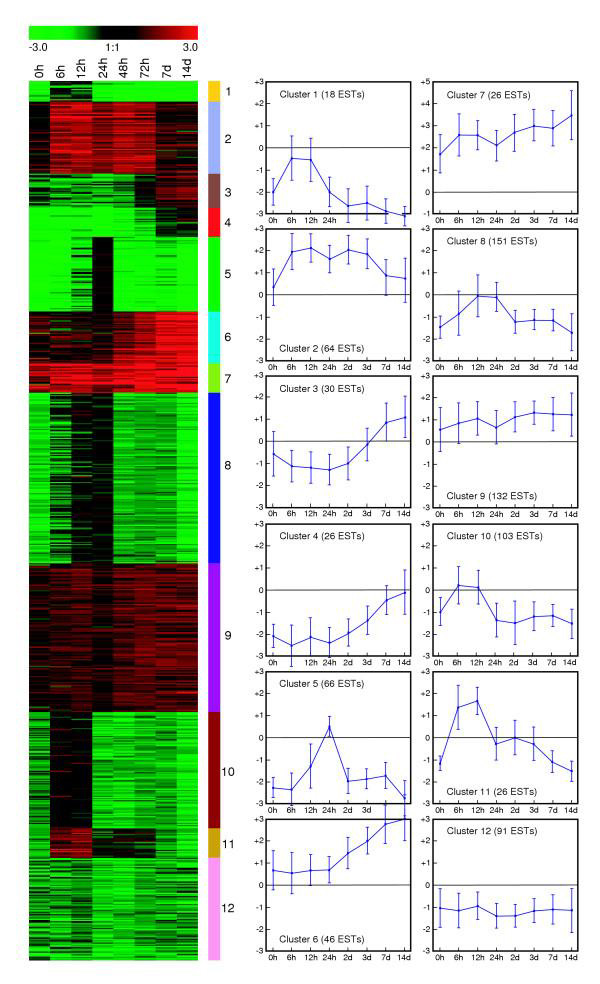
Clustering of ESTs found to be differentially expressed during fat cell differentiation. Shown is k-means clustering of 780 ESTs found to be more than twofold upregulated or downregulated at a minimum of four time points during fat cell differentiation. ESTs were grouped into 12 clusters with distinct expression profiles. Relative expression levels (log_2 _ratios) for EST gene at different time points are shown and color coded according to the legend at the top (left) and expression profile (mean ± standard deviation) for each cluster (right). EST, expressed sequence tag.

To examine the relationships between coexpression and gene functions, we first clustered 780 ESTs that were twofold differentially expressed into 12 temporally distinct patterns, containing between 23 and 143 ESTs (Figure 1). ESTs in four of the clusters are mostly upregulated during adipogenesis, whereas genes in the other eight clusters are mostly downregulated.

**Figure 2:**
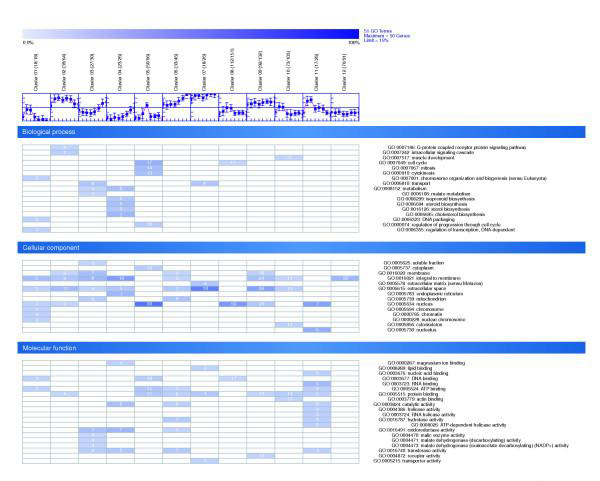
Distribution of GO terms for genes/ESTs in each cluster. The GO terms listed here are those present in at least 15% of the genes within the cluster. In brackets are the number of genes/ESTs with associated GO terms and the number of genes/ESTs within the cluster. EST, expressed sequence tag; GO, Gene Ontology.

We then categorized ESTs with available RefSeq annotation and Gene Ontology (GO) term (486 out of 780) for molecular function, cellular component, and biological process (Figure 2). Genes in clusters 5 and 8 are downregulated through the whole differentiation process and upregulated at 12/24 hours. Many of the proteins encoded by these genes are involved in cell cycle processes and were residing in the nucleus (Figure 2). Re-entry into the cell cycle of growth arrested pre-adipocytes is known as the clonal expansion phase and considered to be a prerequisite for terminal differentiation in 3T3-L1 adipocytes [22]. Genes grouped in cluster 2 are highly expressed from 6 hours (onset of clonal expansion) to 3 days (start of the appearance of adipocyte morphology) but are only modestly expressed at the terminal adipocyte differentiation stage. These include a number of genes that encode signaling molecules. Genes increasingly expressed toward the terminal differentiation stage are in clusters 4, 6, and 7, although from different starting values. Some genes in cluster 6 are known players in lipid metabolism and mitochondrial fatty acid metabolism, whereas some genes can be associated with cholesterol biosynthesis and related to extracellular space or matrix in clusters 4 and 7, respectively.

### Correspondence between coexpression and targeting by microRNAs

Previous studies suggest that protein production for 10% or more of all human and mouse genes are regulated by microRNAs (miRNAs) [23,24]. miRNAs are short, noncoding, single-strand RNA species that are found in a wide variety of organisms. miRNAs cause the translational repression or cleavage of target messages [25]. Some miRNAs may behave like small interfering RNAs. It appears that the extent of base pairing between the small RNA and the mRNA determines the balance between cleavage and degradation [26]. Rules for matches between miRNA and target messages have been deduced from a range of experiments [24] and applied to the prediction and discovery of mammalian miRNA targets [23,27]. Moreover, it was shown that human miRNA-143 is involved in adipocyte differentiation [28].

**Figure 3:**
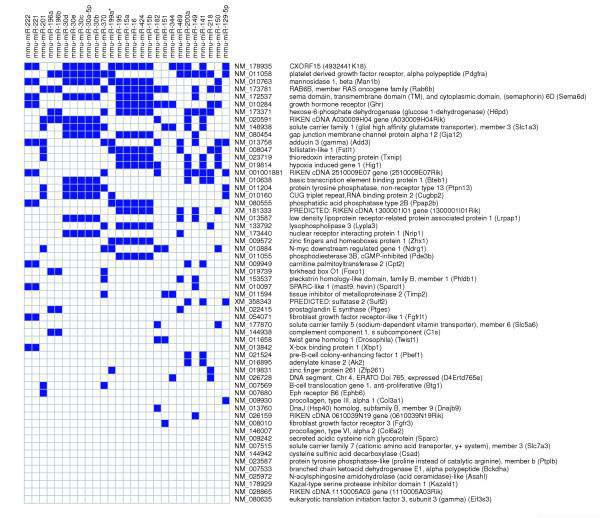
Genes in cluster 9 and significantly over-represented miRNA motifs (blue squares). miRNA, microRNA.

Here we conducted an analysis to determine which of the 780 ESTs differentially expressed during adipocyte differentiation were potential targets of miRNAs and whether there is an over-representation of miRNA targets of coexpressed ESTs clustered in 12 distinct expression patterns. From the 780 ESTs, the 3'-untranslated region (UTR) could be derived for 539. Of these, 518 had at least one exact antisense match for the seven-nucleotide miRNA seed (base 2-8 at the 5' end) from the 234 miRNA sequences (18-24 base pairs [bp]; Additional data file 14). From 395 ESTs with a unique 3'-UTR, 282 (71%) had at least one match over-represented compared with the whole 3'-UTR sequence set (21,396; *P *< 0.05, by one-sided Fisher's exact test). The distribution of statistically over-represented miRNA motifs in 3'-UTRs across the clusters was variable, with genes grouped in cluster 9 (including many transcriptional regulators) having the most statistically over-represented miRNA motifs and genes in cluster 5 having no detectable motifs (Additional data file 18). The results of the analysis of cluster 9 are given in Figure 3. One of the genes with the most significantly over-represented miRNA motifs in the 3'-UTR is related to the ras family (Figure 3). It was previously shown that human oncogene RAS is regulated by let-7 miRNA [29]. Further potential miRNA target genes from all clusters are given in Additional data files 9, 18, 19, 20, 21, 22, 23, 24, 25, 26, 27, 28, 29, 30.

### Molecular atlas of fat cell development derived by *de novo* functional annotation of differentially expressed ESTs

**Figure 4:**
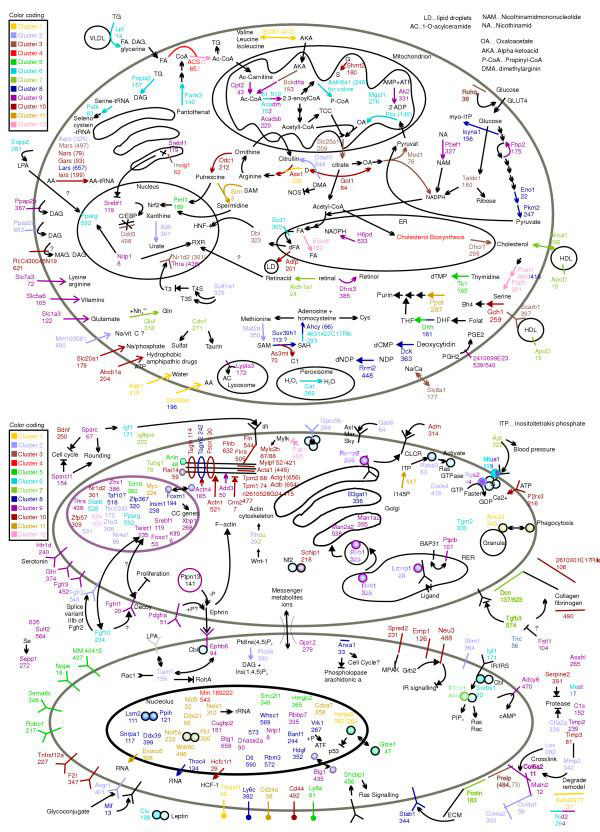
Cellular localization of gene products. Shown are the cellular localizations of gene products involved in (**a**) metabolism and (**b**) other biological processes during fat cell differentiation. Gene products are color coded for each of the 12 clusters (key given to the left of the figure). The numbering is given according to the *de novo *functional annotation (Additional data files 6, 7, 8).

In order to functionally characterize the molecular components underlying adipogenesis in detail, comprehensive bioinformatics analyses of 780 differentially expressed ESTs were performed. A total of 659 protein sequences could be derived, and these were subjected to in-depth sequence analytic procedures. The protein sequences have been annotated *de novo *using 40 academic prediction tools integrated in the ANNOTATOR sequence analysis system. The structure and function was annotated on a sequence segment/domain-wise basis. After extensive literature search and curation using the sequence architecture, 345 gene products were mapped onto known pathways, possible cellular roles, and subcellular localizations (Figure 4) using the PathwayExplorer web service [30] as well as manual literature and domain-based assignment. The results of the sequence analyses and additional information is available in the supplementary material available on our website [20] and Additional data files 6, 7, 8.

This molecular atlas of fat cell development provides the first global view of the underlying biomolecular networks and represents a unique resource for deriving testable hypotheses for future studies on individual genes. Below we demonstrate the usefulness of the atlas by highlighting the following: established regulators of fat cell development, recently discovered fat cell gene products, and candidate transcription factors expressed during adipogenesis. The numbering of the genes is given according to the *de novo *functional annotation (Additional data file 7).

#### Established regulators of fat cell development

Key transcription factors SREBF1 (*Srebf1 *[number 119, cluster 9]) and PPARγ (*Pparg *[number 592, cluster 6]) were highly expressed during the late phase of differentiation. PPARγ [31] (*Pparg *[number 592, cluster 6]) is increasing up to about 15-fold. *Srebf1 *processing is inhibited by insulin-induced gene 1 (*Insig1 *[number 62, cluster 3/4]) through binding of the SREBP cleavage-activation protein [32,33]. *Insig1 *is regulated by *Srebf1 *and *Pparg *at the transcriptional level [34] and the expression of known marker genes of the differentiated adipocyte was increased in parallel with these factors. These include genes from clusters 3, 6, and 9 that are targets of either of these factors: lipoprotein lipase (*Lpl *[number 14, cluster 6]), c-Cbl-associated protein (*Sorbs1 *[number 92, cluster 6]), stearoyl-CoA desaturase 1 (*Scd1 *[number 305, cluster 6]), carnitine palmitoyltransferase II (*Cpt2 *[number 43, cluster 9]), and acyl-CoA dehydrogenase (*Acadm *[number 153, clusters 6 and 9]).

#### Recently discovered fat cell gene products

During the preparation of the manuscript, a number of factors shown to be important to adipocyte function were identified *in vivo*. All of these factors, which have a possible role in the pathogenesis of obesity and insulin resistance, were highly expressed in the present study. Adipose triglyceride lipase (*Pnpla2 *[number 157, cluster 6]), a patatin domain-containing triglyceride lipase that catalyzes the initial step in triglyceride hydrolysis [35], was more than 20-fold upregulated at the terminal differentiation phase. Another example is Visfatin, which is identical to the pre-B cell colony-enhancing factor (*Pbef *[number 327, cluster 9]). This 52 kDa cytokine has enzymatic function in adipocytes, exerts insulin-mimetic effects in cultured cells, and lowers plasma glucose levels in mice by binding to the insulin receptor [36-38]. The imprinted gene mesoderm-specific transcript (*Mest *[number 17, cluster 6/9]), which appears to enlarge adipocytes and could be a novel marker of the size of adipocytes [12], is upregulated during the late stage of 3T3-L1 differentiation.

Members of the Krüppel-like factor (Klf) family, also known as basic transcription element binding proteins, are relevant within the context of adipocyte differentiation. Klf2 was shown to inhibit PPARγ expression and to be a negative regulator of adipocyte differentiation [39]; Klf5 [40], Klf6 [41], and Klf15 [42] have been demonstrated to induce adipocyte differentiation. Whereas Klf9 (*Bteb1 *[number 6, cluster 9]) was upregulated in the intermediate phase in the present study, Klf4 (number 100, cluster 12), which was shown to exert effects on cell proliferation opposing those of Klf5 [43], was downregulated. Another twofold upregulated player is Forkhead box O1 (*Foxo1 *[number 53, cluster 9]), which mediates effects of insulin on the cell. Activation occurs before the onset of terminal differentiation, when Foxo1 becomes dephosphorylated and localizes to the nucleus [44,45]. The glucocorticoid-induced leucine zipper (*Tsc22d3*/*Gilz *[number 173, cluster 2]) functions as a transcriptional repressor of PPARγ and can antagonize glucocorticoid-induced adipogenesis [46,47]. This is consistent with our observation that Gilz is highly upregulated during the first two days, when dexamethasone is present in the medium, and downregulated at the end of differentiation, when PPARγ is highly induced. C/EBP homologous protein 10 (*Ddit3 *[number 498, cluster 3]), another type of transcriptional repressor that forms nonfunctional heterodimers with members of the C/EBP family, was early induced and then downregulated. This might be sufficient to restore the transcriptional activity of C/EBPβ and C/EBPδ [42]. The transcription factor insulinoma-associated 1 (*Insm1 *[number 238, cluster 8]) is associated with differentiation into insulin-positive cells and is expressed during embryo development, where it can bind the PPARγ target Cbl-associated protein (*Sorbs1 *[number 92, cluster 6]; upregulated after induction) [48,49].

#### Candidate transcription factors expressed during adipogenesis

Because knowledge of the transcriptional network during adipogenesis is far from complete, expression profiles have been generated and screened for candidate transcription factors [8,9,12]. Here, we identified a number of transcription factors the exhibit distinct kinetic profiles during adipocyte differentiation that were previously not functionally associated with adipogenesis. Two transcription factors were unique to the present study (Zhx3 and Zfp367), and three more were confirmed (Zhx1, Twist1 and Tcf19) and annotated in the pathway context.

We found evidence for a role of the zinc finger and homeobox protein 3 (*Zhx3 *[number 306, cluster 2]). Zhx3 as well as Zinc finger and homeobox protein 1 (*Zhx1 *[number 386, cluster 9]) might attach to nuclear factor Y, which in turn binds many CCAAT and Y-box elements [50]. We also provide data regarding the expression of zinc finger protein 367 (*Zfp367 *[number 320, cluster 8]) during adipogenesis. The molecular function of Zfp367 is as yet uncharacterized.

Additionally, we provide further experimental evidence and pathway context for candidate transcription factors previously identified in microarray screens [9,12], namely Twist1 and Tcf19. The Twist gene homolog 1 (*Twist1 *[number 235, cluster 9]) was about two- to threefold upregulated at 0 hours, 72 hours, 7 days, and 14 days. Twist1 is a reversible inhibitor of muscle differentiation [51]. Heterozygous double mutants (*Twist1*^-/+^, *Twist2*^-/+^) exhibit loss of subcutaneous adipose tissue and severe fat deficiency in internal organs [52]. Twist1 is a downstream target of nuclear factor-κB and can repress transcription of tumor necrosis factor-α, which is a potent repressor of adipogenesis [52,53]. The differential expression during adipogenesis of Tcf19 was also confirmed in the present study. Tcf19 is a transcription regulator that is involved in cell cycle processes at later stages in cell cycle progression [54]. Expression of other regulators that are involved in the same process support this observation. Forkhead box M1 (*Foxm1 *[number 194, cluster 8]) stimulates the expression of cell cycle genes (for instance the genes encoding cyclin B1 and cyclin B2, and *Cdc25B *and *Cdk1*). In addition, TAF10 RNA polymerase II, also known as TATA box binding protein-associated factor (*Taf10 *[number 518, cluster 8]), is involved in G_1_/S progression and cyclin E expression [55].

### Correspondence between phenotypic changes and gene expression

In addition to the metabolic networks, the molecular atlas also provides a bird's eye view of other molecular processes, including signaling, the cell cycle, remodeling of the extracellular matrix, and cytoskeletal changes. Changes that occur during adipogenesis (phenotypically seen as rounding of densely packed cells) have aspects in common with other tissue differentiation processes such as endothelial angiogenesis (protease, collagen, and noncollagen molecule secretion) [56] and specific features. Here we show that phenotypic changes that occur in maturing adipocytes are paralleled by expression of the respective genes.

#### Extracellular matrix remodeling

Matrix metalloproteinase-2 (*MMP-2 *[number 342, cluster 2]) was strongly upregulated during the entire process of adipocyte differentiation. Matrix metalloproteinase-2 can cleave various collagen structures and its inhibition can block adipogenesis [57]. Tissue inhibitor of metalloproteinase-2 (*Timp2 *[number 239, cluster 9]), a known partner of matrix metalloproteinase-2, which balances the activity of the proprotease/protease [58], was mainly upregulated. Decreased levels of tissue inhibitor of metalloproteinase-3 (number 81, cluster 10; upregulated at 6 hours and repressed after 12 hours) are associated with obese mice [59]. New collagen structures of overexpressed *Col6a2 *(number 11, cluster 9), *Col4a1 *(number 58, cluster 2) and *Col4a2 *(number 303, cluster 2) [60] are cross-linked by the lysyl oxidase (*Lox *[number 282, cluster 2]; upregulated during adipogenesis, which is contrary to findings reported by Dimaculangan and coworkers [61]). Strongly upregulated decorin (*Dcn *[number 137/623, cluster 7]) and osteoblast specific factor 2 (*Postn*/*Osf-2 *[number 183, cluster 7]), as well as proline arginine-rich end leucine-rich repeats (*Prelp *[number 73/484, cluster 3]; upregulated in the final stages of adipogenesis), attach the matrix to the cell. Matrillin-2 (*Matn2 *[number 12, cluster 9]; upregulated during adipogenesis) functions as adaptor for noncollagen structures [62], as does nidogen 2 (*Nid2 *[number 294, clusters 6 and 9]; increasingly upregulated). Secreted protein acidic and rich in cysteine/osteonectin (*SPARC *[number 67, cluster 9]; mainly upregulated) and SPARC-like 1 (*Sparcl1 *[number 154, cluster 9]; upregulated at 0 hours, 72 hours, 7days, and 14 days) can organize extracellular matrix remodeling, inhibit cell cycle progression, and induce cell rounding in cultured cells [63,64].

#### Reorganization of the cytoskeleton

Most cytoskeletal proteins are coexpressed in cluster 10 (not repressed from 6 to 12 hours) and might have a common regulatory mechanism. Transcription of actin α (*Acta1 *[number 445, cluster 10]) and actin γ (*Actg1 *[number 656, cluster 10]), tubulin α (*Tuba4 *[number 377, cluster 8]), and tubulin β (*Tubb5 *[number 110, cluster 8]) were found to diminish during differentiation, which is in agreement with other reports [65]. Myosin light chain 2 (*Mylc2b*/*Mylpf *[number 87/88/52/421, cluster 10]), and tropomyosin 1 and 2 (*Tpm1*/*Tpm2 *[number 74/68, cluster 10]) are members of the mainly repressed cluster 10. The downregulated transgelin 1 and 2 (*Tagln*/*Tagln2 *[number 114/242, cluster 10/8]) as well as fascin homolog 1 (*Fscn1 *[number 30, cluster 10]) are known actin-bundling proteins [66,67]. Apparently, their absence decreases the cross-linking of microfilaments in compact parallel bundles. Calponin 2 (*Cnn2 *[number 7, cluster 10]), a regulator of cytokinesis, is downregulated [68]. The insulin receptor and actin binding proteins filamin α and β (*Flna*/*Flnb *[number 506/632, cluster 10]) can selectively inhibit the mitogen-activated protein kinase signaling cascade of the insulin receptor [69]. Finally, the maintenance protein ankycorbin (*Rai14 *[number 59, cluster 10]) and the cross-linking protein actinin 1 (*Actn1 *[number 521, cluster 10]) share the mainly repressed expression profile. Tubulin γ 1 (*Tubg1 *[number 78, cluster 7]; upregulated during adipogenesis, about 42-fold at 6 hours) is not a component of the microtubules like *Tuba*/*Tubb*, but it plays a role in organizing their assembly and in establishing cell polarity [70]. Actinin 4 (*Actn4 *[number 185, cluster 9]; upregulated throughout adipogenesis) differs from *Actn1 *in its localization. Its expression leads to higher cell motility, and it can be translocated into the nucleus upon phosphatidylinositol 3-kinase inhibition [71]. Adducin 3γ (*Add3 *[number 50, cluster 9]; permanently upregulated) has different actin-associated cytoskeletal roles.

T-lymphoma invasion and metastasis 1 (*Tiam1 *[number 159, cluster 2]) is a guanine nucleotide exchange factor of the small GTPase Rac1, which regulates actin cytoskeleton, morphology and adhesion, and antagonizes RhoA signaling [72,73]. Additionally, the putative constitutive active Rho GTPase ras homolog gene family, member U/Wnt1 responsive Cdc42 homolog (*Rhou*/*Wrch-1 *[number 292, clusters 2 and 7]), which has no detectable intrinsic GTPase activity and very high nucleotide exchange capacity, leads to an phenotype of mature adipocyte [74,75]. Interplay between *Rhou *and *Tiam1*, which might reverse *Rhou *activity through Rac1 signaling [74], could be a mechanism for regulating cell morphology in adipogenesis.

In summary, the evidence presented above suggests that reduced replenishment of the cytoskeleton with building blocks and the strong transcriptional upregulation of modulating proteins, together with the extracellular remodeling, are responsible for the morphological changes that occur during differentiation of 3T3-L1 cells.

### Regulation of metabolic networks at the transcriptional level via key points of pathways

**Figure 5:**
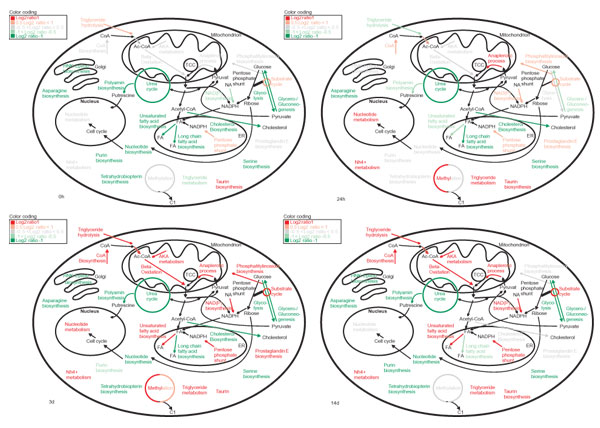
Temporal activation of metabolic pathways. Summarized is the activation of metabolic pathways at different time points (0 hours, 24 hours, 3 days, and 14 days) during fat cell differentiation. Color codes are selected according to expression levels of key enzymes in these pathways at distinct time points (red = upregulated; green = downregulated).

We next used the molecular atlas to derive novel biological insights from the global view of molecular processes. We analyzed transcriptionally regulated genes that are members of 36 different metabolic pathways. Within each pathway, we considered whether these transcriptionally regulated genes occupy key positions, such as a position at the pathway start, which is the typical rate-limiting step where the amount of enzyme is critical [76], or at some other point of regulation. We found that such key positions are occupied by transcriptionally regulated targets in 27 pathways (an overview is provided in Table 1). Those pathways that are strongly transcriptionally regulated at key points are illustrated in Figure 5 at the time points 0, 24 and 48 hours, and 14 days. For additional time points and images with more detailed information from all investigated pathways, see our website [20] and Additional data files Additional data file 31 and Additional data file 32.

In the following discussion we present the evidence for transcriptional regulation at key points for five selected metabolic pathways. Further information on other pathways can be found in Additional data files 31, 32, 33, 34, 35, 36, 37, 38 and on our website [20].

#### Biosynthesis of the important lipogenic cofactors CoA and NAD(P)+ are transcriptionally regulated at their key enzymes

Coenzyme A (CoA) is the carrier of the fatty acid precursor acetate/malonate [77,78]. Panthotenate kinase 3 (*Pank3 *[number 140, cluster 6]; about eightfold upregulated) is responsible for the first and rate-limiting step in converting panthotenate to CoA [79]. Nicotinamide adenine dinucleotide phosphate (reduced form; NADPH) is necessary in reductive reactions for fatty acid synthesis. Pre-B-cell colony-enhancing factor (*Visfatin*/*Pbef1 *[number 327, cluster 9]; strongest upregulated in the last three points of the time course in parallel with the emergence of fat droplets) is the rate-limiting enzyme in NAD(P)+ biosynthesis [38,80]. For reduction of NADP+ to NADPH, two major mechanisms are responsible: the pentose phosphate shunt and the tricarboxylate transport system. Hexose-6P dehydrogenase (*H6pd *[number 533, cluster 9]; upregulated throughout adipogenesis) is the rate limiting enzyme of the pentose phosphate shunt in the endoplasmic reticulum and provides NADPH to its lumen [81]. In the cytosolic pendant in the pentose phosphate shunt, the transaldolase (*Taldo1 *[number 160, cluster 3]) is repressed at early stages and is about threefold upregulated at the end of 3T3-L1 differentiation. This expression change appears to switch the shunt between ribose-5-phosphate (for nucleic acid synthesis) and NADPH (for fatty acid production) synthesis at early and late time points, respectively. A similar expression profile is observed for the cytosolic NADP-dependent malic enzyme (*Mod1 *[number 76, cluster 3]) and the citrate transporter (*Slc25a1*/*Ctp1 *[number 209, cluster 3]). Both are part of the tricarboxylate transport system through the mitochondrial membrane. Transcription of the anaplerotic pyruvate carboxylase (*Pcx *[number 149, cluster 6]; activated by acetyl-CoA) is increasingly upregulated up to 16-fold toward the final two time points.

#### Fatty acid modification and assimilation is transcriptionally regulated at the rate-limiting steps

The transcriptional expression of stearoyl-CoA desaturase 1 (*Scd1 *[number 305, cluster 6]), which catalyzes the rate-limiting reaction of monounsaturated fatty acid synthesis and which is an important marker gene of adipogenesis [82,83], is downregulated at induction but increases up to 60-fold with advancing adipogenesis. In contrast to previous reports [82], we found that the gene for elongation of long-chain fatty acid (*Elovl6 *[number 162, cluster 12]) protein, which may be the rate-limiting enzyme of long chain elongation to stearate [84], is not overexpressed in differentiated 3T3-L1 cells as in adipose tissue. *Elovl6 *appears repressed during the entire process of adipogenesis in 3T3-L1 cells. Expression of lipoprotein lipase (*Lpl *[number 14, cluster 6]), the rate-limiting enzyme of extracellular triglyceride-rich lipoprotein hydrolyzation and triglyceride assimilation [85-87], increases with time up to 21-fold in differentiated adipocytes.

#### Transcriptional regulation of triglyceride and fatty acid degradation is performed at key points

Adipose triglyceride lipase (*Pnpla2*/*Atgl *[number 157, cluster 6]) executes the initial step in triglyceride metabolism [35]. Its expression increases strongly with differentiation progression. Acyl-CoA dehydrogenases (*Acadm*/*Acadsb *[number 153/220, clusters 6 and 9]) [88], the rate-limiting enzymes of medium, short and branched chain β-oxidation, are strongly upregulated in the final four time points. In contrast, the acyl-CoA dehydrogenase (*Acadvl*) of very long chain fatty acids is not in the set of distinctly differentially regulated genes, and exhibits some upregulation at the final two time points. This difference in expression might shift the enrichment from short and medium to long chain fatty acids during adipogensis. Branched chain ketoacid dehydrogenase E1 (*Bckdha *[number 193, clusters 3 and 9]) is the rate-limiting enzyme of leucine, valine, and isoleucine catabolism and is known to be inhibited by phosphorylation [89]. Its gene shares a similar expression profile with the *Acad *genes. The elevated degradation of amino acids allows conversion to fatty acids through acetyl-CoA.

#### Several important nucleotide biosynthetic pathway enzymes follow a cell cycle specific expression profile (strongly repressed except between 12 and 24 hours)

Phosphoribosylpyrophosphate amidotransferse (*Ppat *[number 287, cluster 11]) [90] is rate-limiting for purin production. Deoxycytidine kinase (*Dck *[number 363, cluster 8]) is the rate-limiting enzyme of deoxycytidine (dC), deoxyguanosine (dG) and deoxyadenosine (dA) phosphorylation [91-93]. Ribonucleotide reductase M2 (*Rrm2 *[number 448, cluster 8]) converts ribonucleotides to desoxyribonucleotides [94,95]. Additionally, thymidine kinase 1 (*Tk1 *[number 165, cluster 5]) and dihydrofolate reductase (*Dhfr *[number 161, cluster 5/8]) play important roles in dT and purin biosynthesis during the cell cycle. In contrast, purin degradation is about sixfold upregulated between 6 and 72 hours by the rate-limiting xanthine dehydrogenase (*Xdh *[number 361, cluster 2]) [96,97]. These findings are in concordance with those of a previous study [22], which showed that mitotic clonal expansion is a prerequisite for differentiation of 3T3-L1 preadipocytes into adipocytes. After induction of differentiation, the growth-arrested cells synchronously re-enter the cell cycle and undergo mitotic clonal expansion, as monitored by changes in cellular DNA content [22]. In accord with this experimental evidence, we observed changes in cell cycle genes, most of which were in clusters 5 and 8 (see our website [20] and Additional data file 37).

#### Cholesterol biosynthesis is regulated by expression of key steps and whole pathway segments

The synthesis of the early precursor molecule 3-hydroxy-3-methylglutaryl (HMG)-CoA, which might be also used in other metabolic pathways, is transcriptionally controlled at the key enzymes HMG-CoA synthase (*Hmgcs1 *[number 178, cluster 4]; repressed except in terminal stages) and HMG-CoA reductase (*Hmgcr *[number 619, cluster 12]; always repressed), which is the rate-limiting enzyme of the cholesterol and mevalonate pathway [98,99]. After the step of isopentenylpyrophosphate synthesis, cholesterol biosynthesis genes are coexpressed in cluster 4.

### Correspondence between coexpression and coregulation

To determine whether coexpressed genes are also coregulated, we analyzed the available promoter sequences of the 780 ESTs. Promoter sequences could be retrieved for 357 genes. Most ESTs are sequenced from the 3' end, and hence it is easier to retrieve the 3'-UTR. Retrieval of promoters is more difficult than retrieval of the 3'-UTR because of experimental problems in extracting full-length cDNAs (and hence transcription start sites) and insufficient computational methods for identifying beginning of the 5'-UTR. We analyzed the occurrences of the binding sites of all transcription factors in vertebrates from the TRANSFAC database. Based on statistical analyses, among transcription factors with binding site motifs described in TRANSFAC [100] those listed in Table 2 are the most promising candidates for further functional studies on transcriptional regulation.

One example of a functional transcription factor binding site is SREBP-1 in cluster 4. A comparison among clusters showed that cluster 4 has significantly more genes with a SREBP-1 (SRE and E-box motifs [101]) binding site than all other clusters (*P *= 0.0484, Fisher's exact test; Table 3). Similarly, a putative SREBP-1 regulatory region is significantly more frequent in the promoters of the genes in cluster 4 compared with all unique sequences in the PromoSer database (*P *< 0.0289; PromoSer contains 22,549 promoters of 12,493 unique sequences). For a subset of the genes in cluster 4 with predicted SREBP-1 binding site (most genes of the cholesterol biosynthesis pathway), transcriptional regulation with SREBP-1 has been experimentally proven [102].

Surprisingly, binding sites for the key regulators of adipogenesis, namely PPAR and C/EBP, are not significantly over-represented in any of the promoters of the coexpressed genes. We generated a novel matrix for PPAR using 22 experimentally verified binding sites from the literature and analyzed the promoters of the coexpressed genes and all PromoSer promoters. Again, using this matrix the PPAR binding sites were not significantly over-represented.

### Genomic position of coexpressed genes

**Figure 6:**
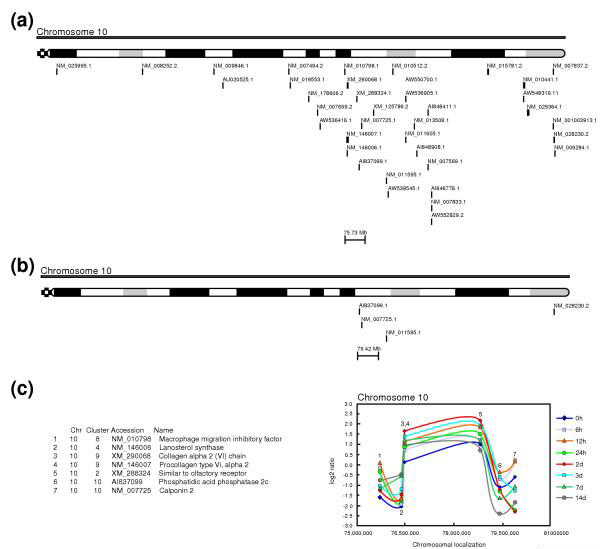
Chromosomal localization analysis for ESTs found to be differentially expressed during fat cell differentiation. Chromosomal localization analysis for chromosome 10 from 780 ESTs shown to be more than two times upregulated or downregulated in a minimum of four time points during adipocyte differentiation. (**a**) Mapped ESTs to chromosome 10. (**b**) ESTs from cluster 10 mapped to chromosome 10. (**c**) Relative gene expression levels (log_2 _ratios) at different time points for seven ESTs mapped within a genomic interval of 5 Mb from chromosome 10. EST, expressed sequence tag.

Finally, we considered whether coexpressed genes also colocalize on the chromosomes. In a broad genomic interval (5 megabases [Mb]) on each mouse chromosome we mapped the ESTs from each cluster. Unexpectedly, our data do not support the observation of the highly significant correlation in the expression and genomic positioning of the genes. A typical example of mapped ESTs to chromosome 10 is illustrated in Figure 6, showing that expression levels of colocalized ESTs are divergent because only two mapped ESTs are members of the same cluster.

Additionally, we analyzed the genomic position of 5,205 ESTs that exhibited significant differentially expression between time points (*P *< 0.05; one-way ANOVA). These ESTs were grouped in 12 clusters, and we then searched for regions with three or more members in a genomic interval of 500 kilobases (kb). On average, 7 ± 5% of the ESTs from one cluster were colocalized. Comprehensive results of this analysis are accessible within the supplementary website [20] and Additional data files 42, 43, 44, 45.

In summary, these data do not provide evidence that colocalized genes in the genomic sequence are subject to the same transcriptional regulation (coexpression), as indicated by examples for different processes in other studies [103].

## Discussion

The data presented here and the functional annotation considerably extend upon previous microarray analyses of gene expression in fat cells [8-14] and demonstrate the extent to which molecular processes can be revealed by global expression profiling in mammalian cells. Our strategy resulted in a molecular atlas of fat cell development and provided the first global view of the underlying biomolecular networks. The molecular atlas and the dissection of molecular processes suggest several important biological conclusions.

First, the data support the notion that there are hundreds of mouse genes involved in adipogenesis that were not previously linked to this process. Out of the 780 selected genes, 326 were not shared with previous studies [8,9,12], suggesting that our view of this process is far from complete. Using microarrays enriched with developmental ESTs, we were able not only to identify new components of the transcriptional network but also to map the gene products onto molecular pathways. The molecular atlas we developed is a unique resource for deriving testable hypothesis. For example, we have identified several differentially expressed genes, including recently discovered gene products (Pnpla2, Pbef1, Mest) and transcription factors not previously detected in microarray screens (Zhx3 and Zfp367).

Second, from our global analysis of the potential role of miRNAs in fat cell differentiation, we were able to predict potential target genes for miRNAs in 71% of the 395 genes with a unique 3'-UTR that were differentially expressed during adipocyte differentiation. The distribution of predicted miRNA targets indicated that one miRNA may regulate many genes and that one gene can be regulated by a number of miRNAs. The function of the potential target genes was diverse and included transcription factors, enzymes, transmembrane proteins, and signaling molecules. Genes with the lowest number of over-represented miRNA motifs were cell cycle genes (clusters 5 and 8), whereas genes grouped in cluster 9 exhibited the most over-represented miRNA motifs in relation to the matches in the control set of all available 3'-UTR sequences. Genes in cluster 9 exhibited high expression values at time point 0 and may include genes relevant to the transition from pre-confluent to confluent cells. Genes in cluster 9 also represent molecular components that are involved in other cell processes, including extracellular matrix remodeling, transport, metabolism, and fat cell development (for example, *Foxo1 *[44,45]). Genes in other clusters exhibited varying percentages of over-represented miRNA motifs and can be associated with diverse biological processes (Additional data file 6). As an example of functional miRNA targets, we showed that one signaling molecule of the ras family is a potential target of miRNAs, which is consistent with a previous observation in humans, in whom it was shown that the human RAS oncogene is regulated by the let-7 miRNA. This example indicates that the present analysis provides promising candidates ranked according to their significance of over-representation and the number of different miRNAs that might regulate these targets in the specific context of adipocyte differentiation. It should be noted that our analysis included only known miRNAs, suggesting that the number of target sites can be even higher. This striking observation could have implications for post-transcriptional regulation of other developmental processes. Microarrays for the analysis of miRNA expression are becoming available and future studies will shed light on the role of miRNAs in the context of cell differentiation.

Third, we were also able to characterize the mechanisms and gene products involved in the phenotypic changes of pre-adipocytes into mature adipocytes. Although the number of selected genes in this study was limited, we characterized gene products for extracellular matrix remodeling and cytoskeletal changes during adipogenesis. Other molecular components involved in these processes can be identified by mapping the characterized gene products onto curated pathways [30] and selecting missing candidates for further focused studies. Notably, most of the cytoskeletal proteins are coexpressed in cluster 10 and might have a common regulatory mechanism. Further computational and experimental analyses are needed to verify this hypothesis.

In addition to new information about fat cell development, our comprehensive analysis has provided new general biological insights that could only be derived from such a global analysis. First, we were able to examine at what points metabolic pathways were regulated. The global view of biological processes and networks derived from expression profiles showed that the metabolic networks are transcriptionally regulated at key points, usually the rate-limiting steps. This was the case in 27 out of the 36 metabolic pathways analyzed in this study. During the development of mature adipocytes from pre-adipocytes, distinct metabolic pathways are activated and deactivated by this molecular control mechanism. For example, at the beginning nucleotide metabolism is activated because the cells undergo clonal expansion and one round of the cell cycle (see out website [20] and Additional data file 37). At the end of development, major metabolic pathways for lipid metabolism are upregulated, including β-cell oxidation and fatty acid synthesis (Figure 5). Cell development is a dramatic process in which the cell undergoes biochemical and morphological changes. In our study signals at every time point could be detected from more than 14,000 ESTs. Thus, regulating metabolic networks at key points represents an energy efficient way to control cellular processes. Metabolic networks might be activated/inactivated in a similar manner in other types of cellular differentiation, such as myogenesis or osteogenesis. It is intriguing to speculate that signaling networks are also transcriptionally regulated at key points. However, as opposed to the metabolic networks, it is difficult to verify this hypothesis because the key points are not clearly identifiable due to both the interwoven nature and the partial incompleteness of the signaling pathways.

A second general biological insight derived from our global analysis is that we found that many genes were upregulated by well known transcription factors that nevertheless lacked anything resembling the established upstream promoter consensus sites. Over-represented binding sites for key regulators such as PPAR and C/EBP were not detectable with the TRANSFAC matrices. Even the use of a matrix based on all currently available experimentally validated sequences, such as PPAR, did not result in a significant hit. Hence, only few sequences contain this motif in their promoters. These results demonstrate either that much more sophisticated methods must be developed or that there are many cases where the current methods do not perform well because other aspects such as chromatin determine the recognition site.

A third general piece of information derived from our global analysis is the finding that coexpressed genes in fat cell development are not clustered in the genome. Previous studies identified a number of such cases in a range of organisms, including yeast [104], worms [105], and flies [106]. This observation of significant correlation in the expression and genomic position of genes was recently reported in the mouse [103]. In the present study we could not identify groups of genes with similar expression profiles, for instance within the same cluster within 5 Mb regions on the chromosomes. Our results suggest that such clustering may not be as widespread as may be presumed by extrapolating from previous studies. However, coexpressed genes could have distant locations and still be spatially colocalized due to DNA looping and banding, as was recently shown in a microscopy study [107]. A higher order chromatin structure of the mammalian transcriptome is an emerging concept [108], and new methods are required to examine the correlation between gene activity and spatial positioning.

The biological insights gained in this study were only possible with in-depth bioinformatics analyses based on segment and domain predictions. Distribution of GO terms permits a first view of the biological processes, molecular functions, and cellular components. However, in our work more than 40% of the ESTs could not be assigned to GO terms. Moreover, detailed information about the specific functions cannot be extracted. For example, the GO term 'DNA binding' could be specified by 'zinc-finger domain binding protein' only by in-depth analyses. Hence, de novo functional annotation of ESTs using integrated prediction tools and subsequent curation of the results based on the available literature is not only necessary to complete the annotation process but also to reveal the actual biological processes and metabolic networks. Although the number of protein sequences to which a GO term can be assigned is steadily increasing, specific and detailed annotation is only possible with de novo functional annotation.

## Conclusion

In the present study we demonstrate that, despite the limitations due to mRNA abundance (many thousands of genes are never transcribed above threshold) and insufficient sensitivity, large-scale gene expression profiling in conjunction with sophisticated bioinformatics analyses can provide not only a list of novel players in a particular setting but also a global view on biological processes and molecular networks.

## Materials and methods

### cDNA microarrays

The microarray developed here contains 27,648 spots with mouse cDNA clones representing 16,016 different genes (UniGene clusters). These include developmental clones (the 15 K NIA cDNA clone set from National Institute of Aging, US National Institutes of Health) and the 11 K clones from different brain regions in the mouse (Brain Molecular Anatomy Project [BMAP]). Moreover, 627 clones for adipose-related genes were selected using the TIGR Mouse Gene Index Build 5.0 [19]. These cDNA clones were obtained from the IMAGE consortium (Research Genetics, Huntsville, AL, USA). The inserts of the NIA and BMAP clones were sequence verified (insert size about 1-1.5 kb). All PCR products were purified using size exclusion vacuum filter plates (Millipore, Billerica, MA, USA) and spotted onto amino-silanated glass slides (UltraGAPS II; Corning, Corning, NY, USA) in a 4 × 12 print tip group pattern. As spotting buffer 50% dimethyl sulfoxide was used. Negative controls (genomic DNA, genes from *Arabidopsis thaliana*, and dimethyl sulfoxide) and positive controls (Cot1-DNA and salmon sperm DNA) were included in each of the 48 blocks. Samples were bound to the slides by ultraviolet cross-linking at 200 mJ in a Stratalinker (Stratagene, La Jolla, CA, USA).

### Cell culture

3T3-L1 cells (American Type Culture Collection number CL-173) were grown in 100 mm diameter dishes in Dulbecco's modified Eagle's medium supplemented with 10% fetal bovine serum, 100 units/ml penicillin, 100 μg/ml streptomycin, and 2 mmol/l L-glutamine in an atmosphere of 5% carbon dioxide at 37°C. Two days after reaching confluence (day 0), cells were induced to differentiate with a two-day incubation of a hormone cocktail [109,110] (100 μmol/l 3-iso-butyl-1-methylxanthine, 0.25 μmol/l dexamethasone, 1 μg/ml insulin, 0.16 μmol/l pantothenic acid, and 3.2 μmol/l biotin) added to the standard medium described above. After 48 hours (day 2), cells were cultured in the standard medium in the presence of 1 μg/ml insulin, 0.16 μmol/l pantothenic acid, and 3.2 μmol/l biotin until day 14. Nutrition media were changed every second day.

Three independent cell culture experiments were performed. Cells were harvested and total RNA was isolated at the preconfluent stage and at eight time points (0, 6, 12 and 24 hours, and 3, 4, 7 and 14 days) with TRIzol reagent (Invitrogen-Life Technologies; Carlsbad, CA, USA) [111]. For each independent experiment, RNA was pooled from three different culture dishes for each time point and from 24 dishes at the preconfluent stage used as reference. The quality of the RNA was checked using Agilent 2100 Bioanalyzer RNA assays (Agilent Technologies, Palo Alto, CA, USA) by inspection of the 28S and 18S ribosomal RNA intensity peaks.

### Labeling and hybridization

The labeling and hybridization procedures used were based on those developed at the Institute for Genomic Research [112] and detailed protocols can be viewed on the supplementary website [20]. Briefly, 20 μg total RNA from each time point was reverse transcribed in cDNA and indirectly labeled with Cy5 and 20 μg RNA from the preconfluent stage (reference) was indirectly labeled with Cy3, respectively. This procedure was repeated with reversed dye assignment. Slides were prehybridized with 1% bovine serum albumen. Then, 10 μg mouse Cot1 DNA and 10 μg poly(A) DNA was added to the labeled cDNA samples and pair-wise cohybridized onto the slide for 20 hours at 42°C. Following washing, slides were scanned with a GenePix 4000B microarray scanner (Axon Instruments, Sunnyvale, CA, USA) at 10 μm resolution. Identical photo multiplier voltage settings were used in the scanning of the corresponding dye-swapped hybridized slides. The resulting TIFF images were analyzed with GenePix Pro 4.1 software (Axon Instruments).

### Data preprocessing and normalization

Data were filtered for low intensity, inhomogeneity, and saturated spots. To obtain expression values for the saturated spots, slides were scanned a second time with lower photomultiplier tube settings and reanalyzed. All spots of both channels were background corrected (by subtraction of the local background). Different sources of systematic (sample, array, dye, and gene effects) and random errors can be associated with microarray experiments [113]. Nonbiological variation must be removed from the measurement values and the random error can be minimized by normalization [114,115]. In the present study, gene-wise dye swap normalization was applied. Genes exhibiting substantial differences in intensity ratios between technical replicates were excluded from further analysis based on a two standard deviation cutoff. The resulting ratios were log_2 _transformed and averaged over three independent experiments. The expression profiles were not rescaled in order to identify genes with high expression values. All experimental parameters, images, and raw and transformed data were uploaded to the microarray database MARS [116] and submitted via MAGE-ML export to a public repository (ArrayExpress [117], accession numbers A-MARS-1 and E-MARS-2). Differentially expressed genes were first identified using one-way ANOVA (*P *< 0.05). They were then subjected to a more stringent criterion; specifically, we considered only those genes with a complete temporal profile that were more than twofold upregulated or downregulated at a minimum of four time points. The twofold cutoff for differentially expressed genes was estimated by applying the significance analyses of microarrays method [118] to the biological replicates and assuming false discovery rate of 5%. In order to capture the dynamics of various processes, only ESTs differentially expressed in at least half of the time points were selected. Data preprocessing was performed with ArrayNorm [119].

### Real-time RT-PCR

Microarray expression results were confirmed with RT-PCR. cDNA was synthesized from 2.5 μg total RNA in 20 μl using random hexamers and SuperScript III reverse transcriptase (Invitrogen, Carlsbad, CA, USA). The design of LUX™ primers for *Pparg*, *Lpl*, *Myc*, *Dec*, *Ccna2*, and *Klf9 *was done using the Invitrogen web service (for sequences, see Additional data file 9 and our website [20]). Quantitative RT-PCR analyses for these genes were performed starting with 50 ng reverse transcribed total RNA, with 0.5× Platinum Quantitative PCR SuperMix-UDG (Invitrogen, Carlsbad, CA, USA), with a ROX reference dye, and with a 200 nmol/l concentration of both LUX™ labeled sense and antisense primers (Invitrogen, Carlsbad, CA, USA) in a 25 μl reaction on an ABI PRISM 7000 sequence detection system (Applied Biosystems, Foster City, CA, USA). To measure PCR efficiency, serial dilutions of reverse transcribed RNA (0.24 pg to 23.8 ng) were amplified. Ribosomal 18S RNA amplifications were used to account for variability in the initial quantities of cDNA. The relative quantification for any given gene with respect to the calibrator (preconfluent stage) was determined using the ΔΔC_t _method and compared with the normalized ratios resulting from microarray experiments.

### Clustering and gene ontology classification

Common unsupervised clustering algorithms [120] were used for clustering expression profiling of 780 selected ESTs, according to the log ratios from all time points. Using hierarchical clustering the boundaries of the clusters were not clearly separable and required arbitrary determination of the branching point of the tree, whereas the results of the clustering using self-organized maps led to clusters with highly divergent number of ESTs (between 3 and 242). We have therefore used the k means algorithm [121] and Euclidean distance. The number of clusters was varied from k = 1 to k = 20, and predictive power was analyzed with the figure of merit [122]. Subsequently, k = 12 was found to be optimal. To evaluate the results of the k means clustering, principal component analysis [123] was applied and exhibited low intracluster distances and high intercluster dissimilarities. GO terms and GO numbers for molecular function, biological process, and cellular components were derived from the Gene Ontology database (Gene Ontology Consortium) using the GenPept/RefSeq accession numbers for annotated proteins encoded by selected genes (ESTs). All cluster analyses and visualizations were performed using Genesis [124].

### *De novo* annotation of ESTs

For each of the 780 selected EST sequences, we attempted to find the corresponding protein sequence. Megablast [125] searches (word length w = 70, percentage identity *p* = 95%) against nucleotide databases (in the succession of RefSeq [126,127], FANTOM [128], UniGene [129], nr GenBank, and TIGR Mouse Gene Index [19] until a gene hit was found) were carried out. For the ESTs still remaining without gene assignment, new Megablast searches were conducted with the largest compilation of RefSeq (including the provisional and automatically generated records [126,127]). If an EST remained unassigned, then the whole procedure was repeated with blastn [130]. In addition, a blastn search against the ENSEMBL mouse genome [131] was performed, and ESTs with long stretches (>100 base pairs) of unspecified nucleotides (N) were excluded.

All protein sequences were annotated *de novo *with academic prediction tools that are integrated into ANNOTATOR, a novel protein sequence analysis system [132]: compositional bias (SAPS [133], Xnu, Cast [134], GlobPlot 1.2 [135]); low complexity regions (SEG [136]); known sequence domains (Pfam [137], Smart [138], Prosite and Prosite pattern [139] with HMMER, RPS-BLAST [140], IMPALA [141], PROSITE-Profile [139]); transmembrane domains (HMMTOP 2.0 [142], TOPPRED [143], DAS-TMfilter [144], SAPS [133]); secondary structures (impCOIL [145], Predator [146], SSCP [147,148]); targeting signals (SIGCLEAVE [149], SignalP-3.0 [150], PTS1 [151]); post-translational modifications (big-PI [152], NMT [153], Prenylation); a series of small sequence motifs (ELM, Prosite patterns [139], BioMotif-IMPlibrary); and homology searches with NCBI blast [130]. Further information was retrieved from the databases of Mouse Genome Informatics [154] and LocusLink [126].

### Promoter analysis

The promoters were retrieved from PromoSer database [155] through the gene accession number. PromoSer contains 22,549 promoters for 12,493 unique genes. Nucleotides from 2,000 upstream and 100 downstream of the transcription start site were obtained. With an implementation of the MatInspector algorithm [156], the Transfac matrices [100] were checked for binding sites in the promoter regions with a threshold for matrix similarity of 0.85. We counted the number of those gene sequences that were found to carry a predicted transcription factor binding site. As a reference set all unique genes of the PromoSer were reanalyzed. A one-sided χ^2 ^test and a one-sided Fisher's exact test (to improve the statistics for view counts) were performed with the statistical tool R [157] to determine the clusters with a higher affinity for a transcription factor.

### Identification of miRNA target sites in 3'-UTR

All available 3'-UTR sequences (21,396) for mouse genes were derived with EnsMart [158], using Ensembl gene build for the NCBI m33 mouse assembly. 3'-UTRs for unique genes represented by the 780 selected ESTs were extracted using Ensembl transcript ID. A total of 234 mouse miRNA sequences were derived from the Rfam database [159]. The 3'-UTR sequences were searched for antisense matches to the designated seed region of each miRNA (bases 1-8, 2-8, 1-9, and 2-9 starting from the 5' end). Significantly over-represented miRNA motifs in each cluster in comparison with the remaining motifs in the whole 3'-UTR sequence set were determined using the one-sided Fisher's exact test (significance level: *P *< 0.05) and miRNA targets of all clusters were analyzed for significantly over-represented miRNAs.

### Chromosomal localization analysis

RefSeq sequences for 780 selected ESTs, shown to be more than two times upregulated or downregulated in a minimum of four time points during adipocyte differentiation and clustered according their expression profiles, were mapped onto the chromosomes from the NCBI *Mus musculus *genome (build 33) using ChromoMapper 2.1.0 software [160] based on MegaBlast with the following parameters: 99% identity cutoff, word size 32, and E-value (0.001). Colocalized sequences of all selected ESTs and from each of the 12 clusters within a 5 Mb genomic interval were identified. Within the 5Mb genomic intervals of each chromosome with the highest density of mapped ESTs, relative gene expression levels (log2 ratios) of these ESTs at different time points were related to the genomic localization.

## Additional data files

The following additional data are provided with the online version of this article: A spot map for the array design (Additional data file 1); a fasta file containing the EST sequences used for the array (Additional data file 2); an Excel file containing expression values for the 780 selected ESTs (Additional data file 3); an Excel file containing expression values for the 5205 ESTs filtered with ANOVA (Additional data file 4); GenePix result files containing raw data (Additional data file 5); images showing the distribution of gene ontology (Additional data file 6); a table listing relevant proteins (Additional data file 7); a fasta file containing sequences of the relevant proteins (Additional data file 8); a pdf file containing real-time RT-PCR data (Additional data file 9); a table including statistical analysis of independent experiments (Additional data file 10); figure showing a comparison with GeneAtlas (Additional data file 11); a table including expression levels from the present study and GeneAtlas (Additional data file 12); figure showing a comparison with the data set reported by Soukas and coworkers [8]Additional data file 13); figure showing a comparison with the data set reported by Ross and coworkers [9] (Additional data file 14); figure showing a comparison with the data set reported by Burton and coworkers [12] (Additional data file 15); a table including ESTs unique to the present study (Additional data file 16); a figure showing genes with miRNA motifs in 3'-UTR (Additional data file 17); a figure illustrating the significant over-representation of miRNA motifs in the 3'-UTR of genes in each cluster (Additional data file 18); figures showing the significant over-representation of miRNA motifs in the 3'-UTR from genes in each cluster (Additional data files 19, 20, 21, 22, 23, 24, 25, 26, 27, 28, 29); a table including over-represented miRNA motifs in the 3'-UTR from genes in the set of 780 selected ESTs (Additional data file 30); text describing regulation of metabolic pathways (Additional data file 31); figure showing regulation of metabolic pathways by key points (Additional data file 32); figure showing the cellular localization of gene products involved in metabolism and their gene expression at different time points (Additional data file 33); figure showing the cellular localization of gene products involved in other biological processes and their gene expression at different time points (Additional data file 30); text describing signaling networks (Additional data file 35); text file describing extracellular matrix remodeling and cytoskeleton reorganization (Additional data file 36); figure showing cell cycle processes (Additional data file 37); figure showing the cholesterol pathway (Additional data file 38); a list of experimental verified binding site for PPAR:RXR and the derived position weight matrix (Additional data file 39); text file containing TRANSFAC matrices for vertebrates (Additional data file 40); a file showing the promoter sequences in fasta format (Additional data file 41); figure showing cluster-wise mapping of 780 ESTs to all chromosomes (Additional data file 42); figure showing expression of colocalized ESTs for each cluster (Additional data file 43); an Excel file showing a statistical analysis of colocalized ESTs for 780 selected ESTs (Additional data file 44); and an Excel file showing a statistical analysis of colocalized ESTs for 5,502 ANOVA selected ESTs (Additional data file 45).

## Supplementary Material

Additional data file 1 A spot map for the array design Click here for file

Additional data file 2 A fasta file containing the EST sequences used for the array Click here for file

Additional data file 3 An Excel file containing expression values for the 780 selected ESTs Click here for file

Additional data file 4 An Excel file containing expression values for the 5205 ESTs filtered with ANOVA Click here for file

Additional data file 5 GenePix result files containing raw data Click here for file

Additional data file 6 Images showing the distribution of gene ontology Click here for file

Additional data file 7 A table listing relevant proteins Click here for file

Additional data file 8 A fasta file containing sequences of the relevant proteins Click here for file

Additional data file 9 A pdf file containing real-time RT-PCR data Click here for file

Additional data file 10 A table including statistical analysis of independent experiments Click here for file

Additional data file 11 A figure showing a comparison with GeneAtlas Click here for file

Additional data file 12 A table including expression levels from the present study and GeneAtlas Click here for file

Additional data file 13 A figure showing a comparison with the data set reported by Soukas and coworkers [8] Click here for file

Additional data file 14 A figure showing a comparison with the data set reported by Ross and coworkers [9] Click here for file

Additional data file 15 A figure showing a comparison with the data set reported by Burton and coworkers [12] Click here for file

Additional data file 16 A table including ESTs unique to the present study Click here for file

Additional data file 17 A figure showing genes with miRNA motifs in 3'-UTR Click here for file

Additional data file 18 A figure illustrating the significant over-representation of miRNA motifs in the 3'-UTR of genes in each cluster Click here for file

Additional data file 19 A figure showing the significant over-representation of miRNA motifs in the 3'-UTR from genes in cluster 1 Click here for file

Additional data file 20 A figure showing the significant over-representation of miRNA motifs in the 3'-UTR from genes in cluster 2 Click here for file

Additional data file 21 A figure showing the significant over-representation of miRNA motifs in the 3'-UTR from genes in cluster 3 Click here for file

Additional data file 22 A figure showing the significant over-representation of miRNA motifs in the 3'-UTR from genes in cluster 4 Click here for file

Additional data file 23 A figure showing the significant over-representation of miRNA motifs in the 3'-UTR from genes in cluster 6 Click here for file

Additional data file 24 A figure showing the significant over-representation of miRNA motifs in the 3'-UTR from genes in cluster 7 Click here for file

Additional data file 25 A figure showing the significant over-representation of miRNA motifs in the 3'-UTR from genes in cluster 8 Click here for file

Additional data file 26 A figure showing the significant over-representation of miRNA motifs in the 3'-UTR from genes in cluster 9 Click here for file

Additional data file 27 A figure showing the significant over-representation of miRNA motifs in the 3'-UTR from genes in cluster 10 Click here for file

Additional data file 28 A figure showing the significant over-representation of miRNA motifs in the 3'-UTR from genes in cluster 11 Click here for file

Additional data file 29 A figure showing the significant over-representation of miRNA motifs in the 3'-UTR from genes in cluster 12 Click here for file

Additional data file 30 A table including over-represented miRNA motifs in the 3'-UTR from genes in the set of 780 selected ESTs Click here for file

Additional data file 31 Text describing regulation of metabolic pathways Click here for file

Additional data file 32 A figure showing regulation of metabolic pathways by key points Click here for file

Additional data file 33 Figure showing the cellular localization of gene products involved in metabolism and their gene expression at different time points Click here for file

Additional data file 34 Figure showing the cellular localization of gene products involved in other biological processes and their gene expression at different time points Click here for file

Additional data file 35 Text describing signaling networks Click here for file

Additional data file 36 A text file describing extracellular matrix remodeling and cytoskeleton reorganization Click here for file

Additional data file 37 A figure showing cell cycle processes Click here for file

Additional data file 38 A figure showing the cholesterol pathway Click here for file

Additional data file 39 A list of experimental verified binding site for PPAR:RXR and the derived position weight matrix Click here for file

Additional data file 40 Text file containing TRANSFAC matrices for vertebrates Click here for file

Additional data file 41 A file showing the promoter sequences in fasta format Click here for file

Additional data file 42 A file showing the promoter sequences in fasta format Click here for file

Additional data file 43 A figure showing clusterwise mapping of 780 ESTs to all chromosomes Click here for file

Additional data file 44 An Excel file showing a statistical analysis of colocalized ESTs for 780 selected ESTs Click here for file

Additional data file 45 An Excel file showing a statistical analysis of colocalized ESTs for 5,502 ANOVA selected ESTs Click here for file

## Figures and Tables

**Table 1:** Activated metabolic pathways during adipocyte differentiation and their key enzymes (rate limiting steps)

Pathway	Enzyme/Protein name	Accession number	Number	Cluster
Urea cycle and arginine-citrulline cycles	Arginine succinate synthase	NP_031520	128	1/10
Phosphatidylinositol	Phosphatidylinositol 3-kinase, regulatory subunit, polypeptide 1	XP_127550	446	7
	Myoinositol 1-phosphate synthase A1	NP_076116	156	8
Cholesterol biosynthesis/keto-body synthesis	3-hydroxy-3-methylglutaryl-CoA synthase 1	NP_666054	178	4
	3-hydroxy-3-methylglutaryl-CoA reductase	XP_127496	619	12
Triglyceride hydrolysis (fatty acid assimilation)	Lipoprotein lipase (LPL)	NP_032535	14	6
β-Oxidation	Acetyl-CoA dehydrogenase (Acad)	NP_780533	61	6
	Acetyl-CoA dehydrogenase, medium chain (Acadm)	NP_031408	153	6/9
	Isovaleryl-CoA dehydrogenase (Acad)	Mm.6635	510	6
	Acyl-CoA dehydrogenase, short/branched chain (Acadsb)	NP_080102	220	9
Triglyceride metabolism	Adipose triglyceride lipase (Pnpla2/Atgl)	NP_080078	157	6
CoA biosynthesis	Pantothenate kinase 3	NP_666074	140	6
Anaplerotic processes	Pyruvate carboxylase	NP_032823	149	6
Branched chain amino acid metabolism (AKA metabolism)	Branched chain ketoacid dehydrogenase E1, α polypeptide	NP_031559	193	3/9
Methylation	S-adenosylhomocysteine hydrolase	NP_057870	66	8
	Methionine adenosyltransferase II, α	NP_663544	350	2
Unsaturated fatty acid biosynthesis	Stearoyl-CoA desaturase 1	NP_033153	305	6
Nucleotide metabolism	Xanthine dehydrogenase	NP_035853	361	2
Taurin biosynthesis	Cysteine dioxygenase	NP_149026	271	7
NH_4_^+ ^metabolism/glutamate	Glutamate-ammonia ligase (glutamine synthase)	NP_032157	318	7
Glycolysis	Pyruvate kinase 3	NP_035229	247	8
Substrate cycle (glycolysis/gluconeogenesis)	Fructose bisphosphatase 2	NP_032020	175	9
Nucleotide biosynthesis	Deoxycytidine kinase	NP_031858	363	8
	Ribonucleotide reductase M2	NP_033130	448	8
Pentose phophate shunt	Hexose-6-phosphate dehydrogenase (AI785303)	XP_181411	533	9
NAD(P) biosynthesis	Pre-B-cell colony-enhancing factor	NP_067499	327	9
Polyamine biosynthesis	Ornithine decarboxylase, structural	NP_038642	212	10
Tetrahydrobiopterin biosynthesis	GTP cyclohydrolase 1	NP_032128	259	10
Purin biosynthesis	Phosphoribosyl pyrophosphate amidotransferase	NP_742158	287	11
Asparagine biosynthesis	Asparagine synthetase	NP_036185	109	12
Long chain fatty acids	ELOVL family member 6, elongation of long chain fatty acids	NP_569717	162	12
Serine biosynthesis	Phosphoserine phosphatase	NP_598661	261	12
Gluconeogenesis	PEPCK 2 (Riken 9130022B02)	NP_083270	393	12
Prostaglandin E biosynthesis	Prostaglandin E synthase (ri|2410099E23; ri|9230102G02)	ri|2410099E23 ri|9230102G02	539 540	9

CoA, coenzyme A.

**Table 2:** Transcription factors that could regulate co-expressed genes in each cluster

Binding factors	Over-represented cluster	CS	FE	Putative target genes	Genes in cluster with promoter in PromoSer database	Putative target genes of all clusters
RORα1	Cluster 1	0.0322	0.0203	10	10	240
ATF	Cluster 2	0.0466	0.0481	15	27	133
CRE-BP1	Cluster 2	0.0050	0.0050	19	27	153
HLF	Cluster 2	0.0436	0.0452	15	27	132
XBP-1	Cluster 2	0.0378	0.0476	4	27	17
AhR	Cluster 2	0.0287	0.0446	3	27	9
Tal-1β/E47	Cluster 3	0.0400	0.0427	9	15	123
v-Maf	Cluster 4	0.0432	0.0308	2	12	11
SREBP-1	Cluster 4	0.0494	0.0484	9	12	166
Tal-1β/ITF-2	Cluster 5	0.0145	0.0169	19	46	89
Pbx-1	Cluster 5	0.0323	0.0206	45	46	312
NRF-2	Cluster 5	0.0310	0.0252	41	46	270
Sox-5	Cluster 5	-	0.0490	40	46	268
VBP	Cluster 5	0.0345	0.0276	42	46	281
NF-κB (p65)	Cluster 6	0.0354	0.0333	13	17	182
CCAAT box	Cluster 6	0.0458	0.0287	17	17	288
AP-2	Cluster 6	0.0330	0.0268	15	17	226
E4BP4	Cluster 8	0.0230	0.0243	31	69	113
CCAAT	Cluster 8	0.0211	0.0304	5	69	7
VBP	Cluster 8	0.0242	0.196	62	69	281
GC box	Cluster 9	-	0.0450	44	48	289
RREB-1	Cluster 10	0.0388	0.0435	13	42	65
SRF	Cluster 10	0.0221	0.0255	16	42	81
GC box	Cluster 10	0.0450	0.0366	39	42	289
Poly A downstream element	Cluster 11	0.0335	0.0431	5	13	55
E2	Cluster 12	0.0459	-	14	47	65

Probabilities for over-representation (<0.05) of genes having a predicted transcription factor binding site relative to the total of all clusters. CS, one-sided χ^2 ^test; FE, one-sided Fisher's exact test.

**Table 3:** Significance of occurrence of predicted SREBP-1 binding sites in the promoters of co-expressed genes identified by clustering

SREBP-1	Putative target genes	Genes in cluster	Against total PromoSer database		Against total of all clusters	
			
			CS	FE	CS	FE
Cluster 1	4	10	0.5000	0.7051	0.5339	0.7644
Cluster 2	11	27	0.5448	0.6892	0.6475	0.7812
Cluster 3	5	16	0.7076	0.8575	0.7697	0.8987
Cluster 4	9	12	0.0290	0.0289	0.0494	0.0484
Cluster 5	16	45	0.7787	0.8570	0.8568	0.9171
Cluster 6	10	20	0.1553	0.1554	0.2278	0.2277
Cluster 7	5	10	0.5000	0.5677	0.5000	0.6423
Cluster 8	31	66	0.2837	0.2827	0.4719	0.4711
Cluster 9	12	42	0.9025	0.9454	0.9414	0.9708
Cluster 10	22	41	0.1635	0.1635	0.2881	0.2877
Cluster 11	8	15	0.3230	0.3204	0.4057	0.4041
Cluster 12	25	45	0.0398	0.0404	0.1014	0.1014
PromoSer	5,456	12,493				

Probabilities for over-representation (<0.05) of genes having a predicted SREBP-1 site relative to all unique regulated genes of PromoSer and to the total of all clusters. Cluster 4 is the only one with significantly increased occurrence of predicted SREBP-1 binding sites. CS, one-sided χ^2 ^test; FE, one-sided Fisher's exact test; SREBP, sterol-regulatory element binding protein.
