# Supplementary figures and images for: Molecular processes during fat cell development revealed by gene expression profiling and functional annotation
Source: Genome Biol. 2005 Dec 19;6(13):R108. doi: 10.1186/gb-2005-6-13-r108 (PMC1414107; doi:10.1186/gb-2005-6-13-r108)

486 genes

### Development (18.1%)

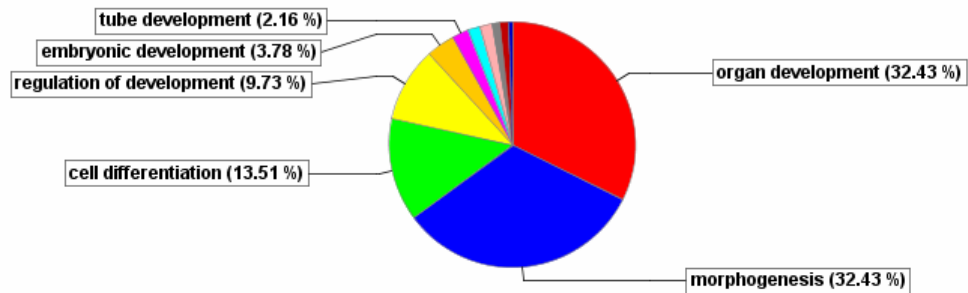

### Cellular processes (91.6%)

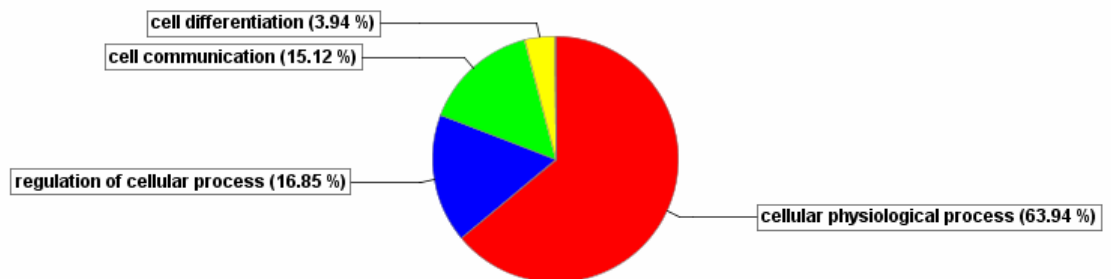

### Physiological processes (89.7%)

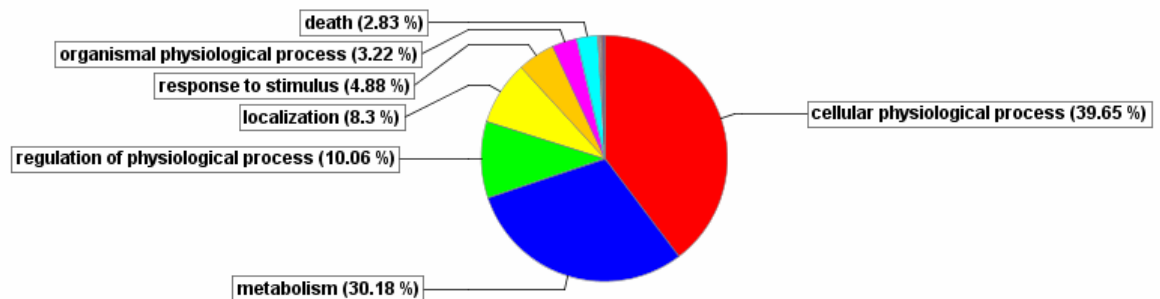

### Metabolism (286 genes)

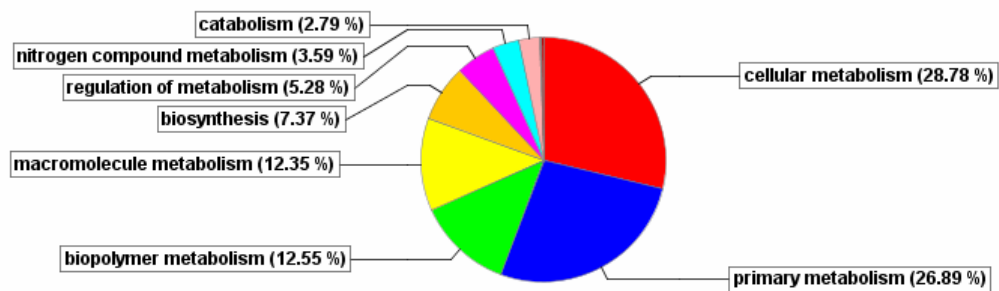

Supplement: Additional data file 6 — Images showing the distribution of gene ontology [file gb-2005-6-13-r108-S6.pdf]

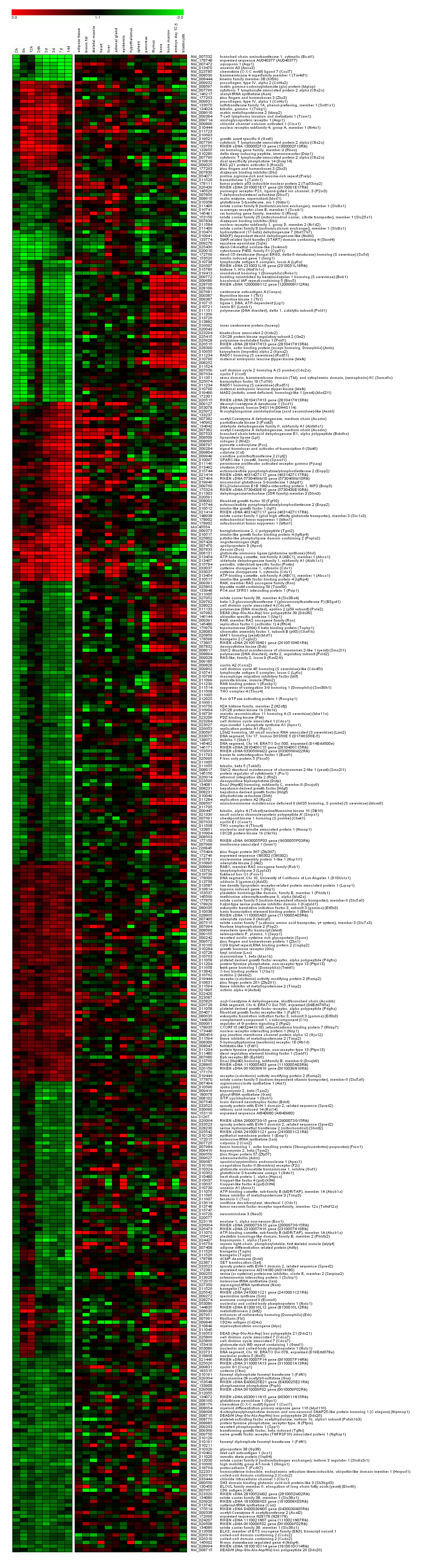

Supplement: Additional data file 11 — A figure showing a comparison with GeneAtlas [file gb-2005-6-13-r108-S11.png]

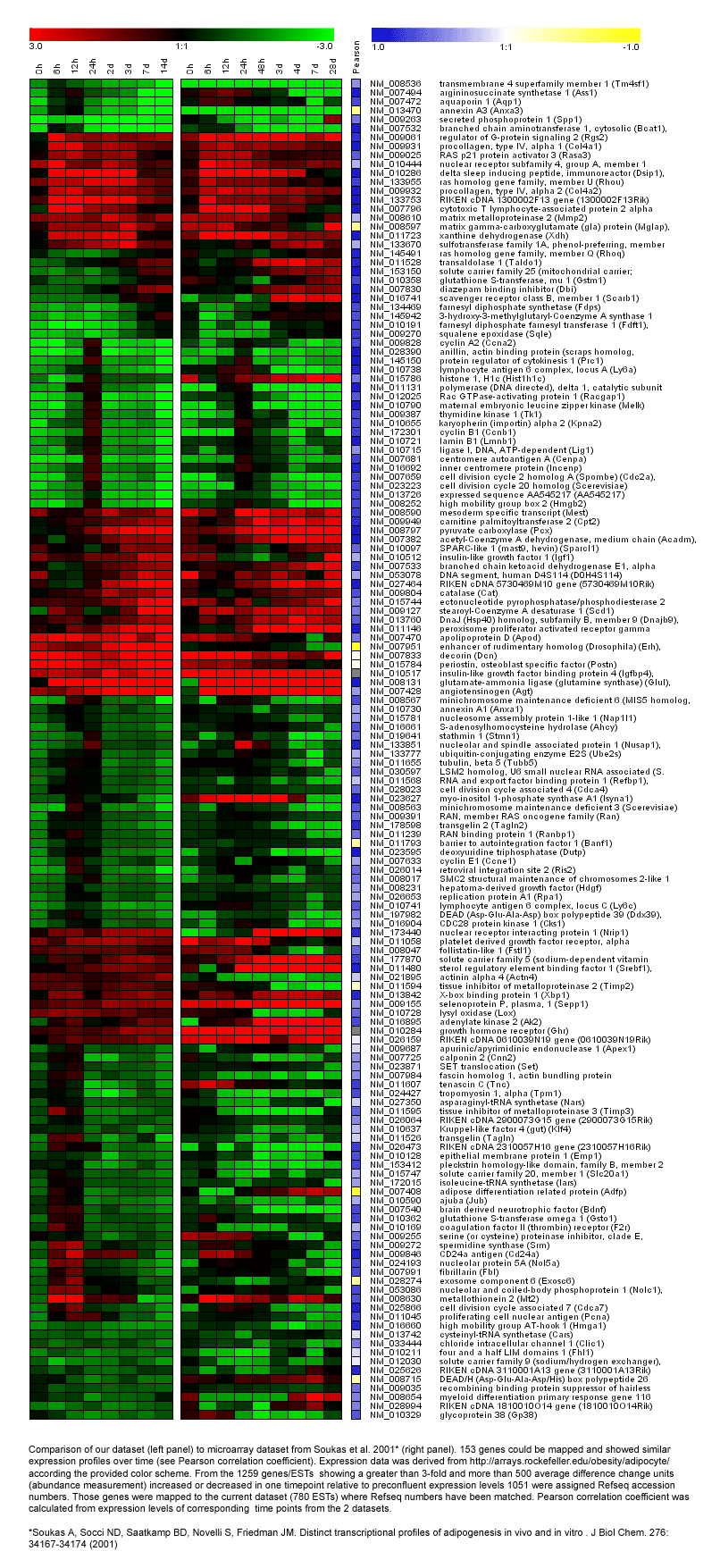

Supplement: Additional data file 13 — A figure showing a comparison with the data set reported by Soukas and coworkers [8] [file gb-2005-6-13-r108-S13.png]

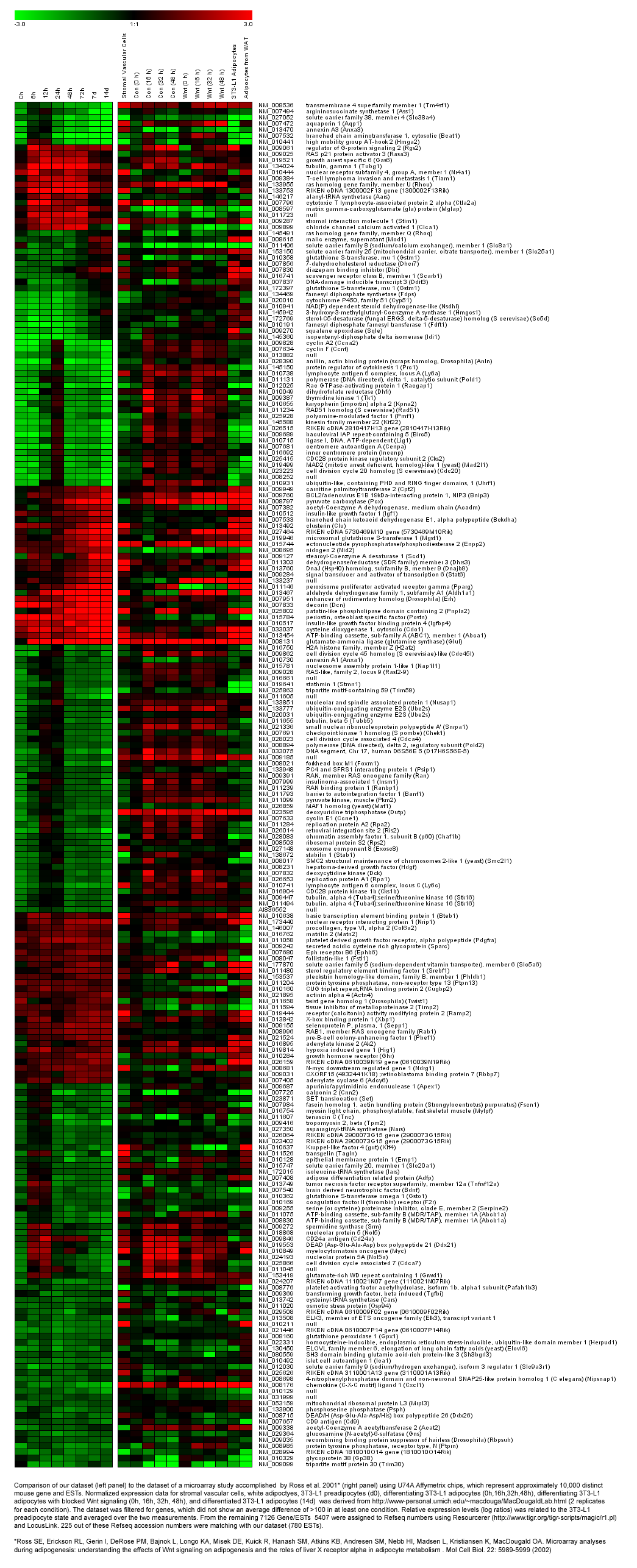

Supplement: Additional data file 14 — A figure showing a comparison with the data set reported by Ross and coworkers [9] [file gb-2005-6-13-r108-S14.png]

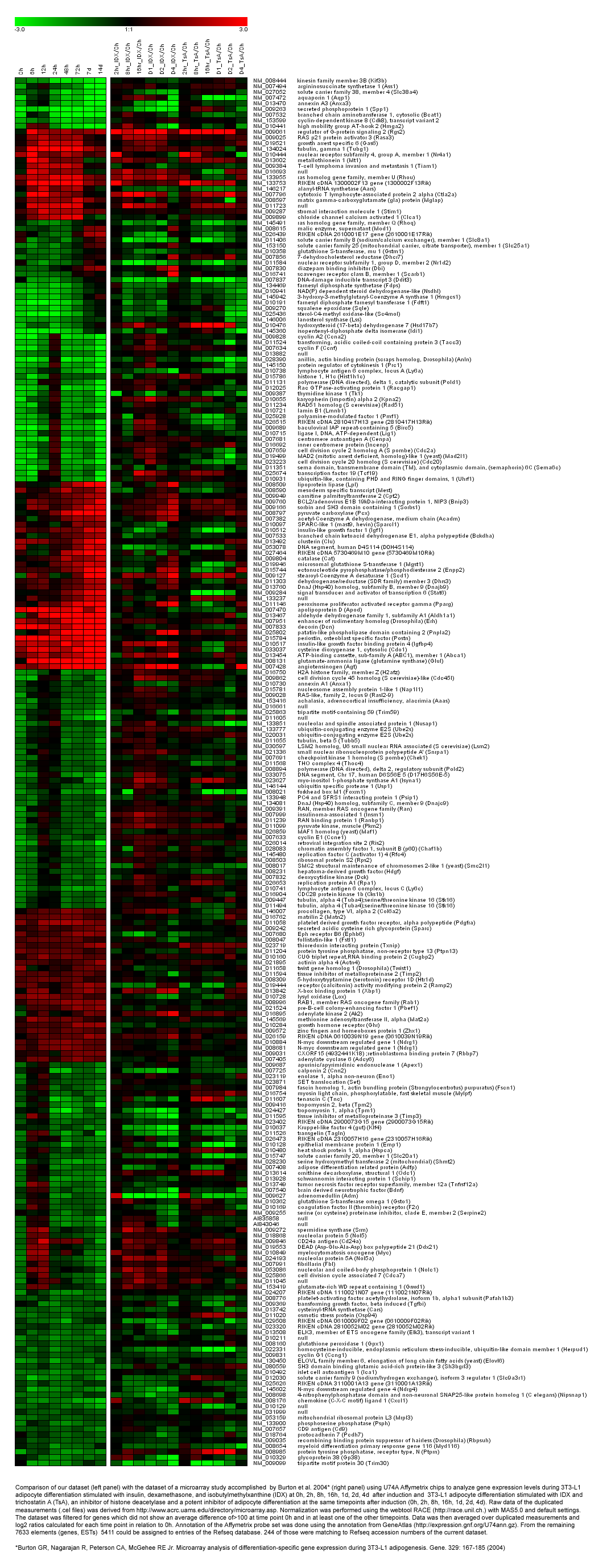

Supplement: Additional data file 15 — A figure showing a comparison with the data set reported by Burton and coworkers [12] [file gb-2005-6-13-r108-S15.png]

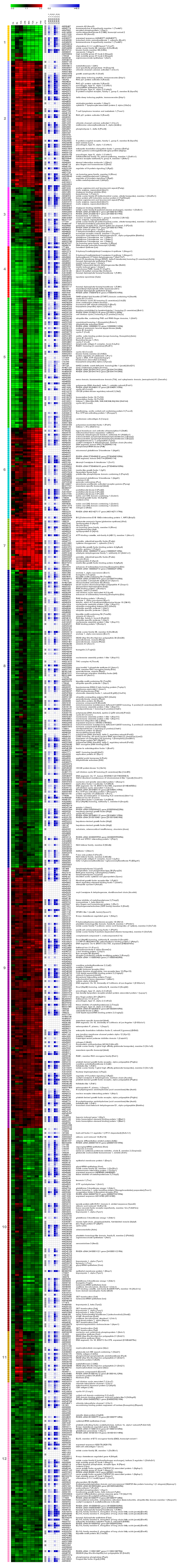

Supplement: Additional data file 17 — A figure showing genes with miRNA motifs in 3'-UTR [file gb-2005-6-13-r108-S17.png]

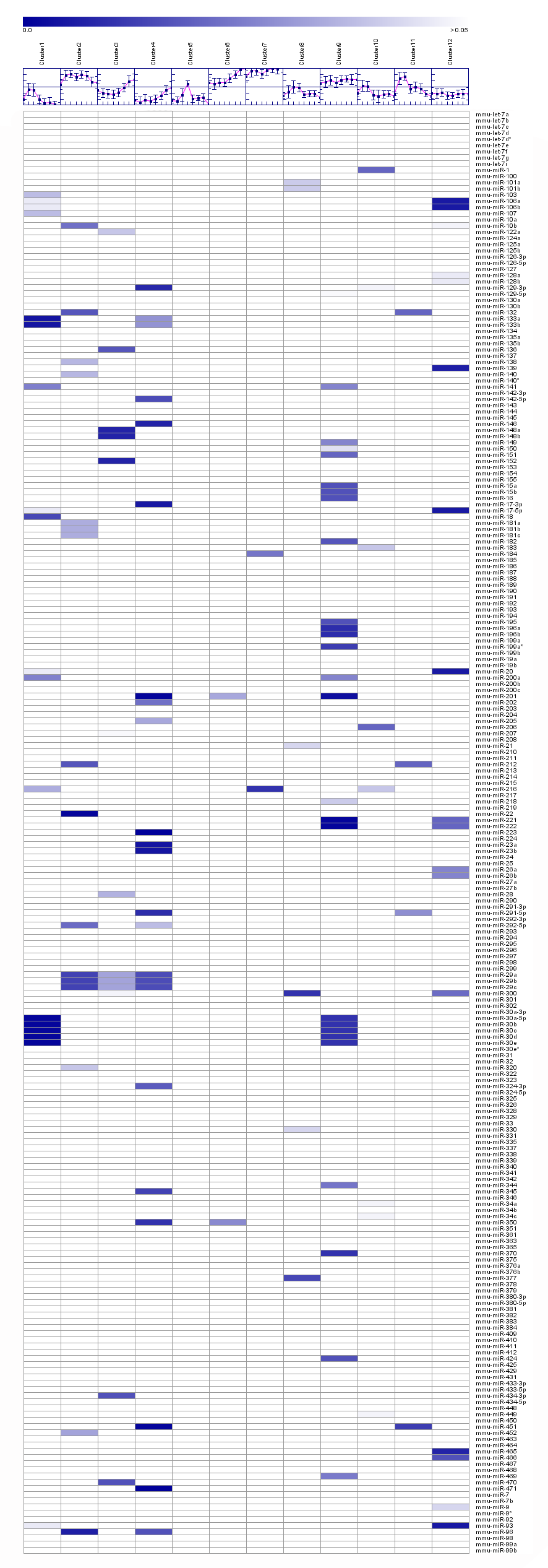

Supplement: Additional data file 18 — A figure illustrating the significant over-representation of miRNA motifs in the 3'-UTR of genes in each cluster [file gb-2005-6-13-r108-S18.png]

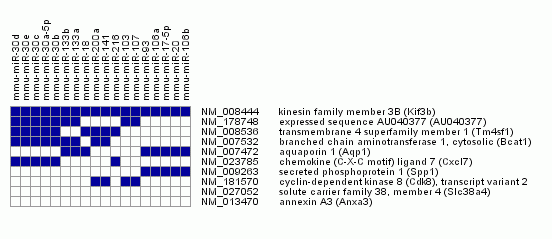

Supplement: Additional data file 19 — A figure showing the significant over-representation of miRNA motifs in the 3'-UTR from genes in cluster 1 [file gb-2005-6-13-r108-S19.png]

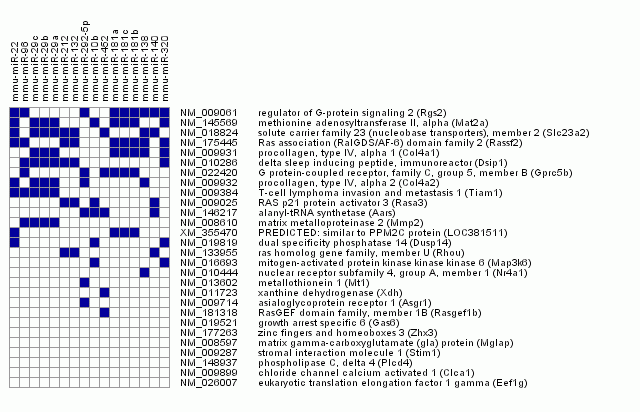

Supplement: Additional data file 20 — A figure showing the significant over-representation of miRNA motifs in the 3'-UTR from genes in cluster 2 [file gb-2005-6-13-r108-S20.png]

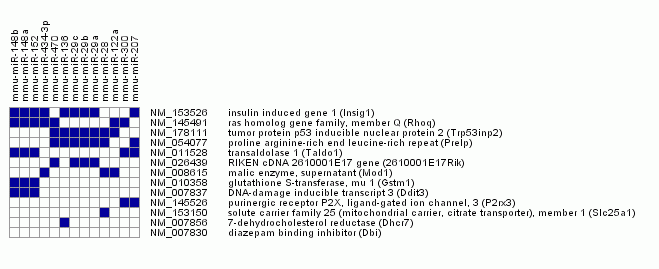

Supplement: Additional data file 21 — A figure showing the significant over-representation of miRNA motifs in the 3'-UTR from genes in cluster 3 [file gb-2005-6-13-r108-S21.png]

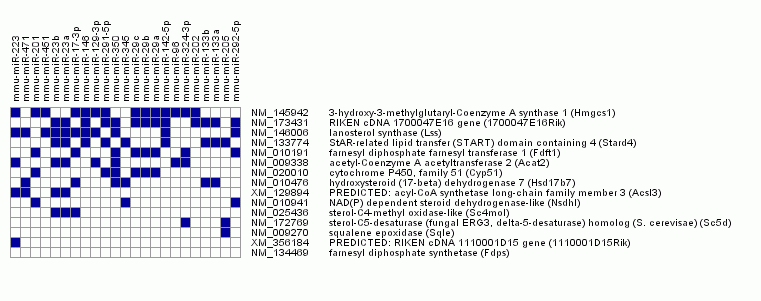

Supplement: Additional data file 22 — A figure showing the significant over-representation of miRNA motifs in the 3'-UTR from genes in cluster 4 [file gb-2005-6-13-r108-S22.png]

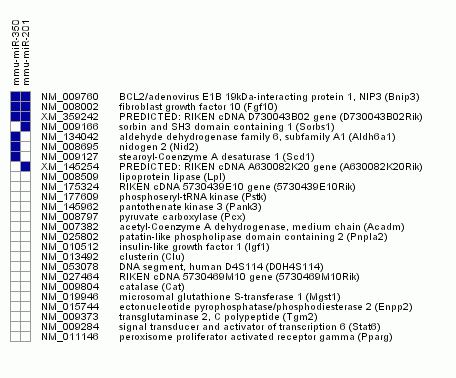

Supplement: Additional data file 23 — A figure showing the significant over-representation of miRNA motifs in the 3'-UTR from genes in cluster 6 [file gb-2005-6-13-r108-S23.png]

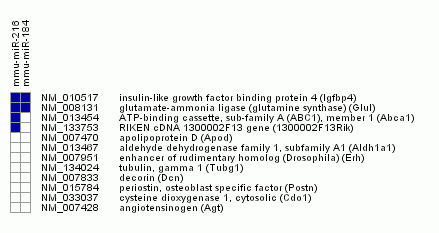

Supplement: Additional data file 24 — A figure showing the significant over-representation of miRNA motifs in the 3'-UTR from genes in cluster 7 [file gb-2005-6-13-r108-S24.png]

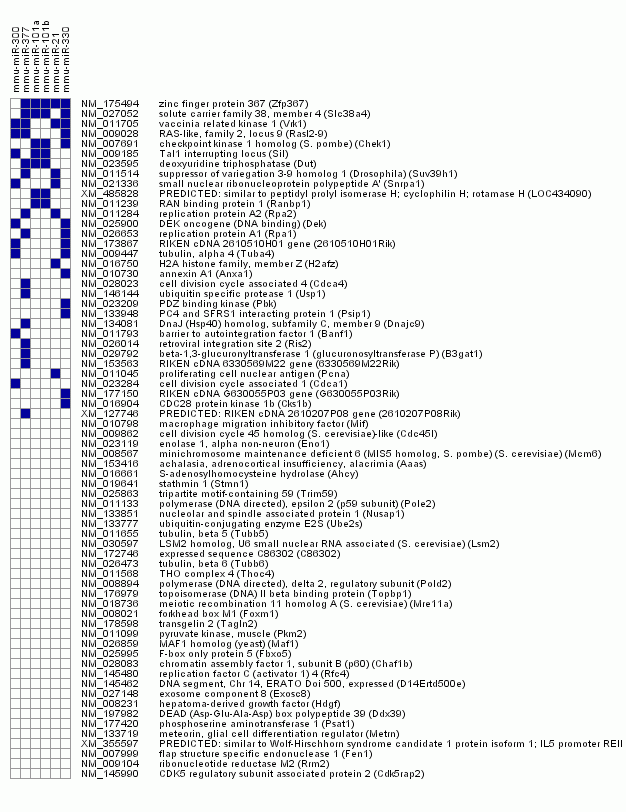

Supplement: Additional data file 25 — A figure showing the significant over-representation of miRNA motifs in the 3'-UTR from genes in cluster 8 [file gb-2005-6-13-r108-S25.png]

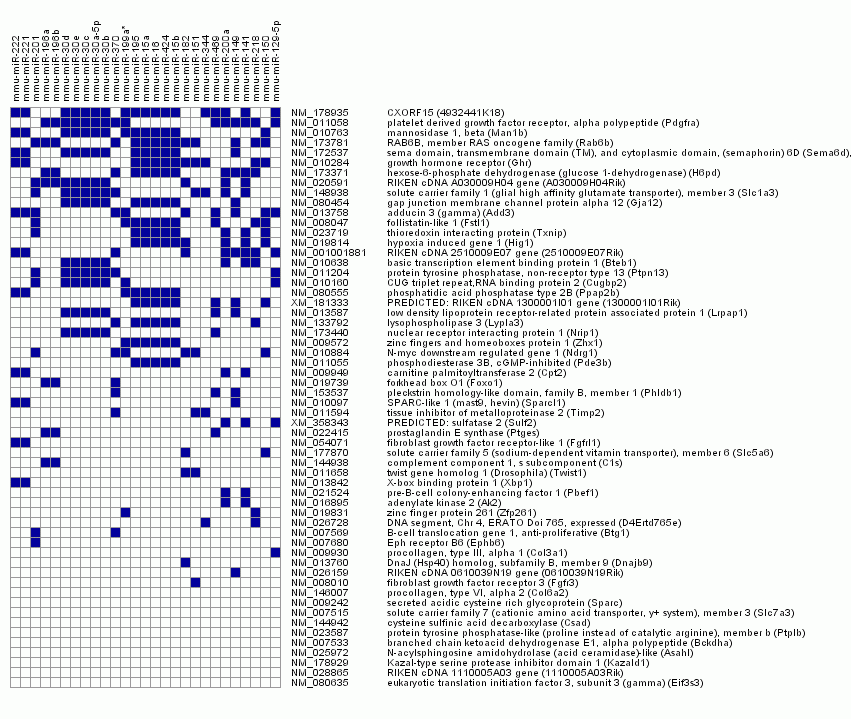

Supplement: Additional data file 26 — A figure showing the significant over-representation of miRNA motifs in the 3'-UTR from genes in cluster 9 [file gb-2005-6-13-r108-S26.png]

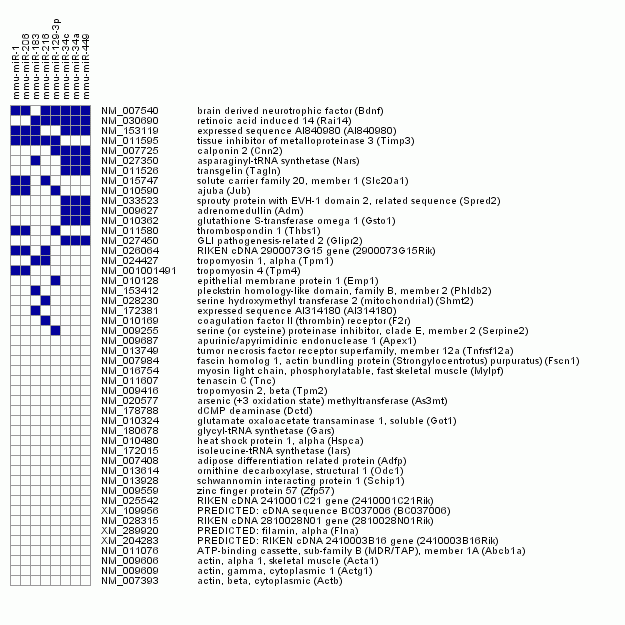

Supplement: Additional data file 27 — A figure showing the significant over-representation of miRNA motifs in the 3'-UTR from genes in cluster 10 [file gb-2005-6-13-r108-S27.png]

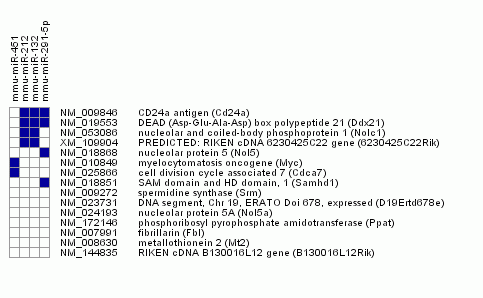

Supplement: Additional data file 28 — A figure showing the significant over-representation of miRNA motifs in the 3'-UTR from genes in cluster 11 [file gb-2005-6-13-r108-S28.png]

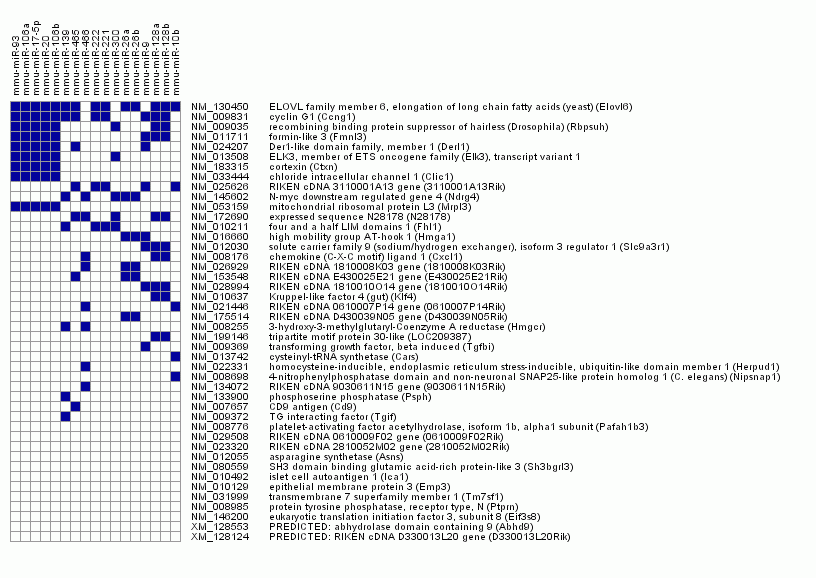

Supplement: Additional data file 29 — A figure showing the significant over-representation of miRNA motifs in the 3'-UTR from genes in cluster 12 [file gb-2005-6-13-r108-S29.png]

## 6h

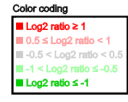

**12h**

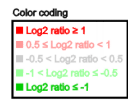

48h

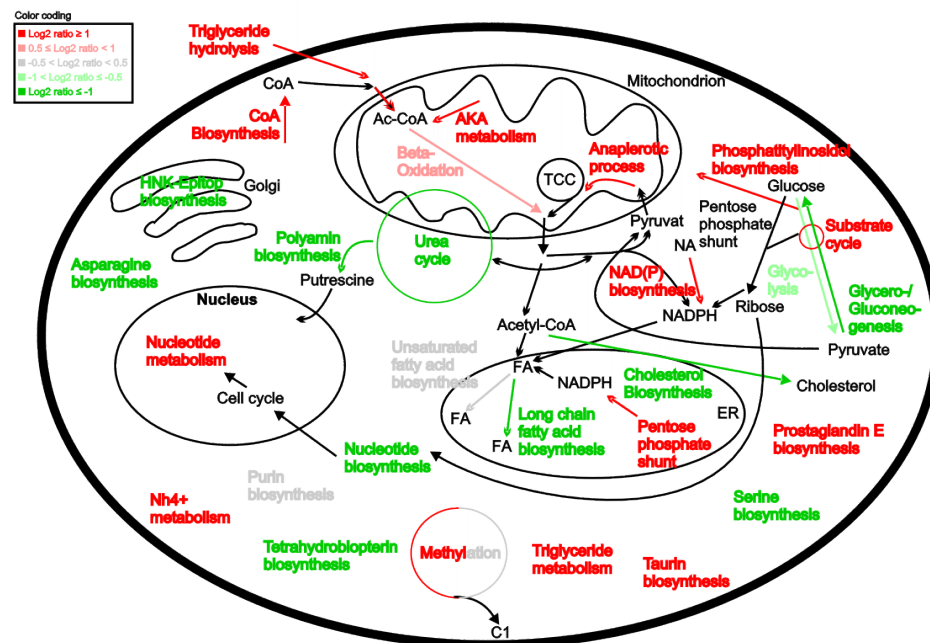

7d

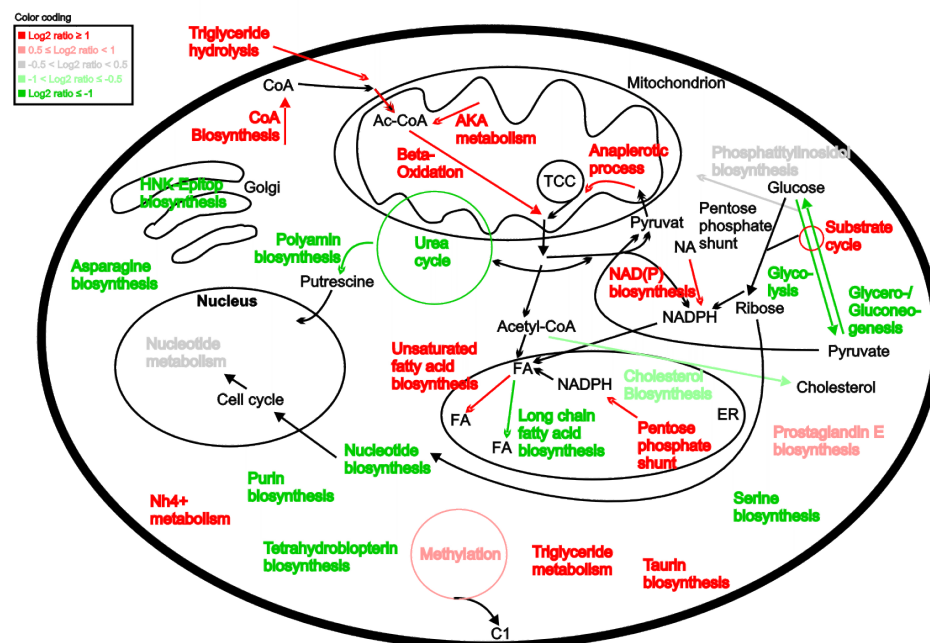

Supplement: Additional data file 32 — A figure showing regulation of metabolic pathways by key points [file gb-2005-6-13-r108-S32.pdf]

[illegible]

48h

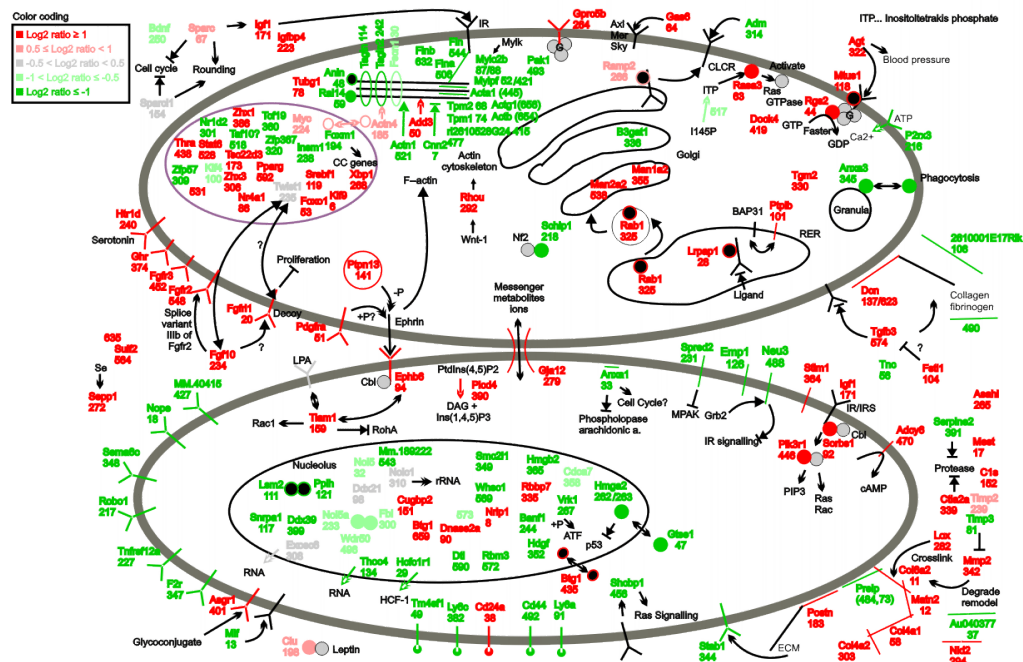

72h

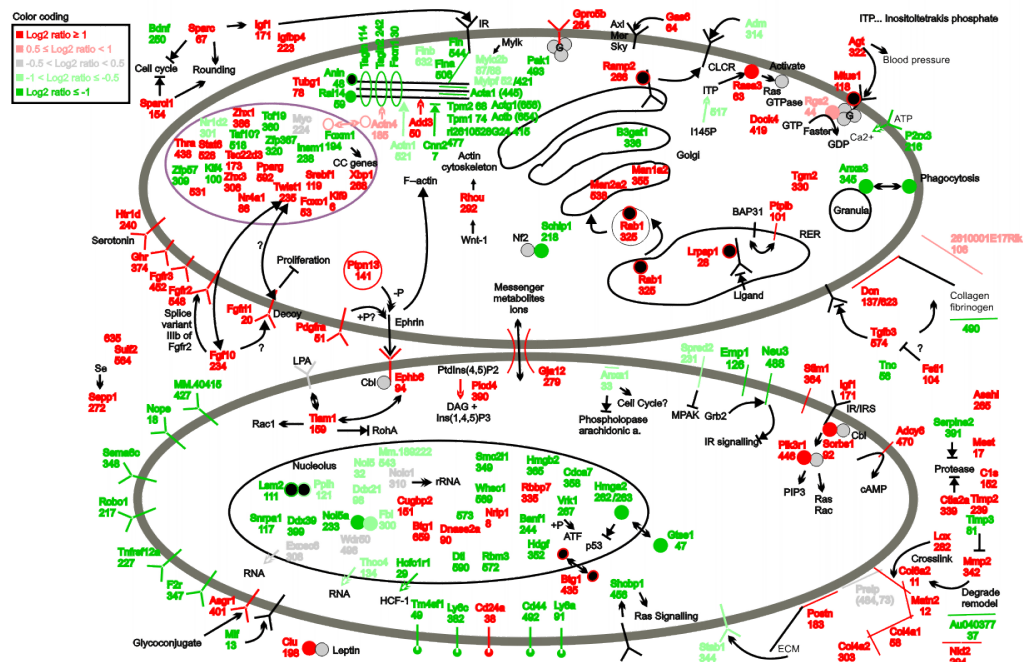

7d

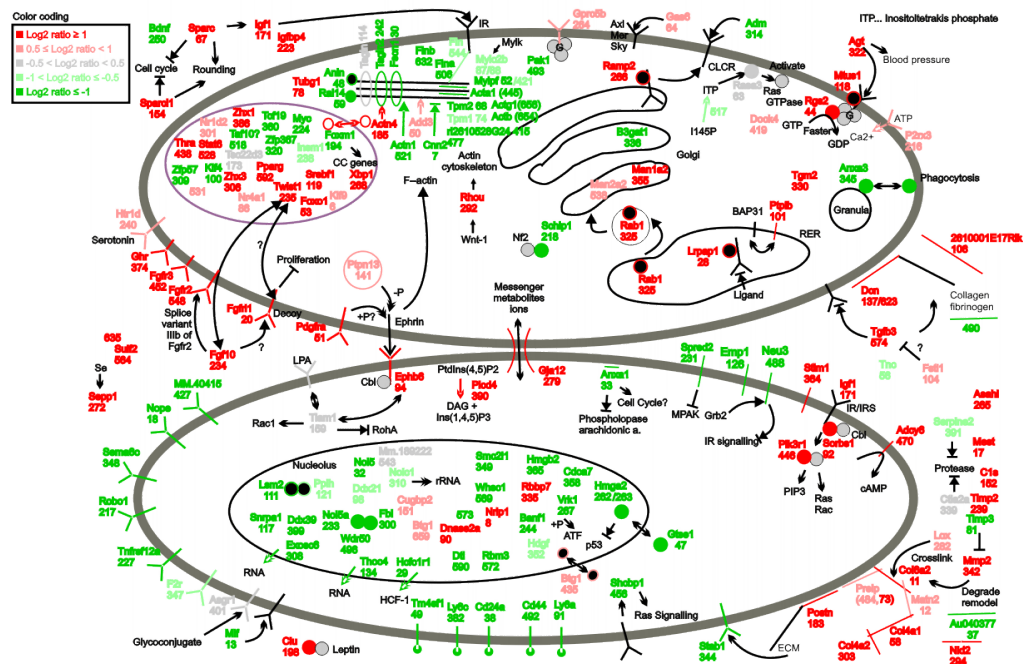

14d

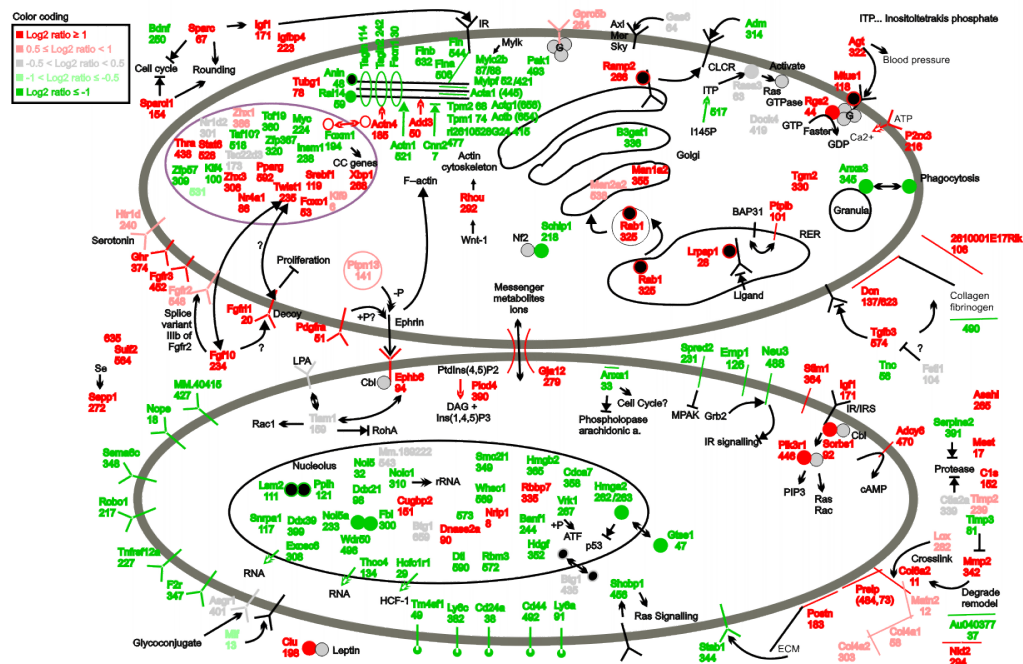

Supplement: Additional data file 34 — Figure showing the cellular localization of gene products involved in other biological processes and their gene expression at different time points [file gb-2005-6-13-r108-S34.pdf]

Cell cycle processes during adipocyte differentiation of 3T3-L1 cells

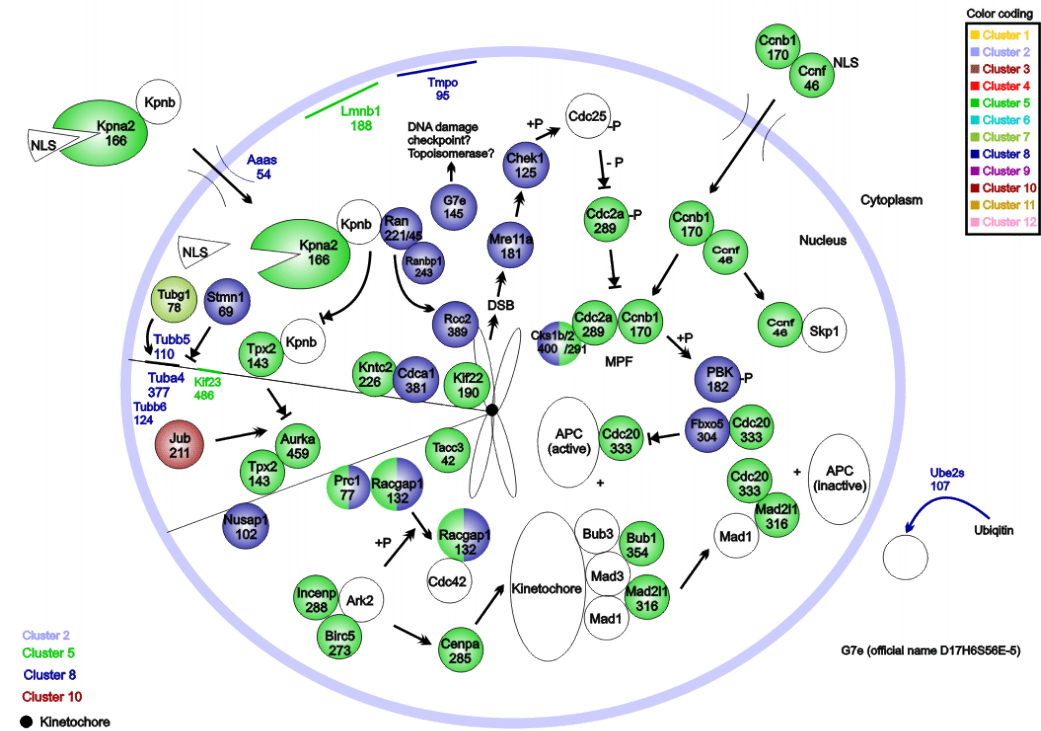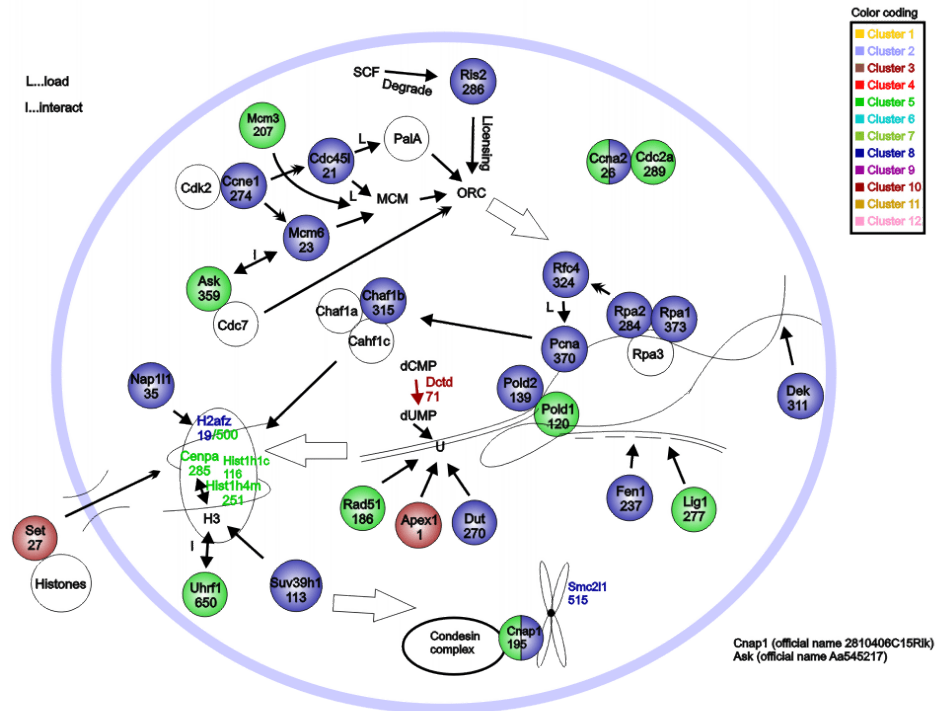

Supplement: Additional data file 37 — A figure showing cell cycle processes [file gb-2005-6-13-r108-S37.pdf]

## Cholesterol pathway during adipocyte differentiation of 3T3-L1 cells

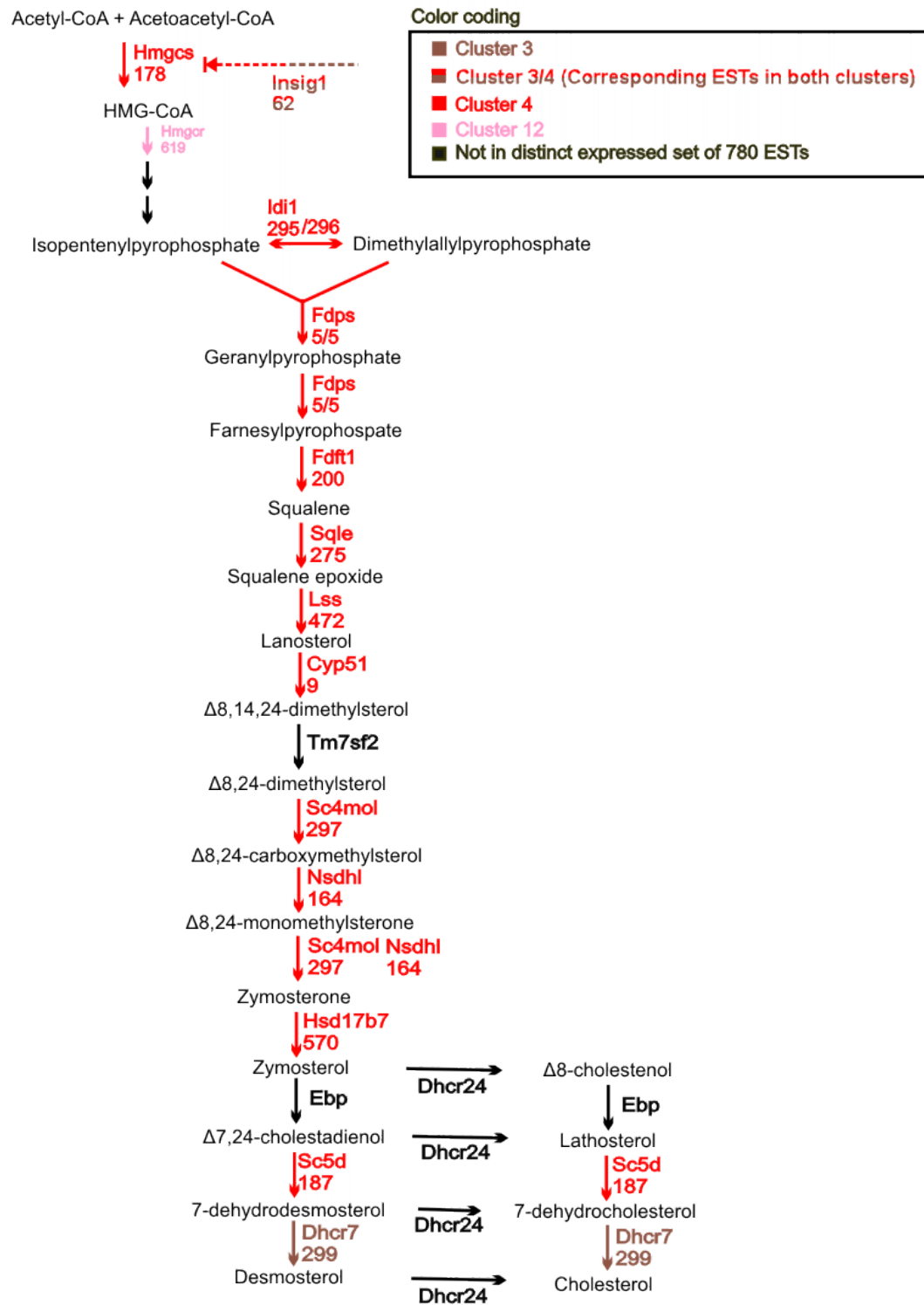

Supplement: Additional data file 38 — A figure showing the cholesterol pathway [file gb-2005-6-13-r108-S38.pdf]
