# Supplementary material for: Molecular processes during fat cell development revealed by gene expression profiling and functional annotation
Source: Genome Biol. 2005 Dec 19;6(13):R108. doi: 10.1186/gb-2005-6-13-r108 (PMC1414107; doi:10.1186/gb-2005-6-13-r108)

Sequences of forward an reverse LUX^{TM} primer for Real Time RT-PCR analysis to confirm microarray experiments for the given genes. Lower case letters (bases) on the 5'-end indicate complement to the 3'-end building a hairpin structure. Lower case t on the 3'-end indicates where the fluorophore is coupled.

Gene	RefSeq	Forward primer /Reverse primer
PPARg2	NM_011146	caccaTGCGGAAGCCCTTTGGtG<br>GGGCGGTCTCCACTGAGAAT
LPL	NM_008509	AGCAGACGCGGGAAGAGATT<br>caaccaAGGTCTTGCTGCTGTGGTtG
c-myc	NM_010849	CCCTAGTGCTGCATGAGGAGA<br>cagcgTTGCTCTTCTTCAGAGTCGCtG
Cyclin A2	NM_009828	CAGAGCTGGCCTGAGTCATTG<br>gacctaGTGGCGCTTTGAGGTAGGtC
Decorin	NM_007833	TCATAGAACTGGGCGGCAAC<br>caccaaAGGGATCGCAGTTATGTTGGtG
BTEB1	NM_010638	TGGCTGTGGGAAAGTCTATGG<br>atacagAAGGGCCGTTCACCTGTAtG

Comparison of RT-PCR results and microarray results from the second experiment and averaged over the 3 microarray experiments (biological replicates).

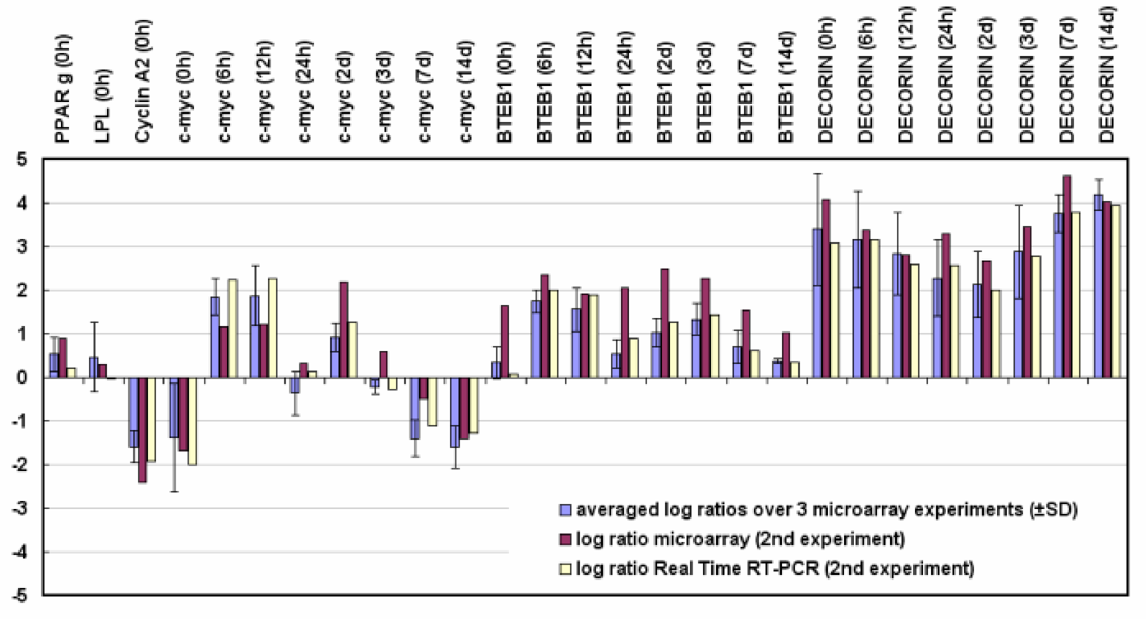

Supplement: Additional data file 9 — A pdf file containing real-time RT-PCR data [file gb-2005-6-13-r108-S9.pdf]
