# Supplementary material for: Molecular processes during fat cell development revealed by gene expression profiling and functional annotation
Source: Genome Biol. 2005 Dec 19;6(13):R108. doi: 10.1186/gb-2005-6-13-r108 (PMC1414107; doi:10.1186/gb-2005-6-13-r108)
Supplement: Additional data file 31 — Text describing regulation of metabolic pathways [file gb-2005-6-13-r108-S31.pdf]

Metabolic networks are regulated via key points of pathways at the transcriptional level

Among the 780 ESTs representing transcriptionally regulated genes, proteins encoded by them are members of 35 metabolic pathways. We studied whether these proteins occupy key positions in these pathways; i.e., a position at the pathway start, the typical rate-limiting step where the amount of enzyme is critical [1], or at a point of regulation. We found that transcriptionally regulated targets occupy such key positions in at least 26 pathways (overview in Table 1). The pathways, which are strongly transcriptionally regulated at the key points, are illustrated at the time points 0h, 24h, 48h and 14d in figure 4. Additional time points and images with more detailed information of all investigated pathways can be found at http://genome.tugraz.at/fatcell. In the following, we discuss adipocyte development-related functional aspects of several targets found with distinct expression profiles in this study.

The biosynthesis of the important lipogenic cofactors coenzyme A (CoA) and NAD(P)+ are transcriptionally regulated at their key enzymes. CoA is the carrier of the fatty acid precursor acetate/malonate [2,3]. Panthotenate kinase 3 (Pk3, No. 140, ~8-fold up-regulated) is responsible for the first and rate limiting step in converting panthotenate to CoA [4]. NADPH is necessary in reductive reactions for fatty acid synthesis. Pre-B-cell colony-enhancing factor (Visfatin/Pbef1, No. 327, up-regulated in the last three points of the time course in parallel to the emergence of fat droplets) is the rate limiting enzyme of NAD(P)+ biosynthesis [5,6]. For reduction of NADP+ to NADPH, two major mechanisms are responsible: the pentose phosphate shunt and the tricarboxylate transport system. Hexose-6P dehydrogenase (H6pd, No. 533, up-regulated during the whole time course) is the rate limiting enzyme of the pentose phosphate shunt in the endoplasmic reticulum and provides NADPH to its lumen [7]. In the cytosolic pendant in the pentose phosphate shunt, the transaldolase (No. 160) is repressed at early stages and ~3-fold up-regulated at the end of 3T3-L1 differentiation. This expression change appears to switch the shunt between ribose-5-phosphate (for nucleic acid synthesis) and NADPH (for fatty acid production) synthesis at early and late time points respectively. A similar expression profile is observed for the cytosolic NADP-dependent malic enzyme (Mod1, No. 76) and the citrate transporter (Ctp1, No. 209). Both are part of the tricarboxylate transport system through the mitochondrial membrane. Transcription of the anaplerotic pyruvate carboxylase (Pcx, No. 149, activated by acetyl-CoA) is increasingly up-regulated up to 16-fold towards the final two time points.

Fatty acid modification and assimilation is transcriptionally regulated at the rate limiting steps. The transcriptional expression of stearoyl-CoA desaturase (Scd, No. 305), which catalyzes the rate limiting reaction of monounsaturated fatty acid synthesis and which is an important marker gene of adipogenesis [8,9], is down-regulated at induction and it increases with advancing adipogenesis up to 60-fold after cell cycle. In contrast to literature, the gene of elongation of long chain fatty acid (LCE. No. 162) protein, which may be the rate limiting enzyme of long chain elongation to stearate [10] is not overexpressed in differentiated 3T3-L1 cells as in adipose tissue. LCE appears repressed during whole adipogenesis in 3T3-L1 cells. Possibly, another enzyme substitutes its function. Expression of lipoprotein lipase (LPL, No. 14), the rate limiting enzyme of extracellular triglyceride-rich lipoprotein hydrolyzation and triglyceride assimilation [11-13], increases with time up to 21-fold in differentiated adipocytes.

Transcriptional regulation of triglyceride and fatty acid degradation is performed at the key points. Adipose triglyceride lipase (ATGL, No. 157) executes the initial step in triglyceride metabolism [14]. Its expression increases strongly with differentiation progression. Acyl-coenzyme A dehydrogenases (ACAD, No. 153, 220) [15], the rate-limiting enzymes of medium, short and branched chain beta-oxidation, are strongly up-regulated in the final four time points. In contrast, the acyl-coenzyme A dehydrogenase of very long chain fatty acids is not in the set of distinctly differentially regulated genes and shows some up-regulation in the final two time points. This expression difference might shift the enrichment from short and medium to long chain fatty acids during adipogensis. Branched chain ketoacid dehydrogenase E1 (Bckdha, No. 193) is the rate limiting enzyme of leucine, valine and isoleucine katabolism and known to be inhibited by phosphorylation [16]. Its gene shares a similar expression profile with the ACAD genes. The elevated degradation of amino acids allows conversion to fatty acids through acetyl-CoA.

Several important nucleotide biosynthetic pathway enzymes follow a cell cycle-specific expression profile (strongly repressed except between 12h and 24h): Phosphoribosylpyrophosphate amidotransferse (Ppat, No. 287) [17] is rate limiting for purin production. Deoxycytidine kinase (Dck, No. 363) is the rate limiting enzyme of dC, dG and dA phosphorylation[18-20]. Ribonucleotide reductase M2 (Rrm2, No. 448) converts ribonucleotides to desoxyribonucleotides [21,22]. Additionally, thymidine kinase 1 (Tk1, No. 165) and dihydrofolate reductase (Dhfr, No. 161) have important roles in dT and purin biosynthesis during cell cycle. Contrary, purin degradation is ~6-fold up-regulated between 6h-72h by the rate limiting xanthine dehydrogenase (Xdh, No. 381,)[23,24].

The genes of argininesuccinate synthase (Ass1, No. 128), ornithine decarboxlyase (Odc, No. 212) and GTP cyclohydrolase 1 (Gch, No. 259) *have a cell cycle specific expression profile (not repressed between 6h-12h).* Ass1 is the rate limiting step of arginine-citrulline and urea cycle which might provide Odc with ornithine [25,26]. Odc inhibition delays differentiation to adipocyte-like cells[27]. Odc is the rate limiting enzyme in polyamine biosynthesis [28] which are bound in the nucleus by the polyamine-modulated factor 1 (Pmf1, No. 189, cell cycle profile) and can thereby activate the transcription factor Nrf2 (required for cell growth) [29]. Gch is the rate limiting step of tetrahydrobiopterin biosynthesis (key cofactor of nitric oxide synthase and of hydroxylation reactions) [30].

Many isoforms of glycolytic and gluconeogenic key points are transcriptionally down-regulated in comparison to pre-confluent cells during adipogenesis of 3T3-L1 cells but not during clonal expansion. This is the case for the allosterically regulated glycolytic isoenzyme pyruvate kinase 3 (Pk3, No. 247) and its predecessor Enolase 1 (Eno1, No. 22). Possibly, other isoforms absent on the chip are expressed. The gluconeogenic/glycerogenic rate limiting counterpart of Pk3, the mitochondrial phosphoenol pyruvate carboxylase (PEPCK, No. 393, permanently down-regulated) might be substituted by the cytosolic isoform (distribution tissue and organism specific) [31]. For instance, the fructose bisphosphatase 2 (Fbp2, No. 175), the muscle specific isoform of the glyconeogenic substrate cycle enzyme [32], is up-regulated during whole adipogenesis.

The genes of glutamine synthase (Glul, No. 318, ~18-fold) and cysteine dioxygenase (Cdo, No. 271, up to 24-fold) are very strongly transcriptionally up-regulated. The first plays a central role in N-metabolism to avoid ammonia accumulation. The latter is the rate limiting step in sulfate production and plays a key role in taurine biosynthesis and cysteine catabolism [33]. Contrary, asparagine synthase (Asns, No. 109) and phosphoserine phosphatase (Psph, No. 261), the key steps in asparagine [34] and serine biosynthesis [35], are repressed during whole adipogenesis.

The cellular methylation potential is transcriptionally regulated by S-adenosyl homocysteine hydrolase (Ahcy, No. 66, cell cycle profile) and methionine adenosyltransferase II, α (Mat2a, No. 350, up-regulated between 6h and 72h). Adenosylhomocysteine is a competitive inhibitor of the methyl transferase reaction and, therefore, Ahcy controls methylation by the concentration of this component [36]. Mat2a is the catalytic subunit of a methylation key step [37].

Of the inositol pathway, Myo-inositol-1-phosphate synthase A1 (Isyna1, No. 156) and the regulatory subunit of PI-3-kinase (Pik3r1, No. 446) have a distinct transcription profile. Isyna1 (strongly repressed at 0h, 6h, 7d and 14d) catalyzes the first and rate limiting step of all inositol-containing compounds (including important messengers) [38,39]. Pik3r1 (~4-fold up-regulated), the regulatory subunit of phosphatitylinositol-3-kinase, provides a link to an insulin-stimulated increase in glucose uptake [40].

Cholesterol biosynthesis is regulated by expression of key steps and whole pathway segments. The synthesis of the early precursor molecule 3-Hydroxy-3-methylglutaryl coenzyme A (HMG-CoA), which might be also used for other metabolic pathways, is transcriptional controlled at the key enzymes HMG-CoA synthase (Hmgcs1, No. 178, repressed except in terminal stages) and HMG-CoA reductase (Hmgcr, No. 619, always repressed), which is the rate limiting enzyme of cholesterol and mevalonate pathway [41,42]. After the step of isopentenylpyrophosphate synthesis, cholesterol biosynthesis genes are co-expressed in cluster 4.

References

1. Klipp E, Heinrich R, Holzhutter HG: **Prediction of temporal gene expression. Metabolic opimization by re-distribution of enzyme activities.** *Eur J Biochem* 2002, **269:**5406-5413.

2. Lynen F: **Acetyl coenzyme A and the fatty acid cycle.** *Harvey Lect* 1952, **48:**210-244.

3. Ganguly J: **Studies on the mechanism of fatty acid synthesis. VII. Biosynthesis of fatty acids from malonyl CoA.** *Biochim Biophys Acta* 1960, **40:**110-118.

4. Song WJ, Jackowski S: **Kinetics and regulation of pantothenate kinase from Escherichia coli.** *J Biol Chem* 1994, **269:**27051-27058.

5. Revollo JR, Grimm AA, Imai S: **The NAD biosynthesis pathway mediated by nicotinamide phosphoribosyltransferase regulates Sir2 activity in mammalian cells.** *J Biol Chem* 2004, **279:**50754-50763.

6. Rongvaux A, Shea RJ, Mulks MH, Gigot D, Urbain J, Leo O, Andris F: **Pre-B-cell colony-enhancing factor, whose expression is up-regulated in activated lymphocytes, is a nicotinamide phosphoribosyltransferase, a cytosolic enzyme involved in NAD biosynthesis.** *Eur J Immunol* 2002, **32:**3225-3234.

7. Clarke JL, Mason PJ: **Murine hexose-6-phosphate dehydrogenase: a bifunctional enzyme with broad substrate specificity and 6-phosphogluconolactonase activity.** *Arch Biochem Biophys* 2003, **415:**229-234.

8. Enoch HG, Catala A, Strittmatter P: **Mechanism of rat liver microsomal stearyl-CoA desaturase. Studies of the substrate specificity, enzyme-substrate interactions, and the function of lipid.** *J Biol Chem* 1976, **251:**5095-5103.

9. Ntambi JM: **Regulation of stearoyl-CoA desaturase by polyunsaturated fatty acids and cholesterol.** *J Lipid Res* 1999, **40:**1549-1558.

10. Moon YA, Shah NA, Mohapatra S, Warrington JA, Horton JD: **Identification of a mammalian long chain fatty acyl elongase regulated by sterol regulatory element-binding proteins.** *J Biol Chem* 2001, **276:**45358-45366.

11. Nilsson-Ehle P: **Impaired regulation of adipose tissue lipoprotein lipase in obesity.** *Int J Obes* 1981, **5:**695-699.

12. Semenkovich CF, Wims M, Noe L, Etienne J, Chan L: **Insulin regulation of lipoprotein lipase activity in 3T3-L1 adipocytes is mediated at posttranscriptional and posttranslational levels.** *J Biol Chem* 1989, **264:**9030-9038.

13. Koike T, Liang J, Wang X, Ichikawa T, Shiomi M, Liu G, Sun H, Kitajima S, Morimoto M, Watanabe T et al.: **Overexpression of lipoprotein lipase in transgenic Watanabe heritable hyperlipidemic rabbits improves hyperlipidemia and obesity.** *J Biol Chem* 2004, **279:**7521-7529.

14. Zimmermann R, Strauss JG, Haemmerle G, Schoiswohl G, Birner-Gruenberger R, Riederer M, Lass A, Neuberger G, Eisenhaber F, Hermetter A et al.: **Fat mobilization in adipose tissue is promoted by adipose triglyceride lipase.** *Science* 2004, **306:**1383-1386.

15. Zhang J, Zhang W, Zou D, Chen G, Wan T, Zhang M, Cao X: **Cloning and functional characterization of ACAD-9, a novel member of human acyl-CoA dehydrogenase family.** *Biochem Biophys Res Commun* 2002, **297:**1033-1042.

16. Harris RA, Hawes JW, Popov KM, Zhao Y, Shimomura Y, Sato J, Jaskiewicz J, Hurley TD: **Studies on the regulation of the mitochondrial alpha-ketoacid dehydrogenase complexes and their kinases.** *Adv Enzyme Regul* 1997, **37:**271-293.

17. Clark DV, MacAfee N: **The purine biosynthesis enzyme PRAT detected in proenzyme and mature forms during development of Drosophila melanogaster.** *Insect Biochem Mol Biol* 2000, **30:**315-323.

18. Bohman C, Eriksson S: **Deoxycytidine kinase from human leukemic spleen: preparation and characteristics of homogeneous enzyme.** *Biochemistry* 1988, **27:**4258-4265.

19. Hatzis P, Al Madhoon AS, Jullig M, Petrakis TG, Eriksson S, Talianidis I: **The intracellular localization of deoxycytidine kinase.** *J Biol Chem* 1998, **273:**30239-30243.

20. Sabini E, Ort S, Monnerjahn C, Konrad M, Lavie A: **Structure of human dCK suggests strategies to improve anticancer and antiviral therapy.** *Nat Struct Biol* 2003, **10:**513-519.

21. Wright JA, Chan AK, Choy BK, Hurta RA, McClarty GA, Tagger AY: **Regulation and drug resistance mechanisms of mammalian ribonucleotide reductase, and the significance to DNA synthesis.** *Biochem Cell Biol* 1990, **68:**1364-1371.

22. Dong Z, Liu LH, Han B, Pincheira R, Zhang JT: **Role of eIF3 p170 in controlling synthesis of ribonucleotide reductase M2 and cell growth.** *Oncogene* 2004, **23:**3790-3801.

23. Xu P, Huecksteadt TP, Harrison R, Hoidal JR: **Molecular cloning, tissue expression of human xanthine dehydrogenase.** *Biochem Biophys Res Commun* 1994, **199:**998-1004.

24. Xu P, Huecksteadt TP, Hoidal JR: **Molecular cloning and characterization of the human xanthine dehydrogenase gene (XDH).** *Genomics* 1996, **34:**173-180.

25. Shambaugh GE, III: **Urea biosynthesis I. The urea cycle and relationships to the citric acid cycle.** *Am J Clin Nutr* 1977, **30:**2083-2087.

26. Lemke CT, Howell PL: **The 1.6 A crystal structure of E. coli argininosuccinate synthetase suggests a conformational change during catalysis.** *Structure (Camb)* 2001, **9:**1153-1164.

27. Lowkvist B, Oredsson SM, Holm I, Emanuelsson H, Heby O: **Inhibition of polyamine synthesis reduces the growth rate and delays the expression of differentiated phenotypes in primary cultures of embryonic mesoderm from chick.** *Cell Tissue Res* 1987, **249:**151-160.

28. Kahana C, Nathans D: **Nucleotide sequence of murine ornithine decarboxylase mRNA.** *Proc Natl Acad Sci U S A* 1985, **82:**1673-1677.

29. Wang Y, Devereux W, Woster PM, Casero RA, Jr.: **Cloning and characterization of the mouse polyamine-modulated factor-1 (mPMF-1) gene: an alternatively spliced homologue of the human transcription factor.** *Biochem J* 2001, **359:**387-392.

30. Maita N, Hatakeyama K, Okada K, Hakoshima T: **Structural basis of biopterin-induced inhibition of GTP cyclohydrolase I by GFRP, its feedback regulatory protein.** *J Biol Chem* 2004, **279:**51534-51540.

31. Tontonoz P, Hu E, Devine J, Beale EG, Spiegelman BM: **PPAR gamma 2 regulates adipose expression of the phosphoenolpyruvate carboxykinase gene.** *Mol Cell Biol* 1995, **15:**351-357.

32. Tillmann H, Stein S, Liehr T, Eschrich K: **Structure and chromosomal localization of the human and mouse muscle fructose-1,6-bisphosphatase genes.** *Gene* 2000, **247:**241-253.

33. Soerbo B, Ewetz L: **The enzymatic oxidation of cysteine to cysteinesulfinate in rat liver.** *Biochem Biophys Res Commun* 1965, **18:**359-363.

34. Horowitz B, Meister A: **Glutamine-dependent asparagine synthetase from leukemia cells. Chloride dependence, mechanism of action, and inhibition.** *J Biol Chem* 1972, **247:**6708-6719.

35. Snell K, Fell DA: **Metabolic control analysis of mammalian serine metabolism.** *Adv Enzyme Regul* 1990, **30:**13-32.

36. Liu S, Wolfe MS, Borchardt RT: **Rational approaches to the design of antiviral agents based on S-adenosyl-L-homocysteine hydrolase as a molecular target.** *Antiviral Res* 1992, **19:**247-265.

37. Halim AB, LeGros L, Chamberlin ME, Geller A, Kotb M: **Regulation of the human MAT2A gene encoding the catalytic alpha 2 subunit of methionine adenosyltransferase, MAT II: gene organization, promoter characterization, and identification of a site in the proximal promoter that is essential for its activity.** *J Biol Chem* 2001, **276:**9784-9791.

38. Jin X, Geiger JH: **Structures of NAD(+)- and NADH-bound 1-l-myo-inositol 1-phosphate synthase.** *Acta Crystallogr D Biol Crystallogr* 2003, **59:**1154-1164.

39. Majumder AL, Johnson MD, Henry SA: **1L-myo-inositol-1-phosphate synthase.** *Biochim Biophys Acta* 1997, **1348:**245-256.

40. Clarke JF, Young PW, Yonezawa K, Kasuga M, Holman GD: **Inhibition of the translocation of GLUT1 and GLUT4 in 3T3-L1 cells by the phosphatidylinositol 3-kinase inhibitor, wortmannin.** *Biochem J* 1994, **300 (Pt 3):**631-635.

41. Popplewell PY, Azhar S: **Effects of aging on cholesterol content and cholesterol-metabolizing enzymes in the rat adrenal gland.** *Endocrinology* 1987, **121:**64-73.

42. Sato R, Takano T: **Regulation of intracellular cholesterol metabolism.** *Cell Struct Funct* 1995, **20:**421-427.
