# Supplementary material for: Molecular processes during fat cell development revealed by gene expression profiling and functional annotation
Source: Genome Biol. 2005 Dec 19;6(13):R108. doi: 10.1186/gb-2005-6-13-r108 (PMC1414107; doi:10.1186/gb-2005-6-13-r108)
Supplement: Additional data file 33 — Figure showing the cellular localization of gene products involved in metabolism and their gene expression at different time points [file gb-2005-6-13-r108-S33.pdf]

0h

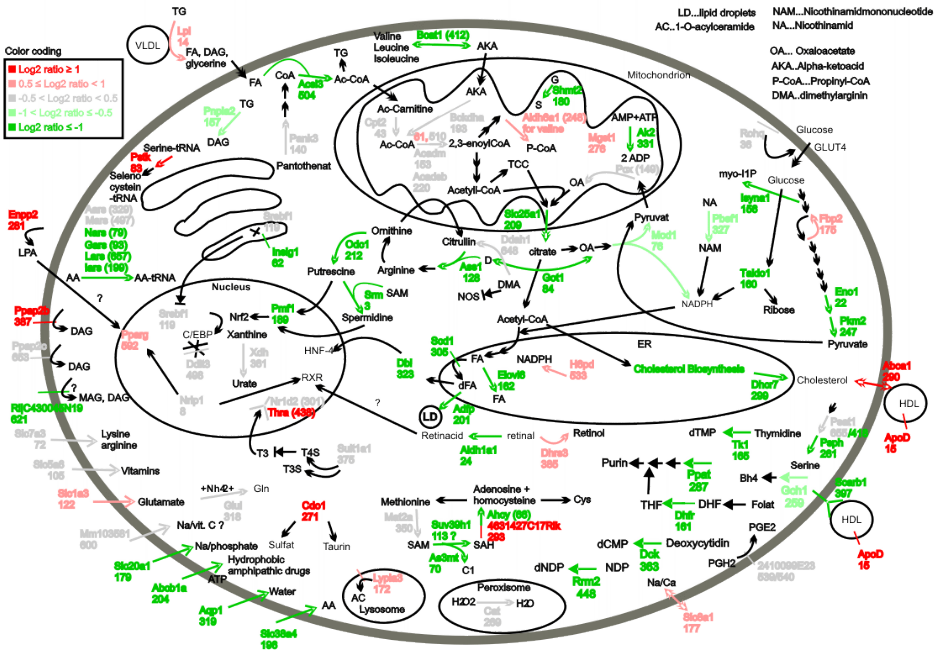

Color coding
Log2 ratio ≥ 1
0.5 ≤ Log2 ratio < 1
-0.5 < Log2 ratio < 0.5
-1 < Log2 ratio ≤ -0.5
Log2 ratio ≤ -1
LD...lipid droplets
AC..1-O-acylceramide
NAM...Nicothinamidmononucleotide
NA...Nicothinamid
OA... Oxaloacetate
AKA..Alpha-ketoacid
P-CoA...Propinyl-CoA
DMA..dimethylarginin
Mitochondrion
Nucleus
ER
Peroxisome
Lysosome

6h

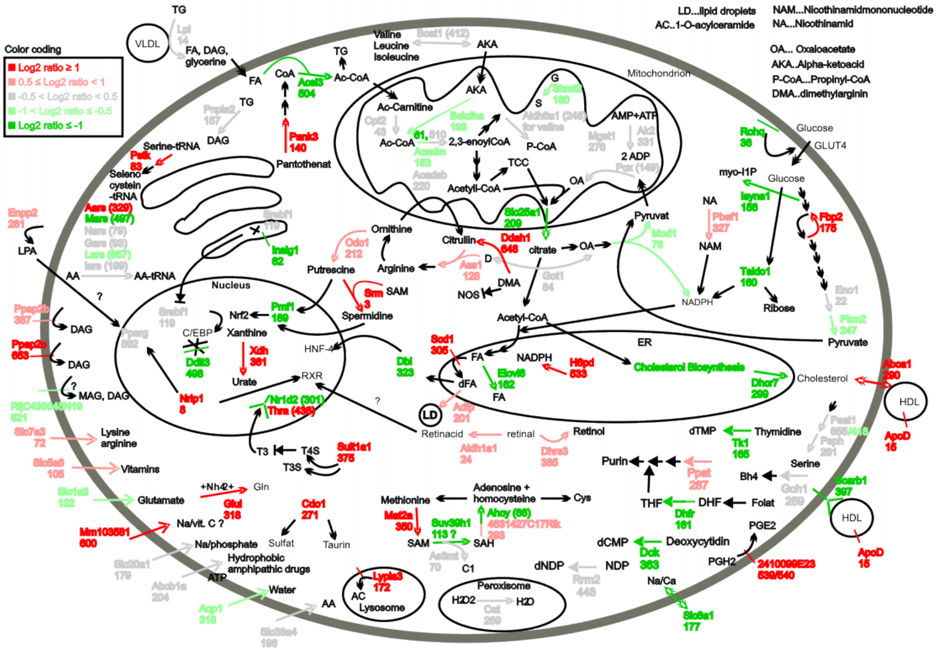

Color coding
Log2 ratio ≥ 1
0.5 ≤ Log2 ratio < 1
-0.5 < Log2 ratio < 0.5
-1 < Log2 ratio ≤ -0.5
Log2 ratio ≤ -1
LD...lipid droplets
AC..1-O-acylceramide
NAM...Nicothinamidmononucleotide
NA...Nicothinamid
OA... Oxaloacetate
AKA..Alpha-ketoacid
P-CoA...Propinyl-CoA
DMA..dimethylarginin
Mitochondrion
Nucleus
ER
Peroxisome
Lysosome

12h

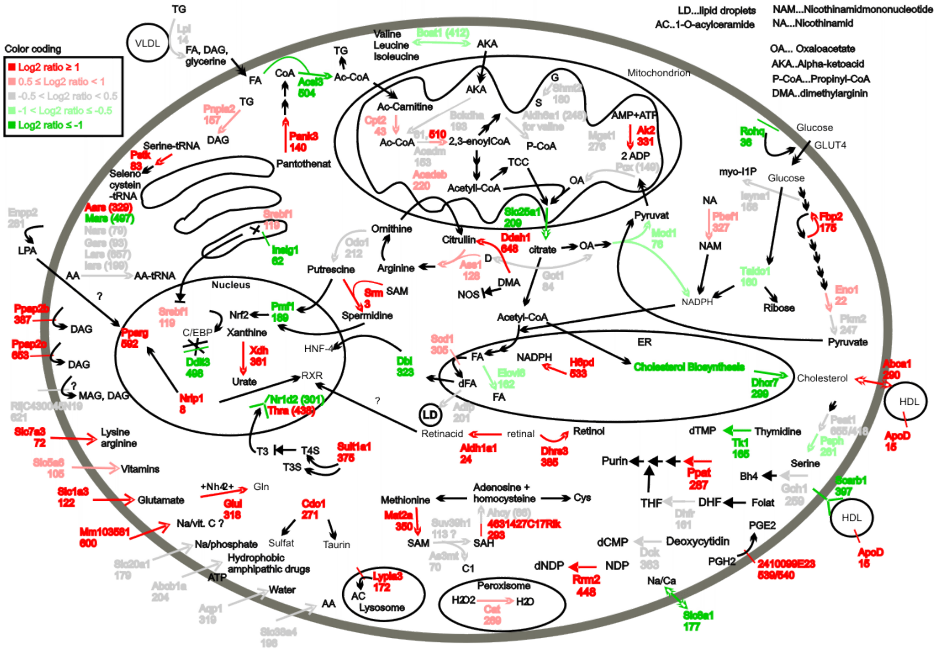
Color coding
Log2 ratio ≥ 1
0.5 ≤ Log2 ratio < 1
-0.5 < Log2 ratio < 0.5
-1 < Log2 ratio ≤ -0.5
Log2 ratio ≤ -1
LD...lipid droplets
AC..1-O-acylceramide
NAM...Nicothinamidmononucleotide
NA...Nicothinamid
OA... Oxaloacetate
AKA..Alpha-ketoacid
P-CoA...Propinyl-CoA
DMA..dimethylarginin

24h

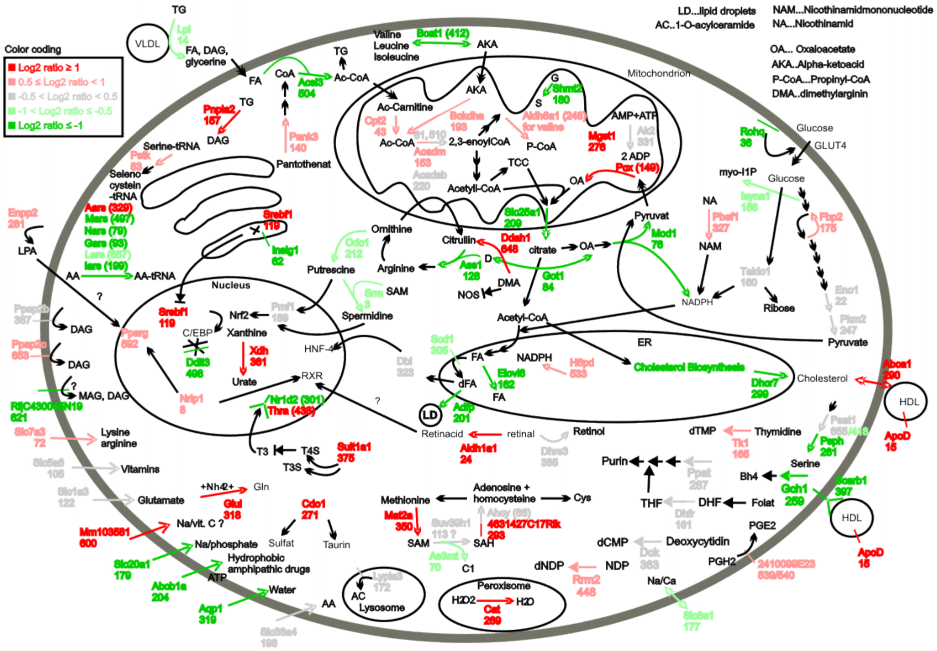
Color coding
Log2 ratio ≥ 1
0.5 ≤ Log2 ratio < 1
-0.5 < Log2 ratio < 0.5
-1 < Log2 ratio ≤ -0.5
Log2 ratio ≤ -1
LD...lipid droplets
AC..1-O-acylceramide
NAM...Nicothinamidmononucleotide
NA...Nicothinamid
OA... Oxaloacetate
AKA..Alpha-ketoacid
P-CoA...Propinyl-CoA
DMA..dimethylarginin

48h

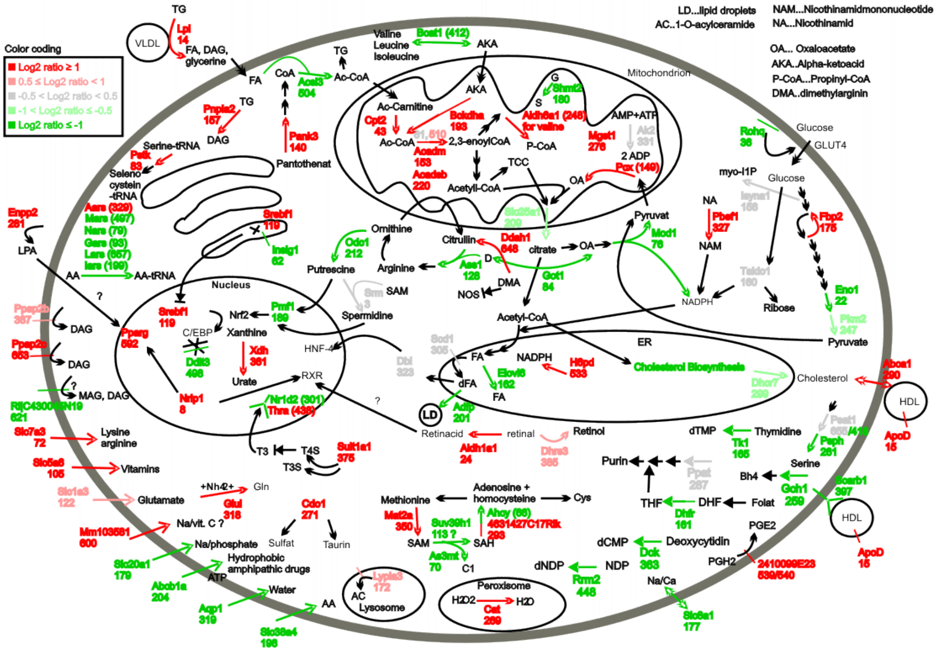

Color coding
Log2 ratio ≥ 1
0.5 ≤ Log2 ratio < 1
-0.5 < Log2 ratio < 0.5
-1 < Log2 ratio ≤ -0.5
Log2 ratio ≤ -1
LD...lipid droplets
AC..1-O-acylceramide
NAM...Nicothinamidmononucleotide
NA...Nicothinamid
OA... Oxaloacetate
AKA..Alpha-ketoacid
P-CoA...Propinyl-CoA
DMA..dimethylarginin
TG
Lpl 14
VLDL
FA, DAG, glycerine
FA
CoA
Acss3 504
Ac-CoA
Valine Leucine Isoleucine
Bcat1 (412)
AKA
Mitochondrion
Ac-Carnitine
Cpt2 43
Ac-CoA
Bckdha 193
2,3-enoylCoA
Acadm 153
Acadsb 220
Aldh6a1 (248) for valine
P-CoA
Shmt2 180
Mgst1 276
AMP+ATP
Ak2 331
2 ADP
Pcx (149)
TCC
Acetyl-CoA
OA
Pnpla2 157
DAG
Pank3 140
Pantothenat
Serine-tRNA
Pstk 83
Seleno cystein -tRNA
Aars (329)
Mars (487)
Nars (79)
Gars (93)
Lars (667)
Iars (199)
AA
AA-tRNA
Srebf1 119
Insig1 62
Enpp2 281
LPA
Ppap2b 357
DAG
Ppap2a 653
DAG
MAG, DAG
RIKEN C430002N19 621
Nucleus
Srebf1 119
Nrf2
Pparg 592
C/EBP
Ddit3 498
Xanthine
Xdh 361
Urate
Nrip1 8
RXR
HNF-4
Nr1d2 (301)
Thra (436)
Pmf1 169
Ornithine
Odc1 212
Putrescine
Arginine
Srm 3
SAM
Spermidine
Citrullin
Ddah1 848
Ass1 128
DMA
NOS
citrate
OA
Got1 84
Slc25a1 209
Pyruvat
Mod1 78
NADPH
NA
Pbef1 327
NAM
Acetyl-CoA
ER
Sod1 305
Dbi 323
FA
NADPH
H6pd 533
Cholesterol Biosynthesis
Dhcr7 299
Cholesterol
Elovl6 162
dFA
FA
LD
Adfp 201
Retinacid
Aldh1a1 24
retinal
Dhrs3 385
Retinol
T3
T4S
T3S
Sult1a1 375
Slc7a3 72
Lysine arginine
Slc5a6 105
Vitamins
Slc1a3 122
Glutamate
+Nh4 2+
Gln
Glul 318
Mm103681 600
Na/vit. C ?
Cdo1 271
Sulfat
Taurin
Slc20a1 179
Na/phosphate
Hydrophobic amphipathic drugs
Abcb1a 204
ATP
Aqp1 319
Water
Slc38a4 196
AA
AC
Lysosome
Lypla3 172
Peroxisome
H2O2
H2O
Cat 269
Methionine
Adenosine + homocysteine
Cys
Mat2a 380
Suv39h1 113 ?
SAM
Ahcy (66)
4631427C17Rik 293
SAH
As3mt 70
C1
dCMP
Dck 363
Deoxycytidin
dNDP
Rrm2 448
NDP
Na/Ca
Slc8a1 177
PGE2
PGH2
2410099E23 539/540
Rohc 38
Glucose
GLUT4
myo-I1P
Isyna1 166
Glucose
Fbp2 175
Taldo1 180
Ribose
Eno1 22
Pkm2 247
Pyruvate
Abca1 290
HDL
ApoD 15
dTMP
Tk1 166
Thymidine
Psat1 /412
Pgph 281
Serine
Purin
Ppat 287
Bh4
Gch1 259
Scarb1 397
THF
DHF
Dhfr 161
Folat
HDL
ApoD 15

72h

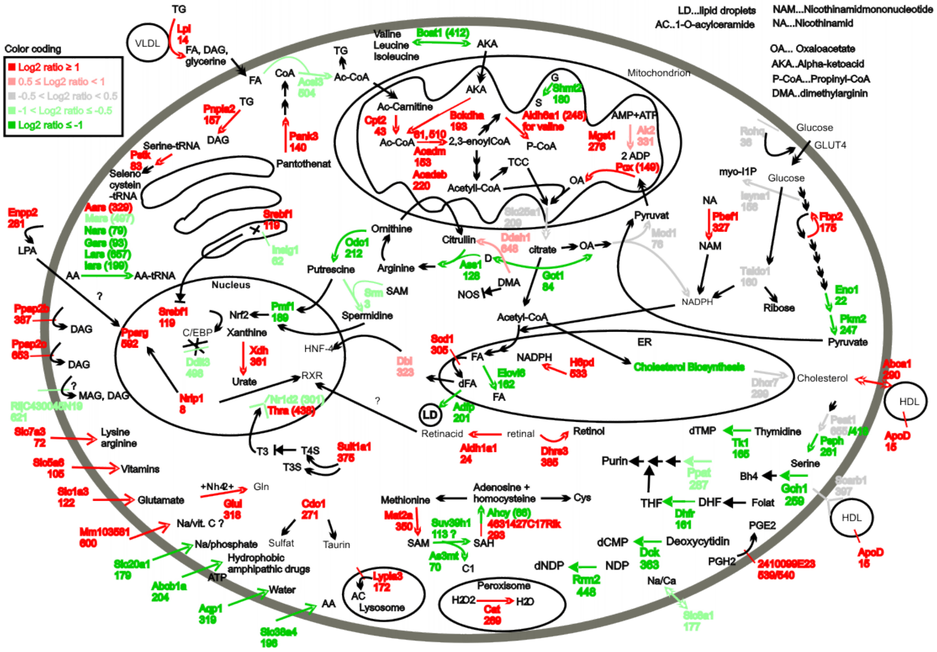

Color coding
Log2 ratio ≥ 1
0.5 ≤ Log2 ratio < 1
-0.5 < Log2 ratio < 0.5
-1 < Log2 ratio ≤ -0.5
Log2 ratio ≤ -1
LD...lipid droplets
AC..1-O-acylceramide
NAM...Nicothinamidmononucleotide
NA...Nicothinamid
OA... Oxaloacetate
AKA..Alpha-ketoacid
P-CoA...Propinyl-CoA
DMA..dimethylarginin
TG
Lpl 14
VLDL
FA, DAG, glycerine
FA
CoA
Acss3 504
Ac-CoA
Valine Leucine Isoleucine
Bcat1 (412)
AKA
Mitochondrion
Ac-Carnitine
Cpt2 43
Ac-CoA
61,510
Bckdha 193
2,3-enoylCoA
Acadm 153
Acadsb 220
Aldh6a1 (248) for valine
P-CoA
Shmt2 180
Mgst1 276
AMP+ATP
Ak2 331
2 ADP
Pcx (149)
TCC
Acetyl-CoA
OA
Pnpla2 157
DAG
Pank3 140
Pantothenat
Serine-tRNA
Pstk 83
Seleno cystein -tRNA
Aars (329)
Mars (487)
Nars (79)
Gars (93)
Lars (667)
Iars (199)
AA
AA-tRNA
Srebf1 119
Insig1 62
Enpp2 281
LPA
Ppap2b 357
DAG
Ppap2a 653
DAG
MAG, DAG
RIKEN C430002N19 621
Nucleus
Srebf1 119
Nrf2
Pparg 592
C/EBP
Ddit3 498
Xanthine
Xdh 361
Urate
Nrip1 8
RXR
HNF-4
Nr1d2 (301)
Thra (436)
Pmf1 169
Ornithine
Odc1 212
Putrescine
Arginine
Srm 3
SAM
Spermidine
Citrullin
Ddah1 848
Ass1 128
DMA
NOS
citrate
OA
Got1 84
Slc25a1 209
Pyruvat
Mod1 78
NADPH
NA
Pbef1 327
NAM
Acetyl-CoA
ER
Sod1 305
Dbi 323
FA
NADPH
H6pd 533
Cholesterol Biosynthesis
Dhcr7 299
Cholesterol
Elovl6 162
dFA
FA
LD
Adfp 201
Retinacid
Aldh1a1 24
retinal
Dhrs3 385
Retinol
T3
T4S
T3S
Sult1a1 375
Slc7a3 72
Lysine arginine
Slc5a6 105
Vitamins
Slc1a3 122
Glutamate
+Nh4 2+
Gln
Glul 318
Mm103681 600
Na/vit. C ?
Cdo1 271
Sulfat
Taurin
Slc20a1 179
Na/phosphate
Hydrophobic amphipathic drugs
Abcb1a 204
ATP
Aqp1 319
Water
Slc38a4 196
AA
AC
Lysosome
Lypla3 172
Peroxisome
H2O2
H2O
Cat 269
Methionine
Adenosine + homocysteine
Cys
Mat2a 380
Suv39h1 113 ?
SAM
Ahcy (66)
4631427C17Rik 293
SAH
As3mt 70
C1
dCMP
Dck 363
Deoxycytidin
dNDP
Rrm2 448
NDP
Na/Ca
Slc8a1 177
PGE2
PGH2
2410099E23 539/540
Rohc 38
Glucose
GLUT4
myo-I1P
Isyna1 166
Glucose
Fbp2 175
Taldo1 180
Ribose
Eno1 22
Pkm2 247
Pyruvate
Abca1 290
HDL
ApoD 15
dTMP
Tk1 166
Thymidine
Psat1 /412
Pgph 281
Serine
Purin
Ppat 287
Bh4
Gch1 259
Scarb1 397
THF
DHF
Dhfr 161
Folat
HDL
ApoD 15

7d

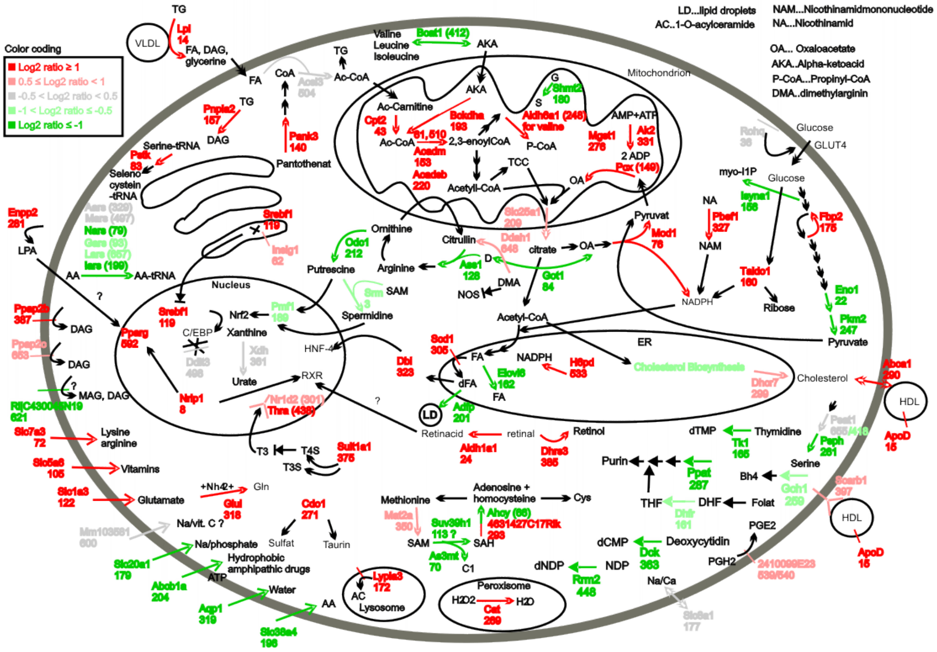
Color coding
Log2 ratio ≥ 1
0.5 ≤ Log2 ratio < 1
-0.5 < Log2 ratio < 0.5
-1 < Log2 ratio ≤ -0.5
Log2 ratio ≤ -1
LD...lipid droplets
AC..1-O-acylceramide
NAM...Nicothinamidmononucleotide
NA...Nicothinamid
OA... Oxaloacetate
AKA..Alpha-ketoacid
P-CoA...Propinyl-CoA
DMA..dimethylarginin
TG
Lpl 14
VLDL
FA, DAG, glycerine
Valine Leucine Isoleucine
Bcat1 (412)
AKA
CoA
Acss3 804
Ac-CoA
Mitochondrion
Ac-Carnitine
Cpt2 43
81,810
Bckdha 193
2,3-enoylCoA
Aldh6a1 (248) for valine
P-CoA
Acadm 153
Acadsb 220
Acetyl-CoA
TCC
OA
Shmt2 180
AMP+ATP
Ak2 331
Mgst1 276
2 ADP
Pcx (149)
Rohc 38
Glucose
GLUT4
myo-I1P
Isyna1 158
Fbp2 175
Pnpla2 157
DAG
Pank3 140
Pantothenat
Serine-tRNA
Pstk 83
Seleno cystein -tRNA
Enpp2 281
LPA
Aars (329)
Mars (497)
Nars (79)
Gars (93)
Lars (857)
Iars (199)
AA
AA-tRNA
Srebf1 119
Insig1 62
Ornithine
Odc1 212
Citrullin
Slc25a15 209
Ddah1 848
Putrescine
Arginine
Ass1 128
citrate
Got1 84
Pyruvat
Mod1 78
NA
Pbef1 327
NAM
NADPH
Taldo1 160
Ribose
Eno1 22
Pkm2 247
Pyruvate
SAM
Srm 3
Spermidine
DMA
NOS
Nucleus
Nrf2
Pmf1 189
Pparg 592
C/EBP
Xanthine
Ddit3 498
Xdh 381
Urate
HNF-4
RXR
Nrip1 8
Nr1d2 (301)
Thra (436)
ER
Sod1 305
Dbi 323
dFA
Elovl6 162
H6pd 533
Cholesterol Biosynthesis
Dhcr7 299
Cholesterol
Abca1 290
HDL
ApoD 15
Ppap2b 367
Ppap2c 853
MAG, DAG
LD
Adfp 201
Retinacid
retinal
Retinol
Aldh1a1 24
Dhrs3 385
dTMP
Thymidine
Tk1 165
Psph 281
Serine
Slc7a3 72
Lysine arginine
Slc5a6 105
Vitamins
T3
T4S
T3S
Sult1a1 375
Purin
Ppat 287
Bh4
Gch1 259
Scarb1 397
Slc1a3 122
Glutamate
+Nh4 2+
Gln
Glul 318
Cdo1 271
Methionine
Adenosine + homocysteine
Cys
THF
DHF
Dhfr 161
Folat
PGE2
Mm103681 600
Na/vit. C ?
Mat2a 380
Suv39h1 113 ?
Ahcy (86)
4631427C17Rik 293
Sulfat
Taurin
Na/phosphate
SAH
dCMP
Deoxycytidin
Dck 363
PGH2
2410099E23 539/540
Slc20a1 179
Hydrophobic amphipathic drugs
As3mt 70
C1
dNDP
NDP
Rrm2 448
Abcb1a 204
ATP
Lypla3 172
AC
Lysosome
Peroxisome
H2O2
H2O
Cat 289
Na/Ca
Slc8a1 177
Aqp1 319
Water
Slc38a4 196

14d

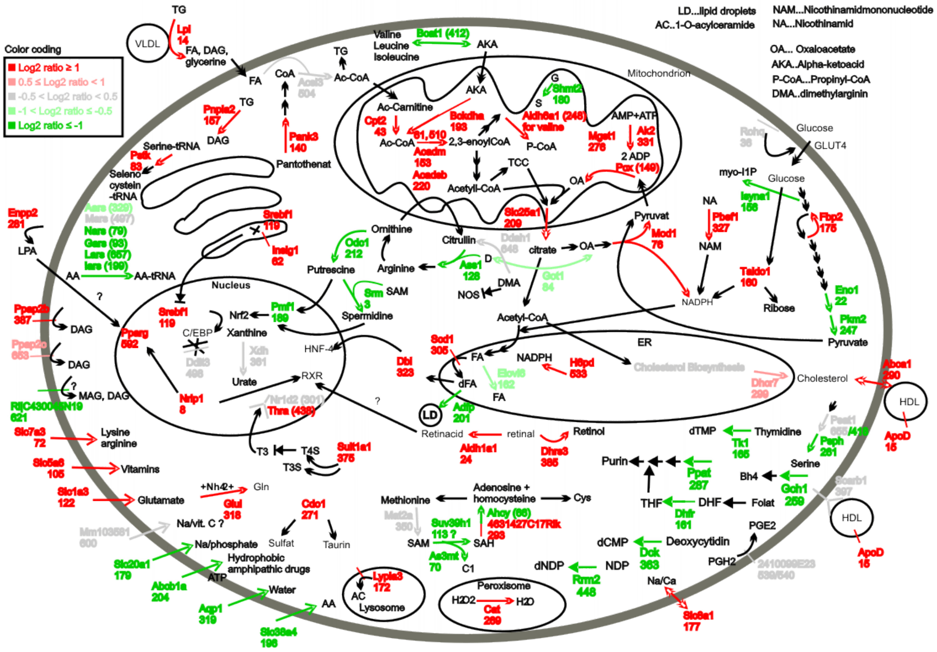
Color coding
Log2 ratio ≥ 1
0.5 ≤ Log2 ratio < 1
-0.5 < Log2 ratio < 0.5
-1 < Log2 ratio ≤ -0.5
Log2 ratio ≤ -1
LD...lipid droplets
AC..1-O-acylceramide
NAM...Nicothinamidmononucleotide
NA...Nicothinamid
OA... Oxaloacetate
AKA..Alpha-ketoacid
P-CoA...Propinyl-CoA
DMA..dimethylarginin
TG
Lpl 14
VLDL
FA, DAG, glycerine
Valine Leucine Isoleucine
Bcat1 (412)
AKA
CoA
Acss3 804
Ac-CoA
Mitochondrion
Ac-Carnitine
Cpt2 43
81,810
Bckdha 193
2,3-enoylCoA
Aldh6a1 (248) for valine
P-CoA
Acadm 153
Acadsb 220
Acetyl-CoA
TCC
OA
Shmt2 180
AMP+ATP
Ak2 331
Mgst1 276
2 ADP
Pcx (149)
Rohc 38
Glucose
GLUT4
myo-I1P
Isyna1 158
Fbp2 175
Pnpla2 157
DAG
Pank3 140
Pantothenat
Serine-tRNA
Pstk 83
Seleno cystein -tRNA
Enpp2 281
LPA
Aars (329)
Mars (497)
Nars (79)
Gars (93)
Lars (857)
Iars (199)
AA
AA-tRNA
Srebf1 119
Insig1 62
Ornithine
Odc1 212
Citrullin
Slc25a15 209
Ddah1 848
Putrescine
Arginine
Ass1 128
citrate
Got1 84
Pyruvat
Mod1 78
NA
Pbef1 327
NAM
NADPH
Taldo1 160
Ribose
Eno1 22
Pkm2 247
Pyruvate
SAM
Srm 3
Spermidine
DMA
NOS
Nucleus
Nrf2
Pmf1 189
Pparg 592
C/EBP
Xanthine
Ddit3 498
Xdh 381
Urate
HNF-4
RXR
Nrip1 8
Nr1d2 (301)
Thra (436)
ER
Sod1 305
Dbi 323
dFA
Elovl6 162
H6pd 533
Cholesterol Biosynthesis
Dhcr7 299
Cholesterol
Abca1 290
HDL
ApoD 15
Ppap2b 367
Ppap2c 853
MAG, DAG
LD
Adfp 201
Retinacid
retinal
Retinol
Aldh1a1 24
Dhrs3 385
dTMP
Thymidine
Tk1 165
Psph 281
Serine
Slc7a3 72
Lysine arginine
Slc5a6 105
Vitamins
T3
T4S
T3S
Sult1a1 375
Purin
Ppat 287
Bh4
Gch1 259
Scarb1 397
Slc1a3 122
Glutamate
+Nh4 2+
Gln
Glul 318
Cdo1 271
Methionine
Adenosine + homocysteine
Cys
THF
DHF
Dhfr 161
Folat
PGE2
Mm103681 600
Na/vit. C ?
Mat2a 380
Suv39h1 113 ?
Ahcy (86)
4631427C17Rik 293
Sulfat
Taurin
Na/phosphate
SAH
dCMP
Deoxycytidin
Dck 363
PGH2
2410099E23 539/540
Slc20a1 179
Hydrophobic amphipathic drugs
As3mt 70
C1
dNDP
NDP
Rrm2 448
Abcb1a 204
ATP
Lypla3 172
AC
Lysosome
Peroxisome
H2O2
H2O
Cat 289
Na/Ca
Slc8a1 177
Aqp1 319
Water
Slc38a4 196
