# Supplementary material for: Molecular processes during fat cell development revealed by gene expression profiling and functional annotation
Source: Genome Biol. 2005 Dec 19;6(13):R108. doi: 10.1186/gb-2005-6-13-r108 (PMC1414107; doi:10.1186/gb-2005-6-13-r108)
Supplement: Additional data file 35 — Text describing signaling networks [file gb-2005-6-13-r108-S35.pdf]

Signaling networks

Important proteins of signaling networks are found to be specifically expressed during adipogenesis (Fig. 4). Known and hypothetical functional aspects of transcriptionally regulated targets with relevance for adipogenesis are discussed below.

Known regulators of cell division are not repressed only during the clonal expansion phase. Activator of S phase kinase (Ask, No. 359) is the regulatory subunit of Cdc7 kinase and recruits it to the minichromosome maintenance protein complex and the origin recognition complex. The Cdc7 kinase activity is co-regulated with the Ask expression [1]. Vaccinia related-kinase 1 (Vrk1, No. 267) is shown to phosphorylate p53 and considered to act as a switch controlling p53 binding partners [2]. Many known GTPase-pathway associated genes have been measured as highly expressed after induction. T-lymphoma invasion and metastasis 1 (Tiam1, No. 159) is a guanine nucleotide exchange factor (GEF) of the small GTPase Rac1, which regulates actin cytoskeleton, morphology and adhesion and antagonizes RhoA signaling [3,4]. Additionally, the putative constitutive active Rho GTPase Wnt1 responsive Cdc42 homolog (Wrch-1, No. 292), which has no detectable intrinsic GTPase activity and very high nucleotide exchange capacity, leads to an up-rounded phenotype [5,6]. Interplay between Wrch-1 and Tiam1, which might reverse the Wrch-1 activity through Rac1 signaling [5], could be a regulatory mechanism of cell morphology in adipogenesis. The small GTPase Rab1 (No. 325) regulates the transport of newly synthesized proteins from the rough endoplasmic reticulum to the Golgi [7,8]. Ras GTPase-activating protein III (GAPIII, No. 63) is a negative regulator of ras, which controls proliferation and differentiation in many cell [9]. We find the regulator of G-protein signaling 2 (Rgs2, No. 44) permanently up-regulated. Its high expression has been observed during early adipogenesis [10].

16 receptors are strongly transcriptionally regulated during adipogenesis (Fig. 3). Further proteins, which ensure the availability and function of receptors, have a distinct expression profile. Low density lipoprotein receptor-related protein associated protein 1 (Lrpap1, No. 28) and receptor (calcitonin) activity modifying protein 2 (Ramp2, No. 266) are both in the mainly up-regulated cluster 9. Lrpap1 prevents co-expressed ligands to bind prematurely to the LDL-receptor family during procession and supports receptor folding [11-13]. Ramp2 controls the transport and glycosylation of the calcitonin receptor, which modifies the receptor to be adrenomedullin specific [14]. We find the peptide hormone adrenomedullin (AM, No. 314) strongly repressed after 12 hours. This contrasts a recent observation of a U-shaped regulation profile [15]. Nuclear receptor interacting protein 1 (RIP140, No. 8) can bind to adipogenic transcription factors PPAR and RXR [16,17] and can regulate energy homeostasis in white adipose tissue [18]. Parallel to transforming growth factor β 3 (TGFbeta3, No. 574), decorin (No. 137/623) is strongly overexpressed during whole adipogenesis. Although expression of TGFbeta3 was found in mature adipose tissue [19], TGF-β is a potent inhibitor of adipogenesis [20]. Beside stabilizing fibrils and orienting fibrillogenesis [21], the protein core of decorin can bind TGF-β [22]. On the one hand, this interaction can repress myogenesis [23] and, on the other, it can reverse repressive effects of autocrine TGF-β on mouse macrophage activation [24]. This mechanism might be important for adipogenic differentiation.

Possibly, up-regulation of Sult1a1 links thyroid hormone deactivation with increased lipogenic activity. Sulfotransferase, phenol preferring 1 (Sult1a1, No. 375) is the functional ortholog to the human SULT1A1. The latter is described to transfer sulphate to the thyroid hormone and to detoxify the cell [25] (it should be noted that the rat homologue is not known to use TH as substrate [26]). Sulphated T4S blocks the conversion to the bioactive T3 completely and enhances the conversion to the receptor-inactive reverse T3 (rT3) [27]. Hypothyroid concentrations (possibly mediated by Sult1a1) stimulate differentiation and the activity of lipogenic enzymes, whereas hyperthyroidism enhances thermogenesis and oxygen consumption [28,29]. Finally, TH receptor α (Thra, No. 438) might compete with PPAR for RXR if TH is present [30].

References

1. Jiang W, McDonald D, Hope TJ, Hunter T: **Mammalian Cdc7-Dbf4 protein kinase complex is essential for initiation of DNA replication.** *EMBO J* 1999, **18:**5703-5713.

2. Vega FM, Sevilla A, Lazo PA: **p53 Stabilization and accumulation induced by human vaccinia-related kinase 1.** *Mol Cell Biol* 2004, **24:**10366-10380.

3. Leeuwen FN, Kain HE, Kammen RA, Michiels F, Kranenburg OW, Collard JG: **The guanine nucleotide exchange factor Tiam1 affects neuronal morphology; opposing roles for the small GTPases Rac and Rho.** *J Cell Biol* 1997, **139:**797-807.

4. Sander EE, ten Klooster JP, van Delft S, van der Kammen RA, Collard JG: **Rac downregulates Rho activity: reciprocal balance between both GTPases determines cellular morphology and migratory behavior.** *J Cell Biol* 1999, **147:**1009-1022.

5. Saras J, Wollberg P, Aspenstrom P: **Wrch1 is a GTPase-deficient Cdc42-like protein with unusual binding characteristics and cellular effects.** *Exp Cell Res* 2004, **299:**356-369.

6. Shutes A, Berzat AC, Cox AD, Der CJ: **Atypical mechanism of regulation of the Wrch-1 Rho family small GTPase.** *Curr Biol* 2004, **14:**2052-2056.

7. Huppertz C, Schurmann A, Joost HG: **Abundance and subcellular distribution of GTP-binding proteins in 3T3-L1 cells before and after differentiation to the insulin-sensitive phenotype.** *Eur J Biochem* 1993, **215:**611-617.

8. Muslin AJ: **Road Rage: Cardiac Rab1 and ER-to-Golgi Traffic.** *Circ Res* 2001, **89:**1087-1088.

9. Baba H, Fuss B, Urano J, Poullet P, Watson JB, Tamanoi F, Macklin WB: **GapIII, a new brain-enriched member of the GTPase-activating protein family.** *J Neurosci Res* 1995, **41:**846-858.

10. Imagawa M, Tsuchiya T, Nishihara T: **Identification of inducible genes at the early stage of adipocyte differentiation of 3T3-L1 cells.** *Biochem Biophys Res Commun* 1999, **254:**299-305.

11. Bu G, Maksymovitch EA, Geuze H, Schwartz AL: **Subcellular localization and endocytic function of low density lipoprotein receptor-related protein in human glioblastoma cells.** *J Biol Chem* 1994, **269:**29874-29882.

12. Bu G, Geuze HJ, Strous GJ, Schwartz AL: **39 kDa receptor-associated protein is an ER resident protein and molecular chaperone for LDL receptor-related protein.** *EMBO J* 1995, **14:**2269-2280.

13. Bu G, Rennke S: **Receptor-associated protein is a folding chaperone for low density lipoprotein receptor-related protein.** *J Biol Chem* 1996, **271:**22218-22224.

14. McLatchie LM, Fraser NJ, Main MJ, Wise A, Brown J, Thompson N, Solari R, Lee MG, Foord SM: **RAMPs regulate the transport and ligand specificity of the calcitonin-receptor-like receptor.** *Nature* 1998, **393:**333-339.

15. Fukai N, Yoshimoto T, Sugiyama T, Ozawa N, Sato R, Shichiri M, Hirata Y: **Concomitant expression of adrenomedullin and its receptor components in rat adipose tissues.** *Am J Physiol Endocrinol Metab* 2005, **288:**E56-E62.

16. Treuter E, Albrektsen T, Johansson L, Leers J, Gustafsson JA: **A regulatory role for RIP140 in nuclear receptor activation.** *Mol Endocrinol* 1998, **12:**864-881.

17. Lee CH, Wei LN: **Characterization of receptor-interacting protein 140 in retinoid receptor activities.** *J Biol Chem* 1999, **274:**31320-31326.

18. Leonardsson G, Steel JH, Christian M, Pocock V, Milligan S, Bell J, So PW, Medina-Gomez G, Vidal-Puig A, White R et al.: **Nuclear receptor corepressor RIP140 regulates fat accumulation.** *Proc Natl Acad Sci U S A* 2004, **101:**8437-8442.

19. Miller DA, Lee A, Matsui Y, Chen EY, Moses HL, Derynck R: **Complementary DNA cloning of the murine transforming growth factor-beta 3 (TGF beta 3) precursor and the comparative expression of TGF beta 3 and TGF beta 1 messenger RNA in murine embryos and adult tissues.** *Mol Endocrinol* 1989, **3:**1926-1934.

20. Choy L, Derynck R: **Transforming growth factor-beta inhibits adipocyte differentiation by Smad3 interacting with CCAAT/enhancer-binding protein (C/EBP) and repressing C/EBP transactivation function.** *J Biol Chem* 2003, **278:**9609-9619.

21. Scott JE: **Proteodermatan and proteokeratan sulfate (decorin, lumican/fibromodulin) proteins are horseshoe shaped. Implications for their interactions with collagen.** *Biochemistry* 1996, **35:**8795-8799.

22. Hildebrand A, Romaris M, Rasmussen LM, Heinegard D, Twardzik DR, Border WA, Ruoslahti E: **Interaction of the small interstitial proteoglycans biglycan, decorin and fibromodulin with transforming growth factor beta.** *Biochem J* 1994, **302 (Pt 2):**527-534.

23. Riquelme C, Larrain J, Schonherr E, Henriquez JP, Kresse H, Brandan E: **Antisense inhibition of decorin expression in myoblasts decreases cell responsiveness to transforming growth factor beta and accelerates skeletal muscle differentiation.** *J Biol Chem* 2001, **276:**3589-3596.

24. Comalada M, Cardo M, Xaus J, Valledor AF, Lloberas J, Ventura F, Celada A: **Decorin reverses the repressive effect of autocrine-produced TGF-beta on mouse macrophage activation.** *J Immunol* 2003, **170:**4450-4456.

25. Jakoby WB, Ziegler DM: **The enzymes of detoxication.** *J Biol Chem* 1990, **265:**20715-20718.

26. Kester MH, Kaptein E, Roest TJ, van Dijk CH, Tibboel D, Meinl W, Glatt H, Coughtrie MW, Visser TJ: **Characterization of rat iodothyronine sulfotransferases.** *Am J Physiol Endocrinol Metab* 2003, **285:**E592-E598.

27. Visser TJ: **Role of sulfation in thyroid hormone metabolism.** *Chem Biol Interact* 1994, **92:**293-303.

28. Hellstrom L, Wahrenberg H, Reynisdottir S, Arner P: **Catecholamine-induced adipocyte lipolysis in human hyperthyroidism.** *J Clin Endocrinol Metab* 1997, **82:**159-166.

29. Flores-Delgado G, Marsch-Moreno M, Kuri-Harcuch W: **Thyroid hormone stimulates adipocyte differentiation of 3T3 cells.** *Mol Cell Biochem* 1987, **76:**35-43.

30. Berrodin TJ, Marks MS, Ozato K, Linney E, Lazar MA: **Heterodimerization among thyroid hormone receptor, retinoic acid receptor, retinoid X receptor, chicken ovalbumin upstream**

promoter transcription factor, and an endogenous liver protein. *Mol Endocrinol* 1992, **6:**1468-1478.
