# Supplementary material for: Molecular processes during fat cell development revealed by gene expression profiling and functional annotation
Source: Genome Biol. 2005 Dec 19;6(13):R108. doi: 10.1186/gb-2005-6-13-r108 (PMC1414107; doi:10.1186/gb-2005-6-13-r108)
Supplement: Additional data file 36 — A text file describing extracellular matrix remodeling and cytoskeleton reorganization [file gb-2005-6-13-r108-S36.pdf]

Extracellular matrix remodeling and cytoskeletal reorganization

Remodeling of the extracellular matrix and cytoskeletal changes are common to many cellular processes. Changes during adipogenesis (phenotypically seen as rounding of densely packed cells) have common aspects with other tissue differentiation processes such as endothelial angiogenesis (protease, collagen and non-collagen molecule secretion) [1] and specific features.

Matrix metalloproteinase 2 (MMP-2, No. 342) was strongly up-regulated during whole adipocyte differentiation and can cleave different collagen structures and its inhibition can block adipogenesis [2]. Tissue inhibitor of metalloproteinase 2 (Timp2 No. 239), a known partner of MMP-2, which balances the activity of the proprotease/protease [3] was mainly up regulated. Decreased Timp3 (No. 81, up-regulated at 6h, repressed after 12h) levels are associated with obese mice [4].

New collagen structures of overexpressed Col6a2 (No. 11), Col4a1 (No. 58) and Col4a2 (No. 303) [5] are crosslinked by the lysyl oxidase (Lox, No. 282, up-regulated during adipogenesis (contrary to [6])). Strongly up-regulated decorin (No. 137/623) and osteoblast specific factor 2 (Osf-2, No. 183) as well as proline arginine-rich end leucine-rich repeats (LRR, No. 73/390/484, up in final stages) attach the matrix to the cell. Matrillin-2 (Matn2, No. 12, up-regulated during adipogenesis) functions as adaptor for non-collagen structures [7] as nidogen 2 (Nid2, No. 294 increasingly up-regulated). Secreted protein acidic and rich in cysteine/osteonectin (SPARC, No. 67, mainly up-regulated) and SPARC-like 1 (Sparcl1, No. 154, up-regulated at 0h, 72h, 7d and 14d) can organize extracellular matrix remodeling, inhibits cell cycle progression and induces cell rounding in cultured cells [8,9].

Most of these cytoskeletal proteins can be found co-expressed in cluster 10 (not repressed 6h~12h) and might have a common regulatory mechanism. Transcription of Actin α and gamma, tubulin α (Tuba4, No. 377) and β (Tubb5, no. 110) and vimentin are found to diminish during differentiation in agreement with literature [10]. Myosin light chain 2 (Mylc2b/Mlc2, No. 87/88/58/421), tropomyosin 1 and 2 (Tpm1/Tpm2, No. 66/74) are members of the mainly repressed cluster 10. The down-regulated transgelin 1 and 2 (Tagln/Tagln2, No. 114/242) as well as fascin homolog 1 (Fscn1, No. 30) are known actin-bundling proteins [11,12]. Apparently, their absence decreases the cross-linking of microfilaments in compact parallel bundles. Calponin 2 (Cnn2, No. 7), a regulator of cytokinesis, is down-regulated [13].

The insulin receptor and actin binding proteins filamin α and β (Flna/Flnb, No. 506/632) can selectively inhibit the MAPK signaling cascade of the insulin receptor [14]. Finally, the maintenance protein ankycorbin (No. 59) and the cross-linking protein actinin 1 (Actn1, No. 521) share the mainly repressed expression profile. Tubulin γ1 (Tubg1, No. 78, up-regulated during adipogenesis, ~42-fold at 72h) is not a component of the microtululus as Tuba/b but has a role in organizing the assembly and in establishing cell polarity [15].

Actinin 4 (Actn4, No. 185, up-regulated during whole time course), differs from Actn1 in localization, its expression leads to higher cell motility and it can be translocated into the nucleus upon PI-3-kinase inhibition [16]. Adducin 3 γ (Add3, No. 50, permanently up-regulated) has different actin-associated cytoskeletal roles.

Further, the key signaling components Tiam1 and Wrch1 are transcriptionally up-regulated as described above. The reduced replenishment of the cytoskeleton with building blocks and the strong transcriptional up-regulation of modulating proteins together with the extracellular remodeling might cause the morphological changes during differentiation of 3T3-L1 cells.

References

1. Novatchkova M, Eisenhaber F: **Can molecular mechanisms of biological processes be extracted from expression profiles? Case study: endothelial contribution to tumor-induced angiogenesis.** *Bioessays* 2001, **23:**1159-1175.

2. Croissandeau G, Chretien M, Mbikay M: **Involvement of matrix metalloproteinases in the adipose conversion of 3T3-L1 preadipocytes.** *Biochem J* 2002, **364:**739-746.

3. Karagiannis ED, Popel AS: **A theoretical model of type I collagen proteolysis by matrix metalloproteinase (MMP) 2 and membrane type 1 MMP in the presence of tissue inhibitor of metalloproteinase 2.** *J Biol Chem* 2004, **279:**39105-39114.

4. Chavey C, Mari B, Monthouel MN, Bonnafous S, Anglard P, Van Obberghen E, Tartare-Deckert S: **Matrix metalloproteinases are differentially expressed in adipose tissue during obesity and modulate adipocyte differentiation.** *J Biol Chem* 2003, **M209196200:**1-42.

5. Weiner FR, Shah A, Smith PJ, Rubin CS, Zern MA: **Regulation of collagen gene expression in 3T3-L1 cells. Effects of adipocyte differentiation and tumor necrosis factor alpha.** *Biochemistry* 1989, **28:**4094-4099.

6. Dimaculangan DD, Chawla A, Boak A, Kagan HM, Lazar MA: **Retinoic acid prevents downregulation of ras recision gene/lysyl oxidase early in adipocyte differentiation.** *Differentiation* 1994, **58:**47-52.

7. Piecha D, Wiberg C, Morgelin M, Reinhardt DP, Deak F, Maurer P, Paulsson M: **Matrilin-2 interacts with itself and with other extracellular matrix proteins.** *Biochem J* 2002, **367:**715-721.

8. Brekken RA, Sage EH: **SPARC, a matricellular protein: at the crossroads of cell-matrix.** *Matrix Biol* 2000, **19:**569-580.

9. Bradshaw AD, Sage EH: **SPARC, a matricellular protein that functions in cellular differentiation and tissue response to injury.** *J Clin Invest* 2001, **107:**1049-1054.

10. Spiegelman BM, Farmer SR: **Decreases in tubulin and actin gene expression prior to morphological differentiation of 3T3 adipocytes.** *Cell* 1982, **29:**53-60.

11. Edwards RA, Herrera-Sosa H, Otto J, Bryan J: **Cloning and expression of a murine fascin homolog from mouse brain.** *J Biol Chem* 1995, **270:**10764-10770.

12. Winder SJ, Jess T, Ayscough KR: **SCP1 encodes an actin-bundling protein in yeast.** *Biochem J* 2003, **375:**287-295.

13. Hossain MM, Hwang DY, Huang QQ, Sasaki Y, Jin JP: **Developmentally regulated expression of calponin isoforms and the effect of h2-calponin on cell proliferation.** *Am J Physiol Cell Physiol* 2003, **284:**C156-C167.

14. He HJ, Kole S, Kwon YK, Crow MT, Bernier M: **Interaction of filamin A with the insulin receptor alters insulin-dependent activation of the mitogen-activated protein kinase pathway.** *J Biol Chem* 2003, **278:**27096-27104.

15. Oakley BR: **Gamma-tubulin: the microtubule organizer?** *Trends Cell Biol* 1992, **2:**1-5.

16. Honda K, Yamada T, Endo R, Ino Y, Gotoh M, Tsuda H, Yamada Y, Chiba H, Hirohashi S: **Actinin-4, a novel actin-bundling protein associated with cell motility and cancer invasion.** *J Cell Biol* 1998, **140:**1383-1393.
