# Supplementary material for: Molecular processes during fat cell development revealed by gene expression profiling and functional annotation
Source: Genome Biol. 2005 Dec 19;6(13):R108. doi: 10.1186/gb-2005-6-13-r108 (PMC1414107; doi:10.1186/gb-2005-6-13-r108)

Experimental verified binding sites for PPARγ

Gene	Organism	5’-3’ Sequence	Ref
CYP4A6/P450 IV	rabbit	AACT AGGGCA A AGTTGA	[1]
CYP4A1/P450 IV	rat	AACT AGGGTA A AGTTCA	[2]
L-fatty acid binding protein	rat	ATAT AGGCCA T AGGTCA*	[3]
3-hydroxy-3-methyl-glutaryl-CoA-synthase	rat	AACT GGGCCA A AGGTCT*	[4]
Enoyl-CoA-hydratase	rat	ATGT AGGTAA T AGTTCA*	[1]
Malic enzyme	rat	TTCT GGGTCA A AGTTGA	[5]
Phosphoenolpyruvate carboxikinase	rat	AACT GGGATA A AGGTCT	[6]
Phosphoenolpyruvate carboxikinase)	rat	CCCA CGGCCA A AGGTCA*	[6]
Acyl-CoA oxidase	rat	GACC AGGACA A AGGTCA*	[1]
Liver specific type 1 sugar transporter	rat	TTAC AGGACA A AGGCCA	[7]
Malic enzyme	rat	TTAG AGGGCA C AGGTCC*	[5]
Acyl-CoA oxidase	rat	AGCA AGGTAG A AGGTCA*	[1]
Acyl-CoA synthetase	rat	TTTC AGGGCA T CAGTCA*	[8]
Palmytoiltransferase fatty acid transport	mouse	AAGT GGGGCA A AGGGCA	[9]
aP2 adipocyte lipid binding protein	mouse	CTCT GGGTGA A ATGTGC*	[10]
aP2 adipocyte lipid binding protein	mouse	TACT GGATCA G AGTTCA	[10]
c-Cbl-associating protein	mouse	ACAC AGGCTA A AGGTCA	[11]
Uncoupling protein 1	mouse	AGTG TGGTCA A GGGTGA*	[12]
Apolopoprotein C-III	human	GCGC TGGGCA A AGGTCA*	[1]
Acyl-CoA oxidase	human	TAGA AGGTCA G CTGTCA	[13]
Lipoprotein lipase	human	GTCT GCCCTT T CCCCCT*	[14]
Muscle type carnitine palmitoyltransferase I	human	CCTT TTCCCT A CATTTG	[15]
Consensus		AWCT AGGNCA A AGGTCA	[16]

* antisense sequence reported

References

1. Krey G, Keller H, Mahfoudi A, et al. **Xenopus peroxisome proliferator activated receptors: genomic organization, response element recognition, heterodimer formation with retinoid X receptor and activation by fatty acids**. *J Steroid Biochem Mol Biol* 1993; **47:**65-73.
2. Aldridge TC, Tugwood JD, Green S. **Identification and characterization of DNA elements implicated in the regulation of CYP4A1 transcription.** *Biochem J* 1995; **306:**473-9.
3. Issemann I, Prince R, Tugwood J, Green S. **A role for fatty acids and liver fatty acid binding protein in peroxisome proliferation.** *Biochem Soc Trans* 1992; **20**:824-7.

4. Rodriguez JC, Gil-Gomez G, Hegardt FG, Haro D. **Peroxisome proliferator-activated receptor mediates induction of the mitochondrial 3-hydroxy-3-methylglutaryl-CoA synthase gene by fatty acids.** *J Biol Chem* 1994; **269:**18767-72.

5. Castelein H, Gulick T, Declercq PE, Mannaerts GP, Moore DD, Baes MI. **The peroxisome proliferator activated receptor regulates malic enzyme gene expression.** *J Biol Chem* 1994; **269:**26754-8.

6. Tontonoz P, Hu E, Devine J, Beale EG, Spiegelman BM. **PPAR gamma 2 regulates adipose expression of the phosphoenolpyruvate carboxykinase gene.** *Mol Cell Biol* 1995; **15:**351-7.

7. Simonson GD, Iwanij V. **Genomic organization and promoter sequence of a gene encoding a rat liver-specific type-I transport protein.** *Gene* 1995, **154:**243-7.

8. Schoonjans K, Watanabe M, Suzuki H, et al. **Induction of the acyl-coenzyme A synthetase gene by fibrates and fatty acids is mediated by a peroxisome proliferator response element in the C promoter.** *J Biol Chem* 1995, **270:**19269-76.

9. Frohnert BI, Hui TY, Bernlohr DA. **Identification of a functional peroxisome proliferator-responsive element in the murine fatty acid transport protein gene.** *J Biol Chem* 1999; **274:**3970-7.

10. Tontonoz P, Hu E, Graves RA, Budavari AI, Spiegelman BM. **mPPAR gamma 2: tissue-specific regulator of an adipocyte enhancer.** *Genes Dev* 1994, **8:**1224-34.

11 Baumann CA, Chokshi N, Saltiel AR, Ribon V. **Cloning and characterization of a functional peroxisome proliferator activator receptor-gamma-responsive element in the promoter of the CAP gene.** *J Biol Chem* 2000, **275:**9131-5.

12. Sears IB, MacGinnitie MA, Kovacs LG, Graves RA. **Differentiation-dependent expression of the brown adipocyte uncoupling protein gene: regulation by peroxisome proliferator-activated receptor gamma.** *Mol Cell Biol* 1996, **16:**3410-9.

13. Varanasi U, Chu R, Huang Q, Castellon R, Yeldandi AV, Reddy JK. **Identification of a peroxisome proliferator-responsive element upstream of the human peroxisomal fatty acyl coenzyme A oxidase gene.** *J Biol Chem* 1996, **271:**2147-55.

14. Schoonjans K, Peinado-Onsurbe J, Lefebvre AM, et al. **PPARalpha and PPARgamma activators direct a distinct tissue-specific transcriptional response via a PPRE in the lipoprotein lipase gene.** *Embo J* 1996, **15:**5336-48.

15. Mascaro C, Acosta E, Ortiz JA, Marrero PF, Hegardt FG, Haro D. **Control of human muscle-type carnitine palmitoyltransferase I gene transcription by peroxisome proliferator-activated receptor**. *J Biol Chem* 1998, **273:**8560-3.

16. Juge-Aubry C, Pernin A, Favez T, et al. **DNA binding properties of peroxisome proliferator-activated receptor subtypes on various natural peroxisome proliferator response elements. Importance of the 5'-flanking region.** *J Biol Chem* 1997, **272:**25252-9.

Position specific weight matrix and sequence logo

	1	2	3	4	5	6	7	8	9	10	11	12	13	14	15	16	17
A	10	8	4	3	11	0	1	3	2	19	15	17	2	0	0	0	16
C	3	4	11	5	1	1	2	6	15	0	1	4	1	1	2	17	2
G	3	2	4	2	7	20	19	6	1	1	2	1	17	15	1	4	1
T	6	8	3	12	3	1	0	7	4	2	4	0	2	6	19	1	3

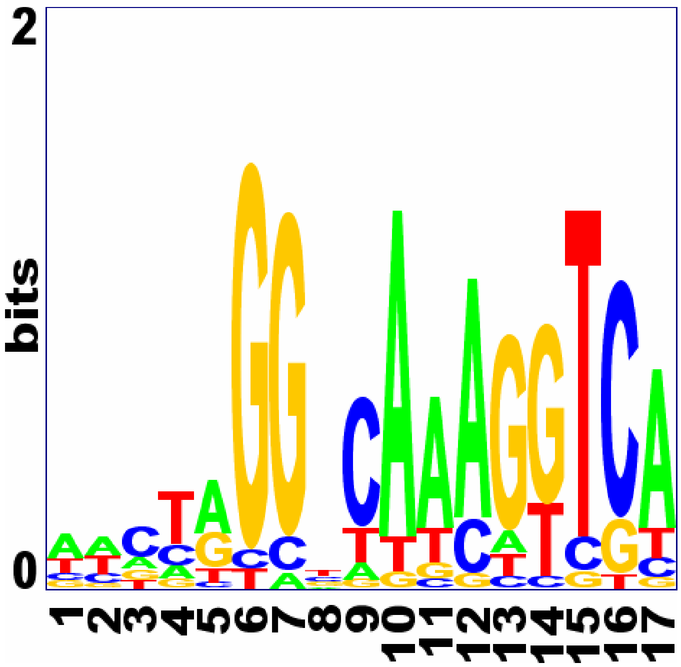

Supplement: Additional data file 39 — A list of experimental verified binding site for PPAR:RXR and the derived position weight matrix [file gb-2005-6-13-r108-S39.pdf]
