# Supplementary material for: Molecular processes during fat cell development revealed by gene expression profiling and functional annotation
Source: Genome Biol. 2005 Dec 19;6(13):R108. doi: 10.1186/gb-2005-6-13-r108 (PMC1414107; doi:10.1186/gb-2005-6-13-r108)
Supplement: Additional data file 42 — A file showing the promoter sequences in fasta format [file gb-2005-6-13-r108-S42.pdf]

Cluster 1

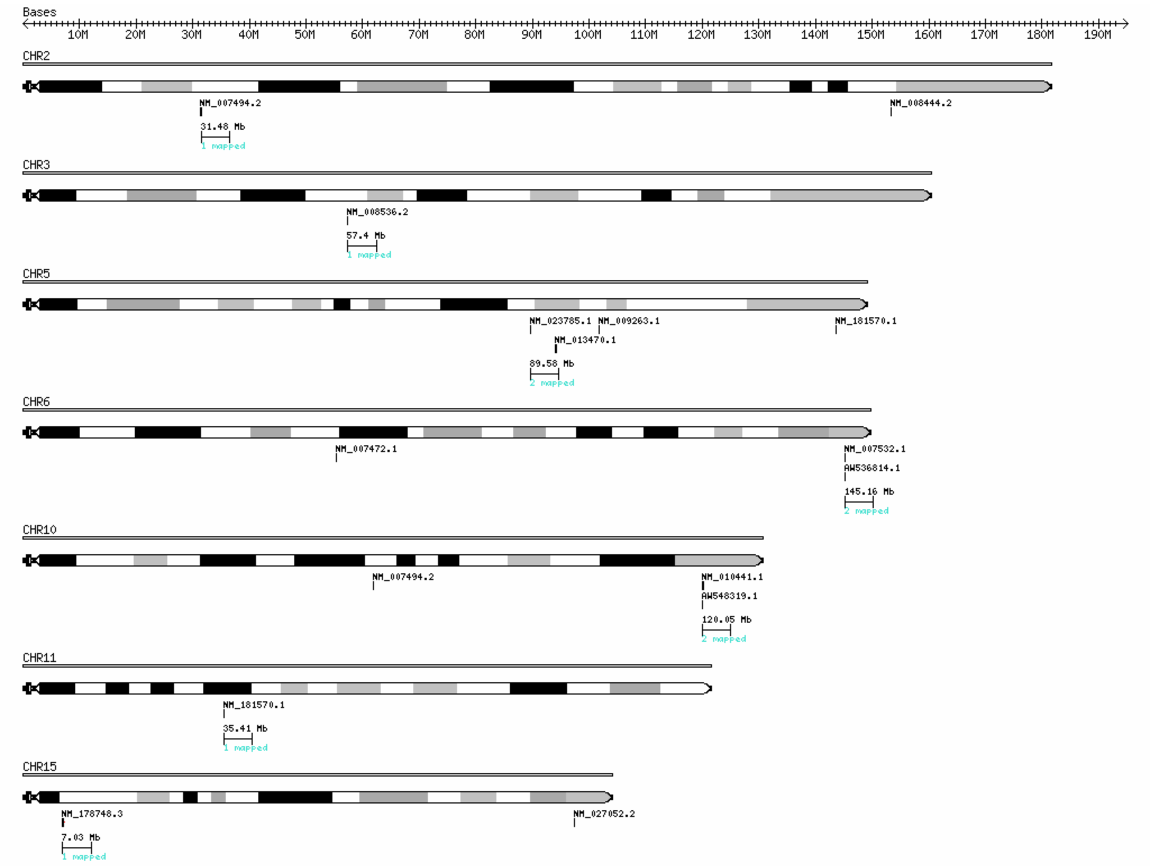
Bases
10M
20M
30M
40M
50M
60M
70M
80M
90M
100M
110M
120M
130M
140M
150M
160M
170M
180M
190M
CHR2
NM_007494.2
NM_008444.2
31.48 Mb
1 mapped
CHR3
NM_008536.2
57.4 Mb
1 mapped
CHR5
NM_023785.1
NM_009263.1
NM_181570.1
NM_013470.1
89.58 Mb
2 mapped
CHR6
NM_007472.1
NM_007532.1
AW536814.1
145.16 Mb
2 mapped
CHR10
NM_007494.2
NM_010441.1
AW548319.1
120.05 Mb
2 mapped
CHR11
NM_181570.1
35.41 Mb
1 mapped
CHR15
NM_178748.3
NM_027052.2
7.03 Mb
1 mapped

Cluster 2A

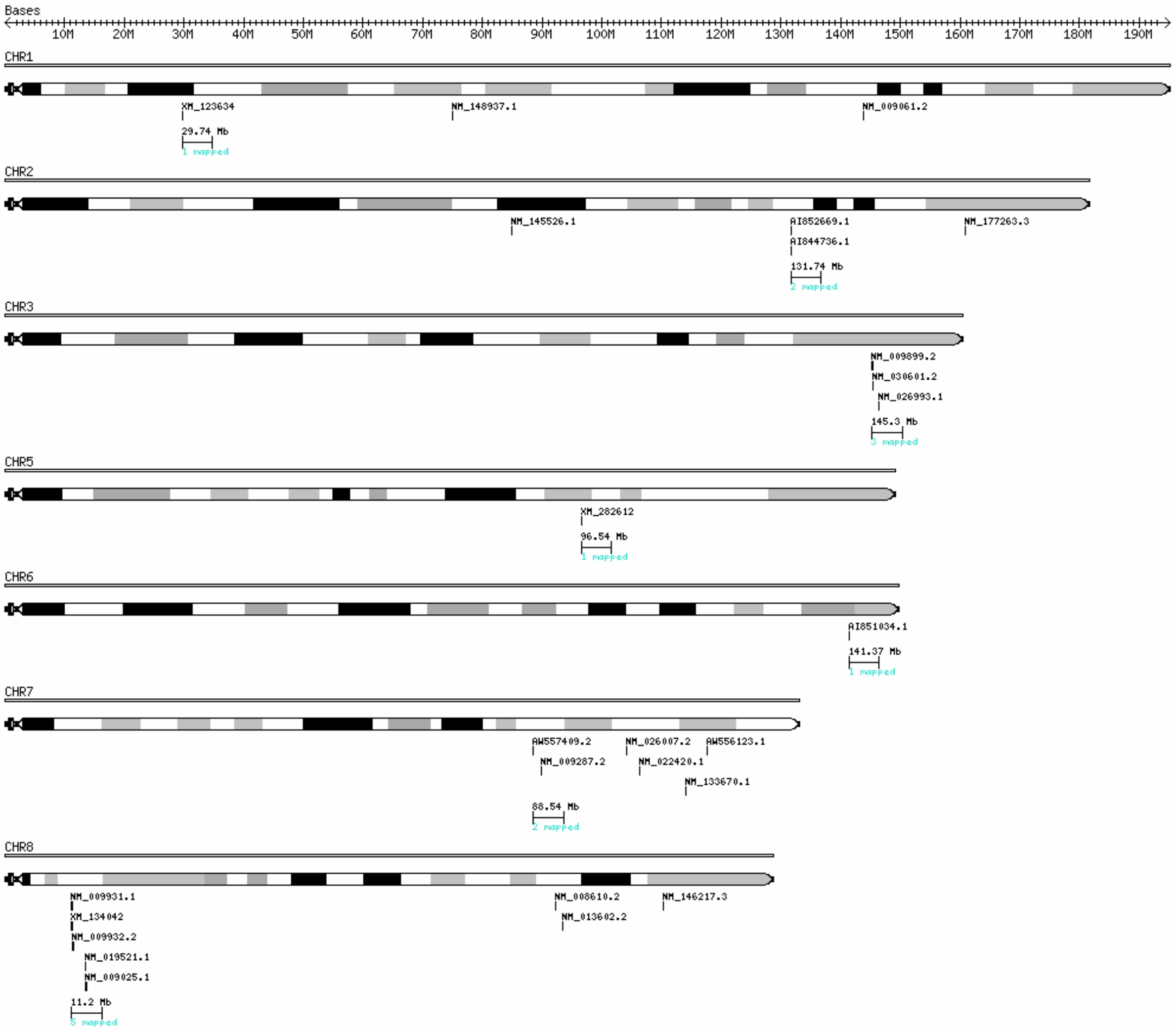
Bases
10M
20M
30M
40M
50M
60M
70M
80M
90M
100M
110M
120M
130M
140M
150M
160M
170M
180M
190M
CHR1
XM_123634
NM_148937.1
NM_009061.2
29.74 Mb
1 mapped
CHR2
NM_145526.1
AI852669.1
NM_177263.3
AI844736.1
131.74 Mb
2 mapped
CHR3
NM_009899.2
NM_030601.2
NM_026993.1
145.3 Mb
3 mapped
CHR5
XM_282612
96.54 Mb
1 mapped
CHR6
AI851034.1
141.37 Mb
1 mapped
CHR7
AW557409.2
NM_026007.2
AW556123.1
NM_009287.2
NM_022420.1
NM_133670.1
88.54 Mb
2 mapped
CHR8
NM_009931.1
NM_008610.2
NM_146217.3
XM_134042
NM_013602.2
NM_009932.2
NM_019521.1
NM_009025.1
11.2 Mb
5 mapped

Cluster 2B

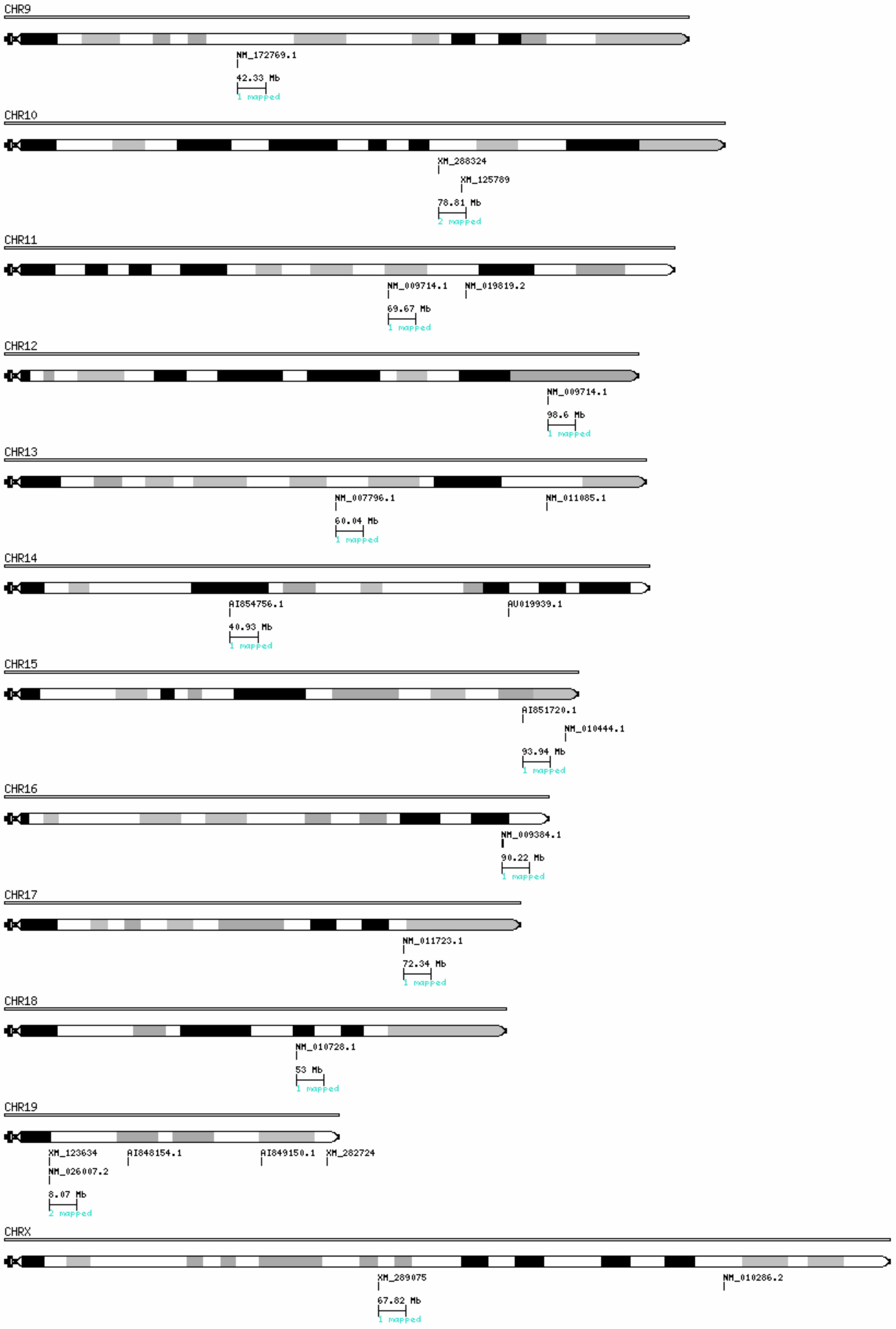
CHR9
NM_172769.1
42.33 Mb
1 mapped
CHR10
XM_288324
XM_125789
78.81 Mb
2 mapped
CHR11
NM_009714.1
NM_019819.2
69.67 Mb
1 mapped
CHR12
NM_009714.1
98.6 Mb
1 mapped
CHR13
NM_007796.1
NM_011085.1
60.04 Mb
1 mapped
CHR14
AI854756.1
AU019939.1
40.93 Mb
1 mapped
CHR15
AI851720.1
NM_010444.1
93.94 Mb
1 mapped
CHR16
NM_009384.1
90.22 Mb
1 mapped
CHR17
NM_011723.1
72.34 Mb
1 mapped
CHR18
NM_010728.1
53 Mb
1 mapped
CHR19
XM_123634
AI848154.1
AI849150.1
XM_282724
NM_026007.2
8.07 Mb
2 mapped
CHRX
XM_289075
NM_010286.2
67.82 Mb
1 mapped

Cluster 3

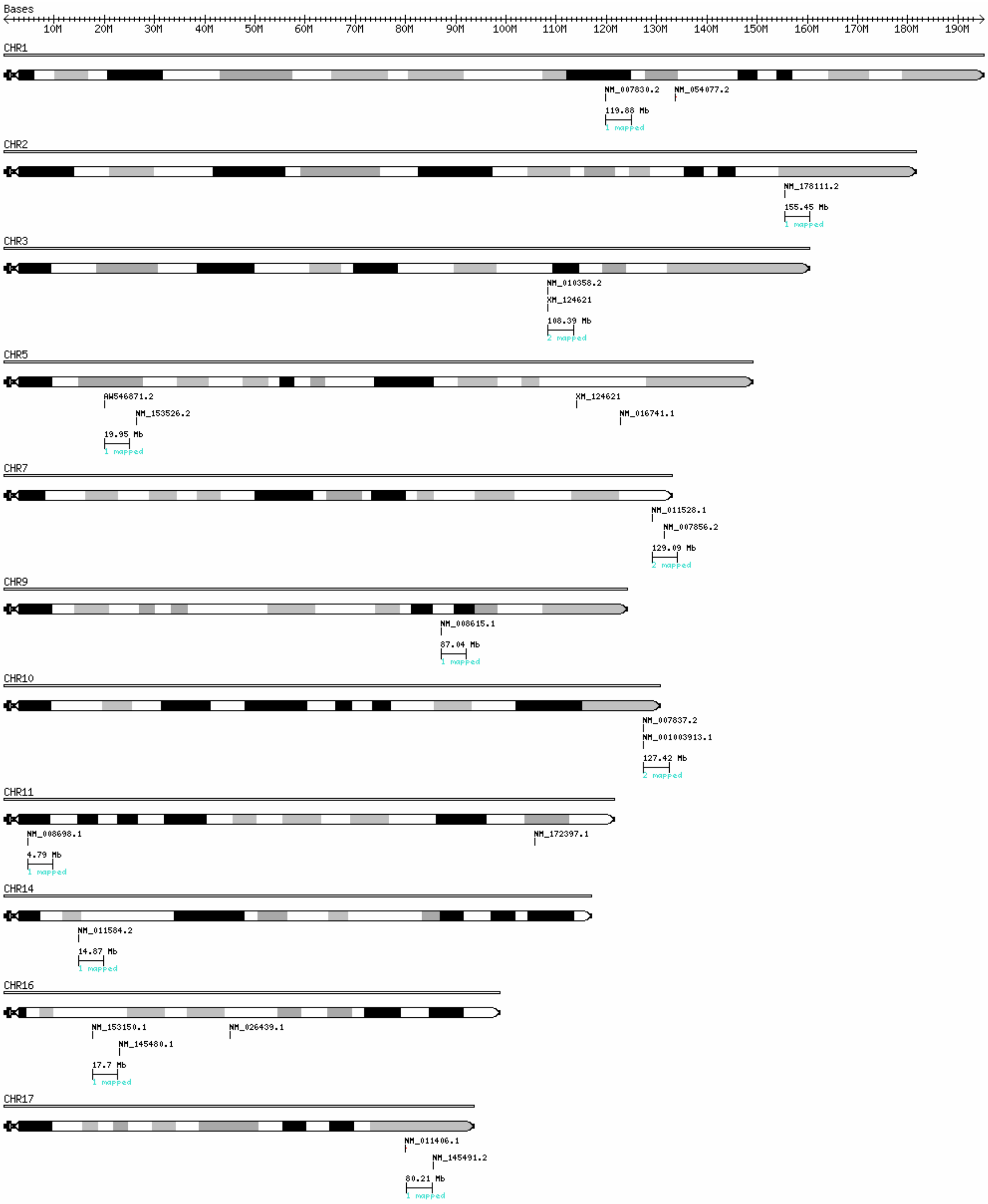

Bases
10M
20M
30M
40M
50M
60M
70M
80M
90M
100M
110M
120M
130M
140M
150M
160M
170M
180M
190M
CHR1
NM_007830.2
NM_054077.2
119.88 Mb
1 mapped
CHR2
NM_178111.2
155.45 Mb
1 mapped
CHR3
NM_010358.2
XM_124621
108.39 Mb
2 mapped
CHR5
AW546871.2
NM_153526.2
XM_124621
NM_016741.1
19.95 Mb
1 mapped
CHR7
NM_011528.1
NM_007856.2
129.09 Mb
2 mapped
CHR9
NM_008615.1
87.04 Mb
1 mapped
CHR10
NM_007837.2
NM_001003913.1
127.42 Mb
2 mapped
CHR11
NM_008698.1
NM_172397.1
4.79 Mb
1 mapped
CHR14
NM_011584.2
14.87 Mb
1 mapped
CHR16
NM_153150.1
NM_026439.1
NM_145480.1
17.7 Mb
1 mapped
CHR17
NM_011406.1
NM_145491.2
80.21 Mb
1 mapped

Cluster 4

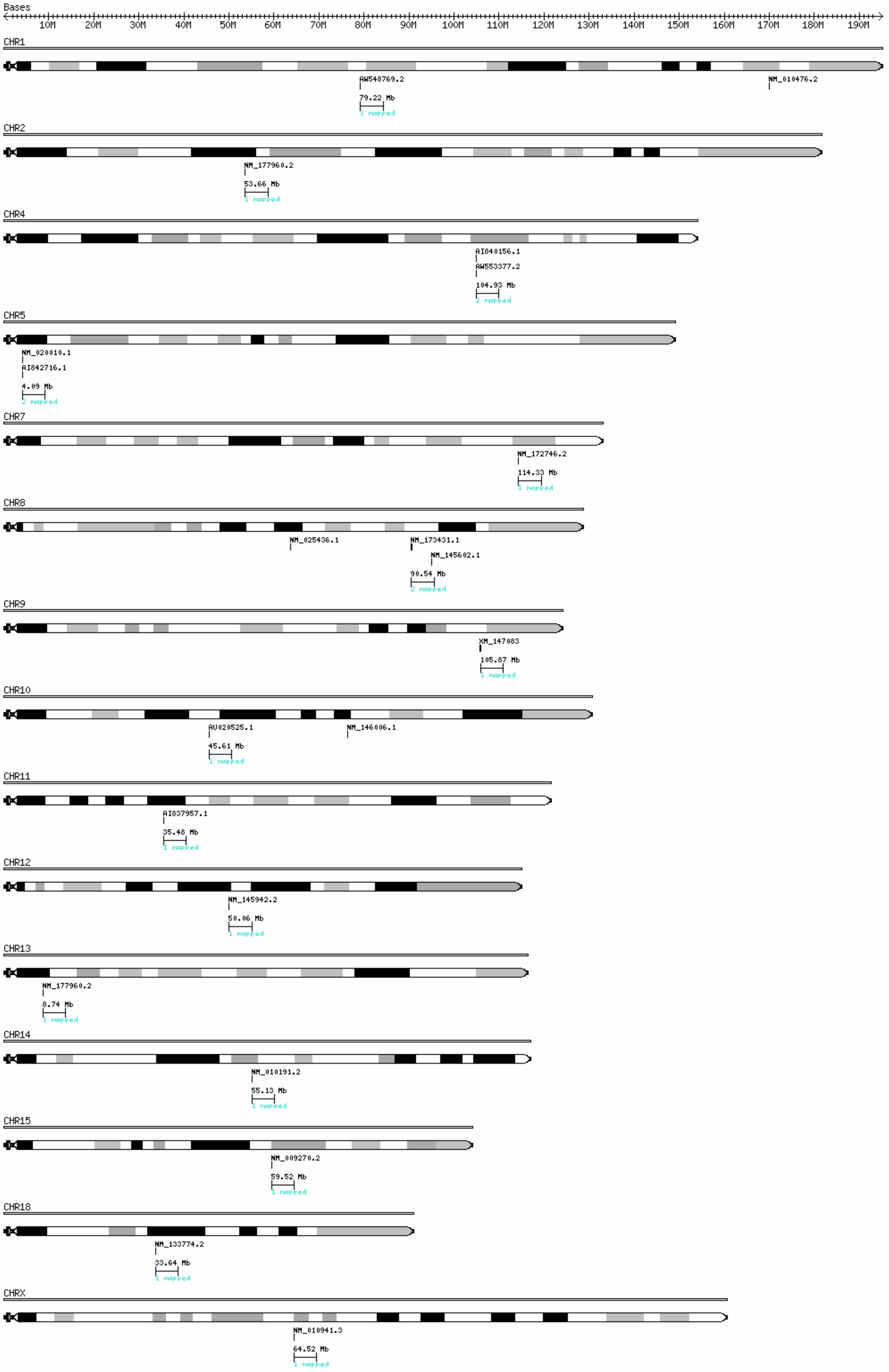
Bases
10M
20M
30M
40M
50M
60M
70M
80M
90M
100M
110M
120M
130M
140M
150M
160M
170M
180M
190M
CHR1
AW548769.2
NM_010476.2
79.22 Mb
1 mapped
CHR2
NM_177960.2
53.66 Mb
1 mapped
CHR4
AI840156.1
AW553377.2
104.93 Mb
2 mapped
CHR5
NM_020010.1
AI842716.1
4.09 Mb
2 mapped
CHR7
NM_172746.2
114.33 Mb
1 mapped
CHR8
NM_025436.1
NM_173431.1
NM_145602.1
90.54 Mb
2 mapped
CHR9
XM_147083
105.87 Mb
1 mapped
CHR10
AU020525.1
NM_146006.1
45.61 Mb
1 mapped
CHR11
AI837957.1
35.48 Mb
1 mapped
CHR12
NM_145942.2
50.06 Mb
1 mapped
CHR13
NM_177960.2
8.74 Mb
1 mapped
CHR14
NM_010191.2
55.13 Mb
1 mapped
CHR15
NM_009270.2
59.52 Mb
1 mapped
CHR18
NM_133774.2
33.64 Mb
1 mapped
CHRX
NM_010941.3
64.52 Mb
1 mapped

Cluster 5A

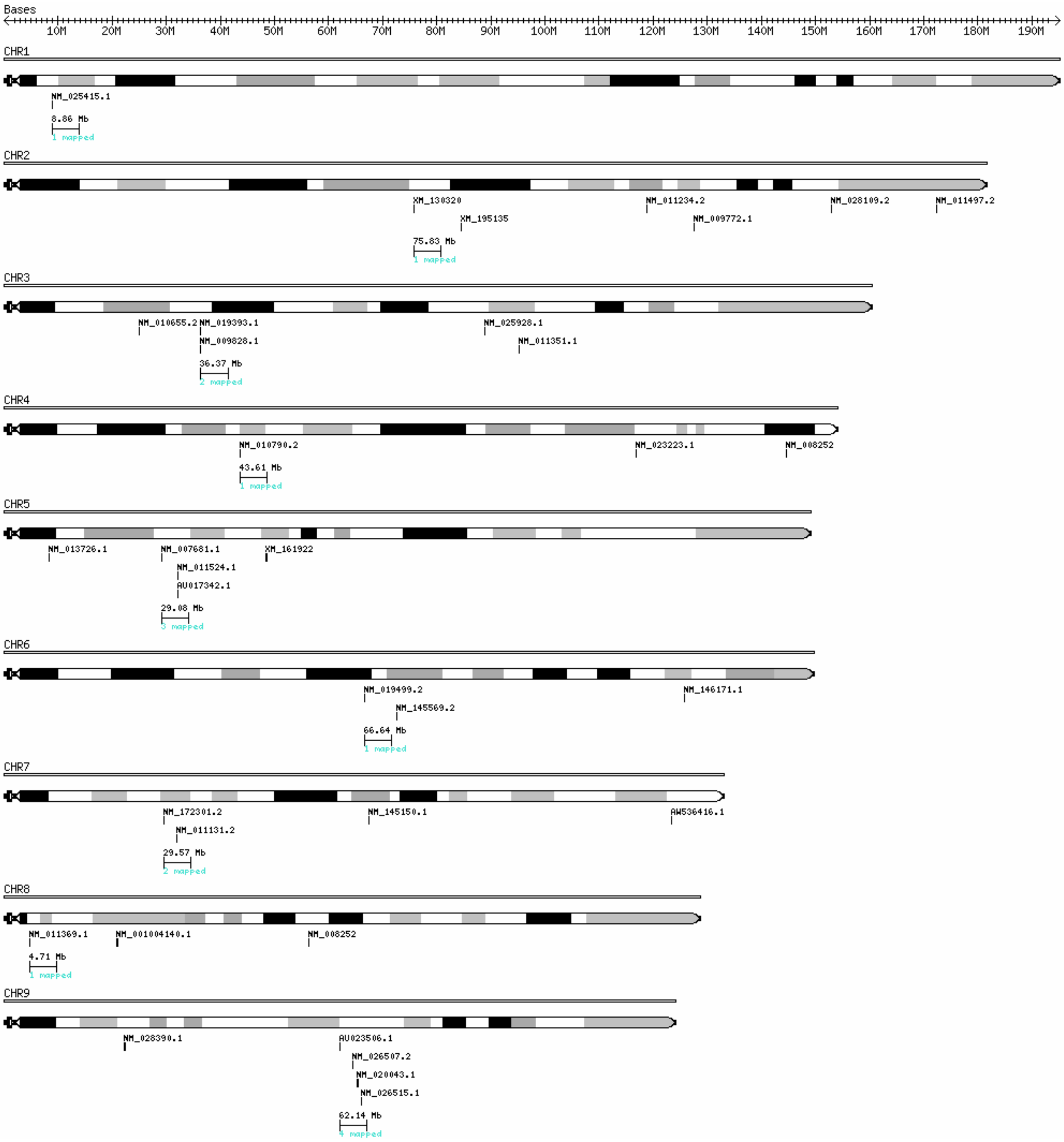

Bases
10M
20M
30M
40M
50M
60M
70M
80M
90M
100M
110M
120M
130M
140M
150M
160M
170M
180M
190M
CHR1
NM_025415.1
8.86 Mb
1 mapped
CHR2
XM_130320
XM_195135
NM_011234.2
NM_009772.1
NM_028109.2
NM_011497.2
75.83 Mb
1 mapped
CHR3
NM_010655.2
NM_019393.1
NM_009828.1
NM_025928.1
NM_011351.1
36.37 Mb
2 mapped
CHR4
NM_010790.2
NM_023223.1
NM_008252
43.61 Mb
1 mapped
CHR5
NM_013726.1
NM_007681.1
XM_161922
NM_011524.1
AU017342.1
29.08 Mb
3 mapped
CHR6
NM_019499.2
NM_145569.2
NM_146171.1
66.64 Mb
1 mapped
CHR7
NM_172301.2
NM_011131.2
NM_145150.1
AW536416.1
29.57 Mb
2 mapped
CHR8
NM_011369.1
NM_001004140.1
NM_008252
4.71 Mb
1 mapped
CHR9
NM_028390.1
AU023506.1
NM_026507.2
NM_020043.1
NM_026515.1
62.14 Mb
4 mapped

Cluster 5B

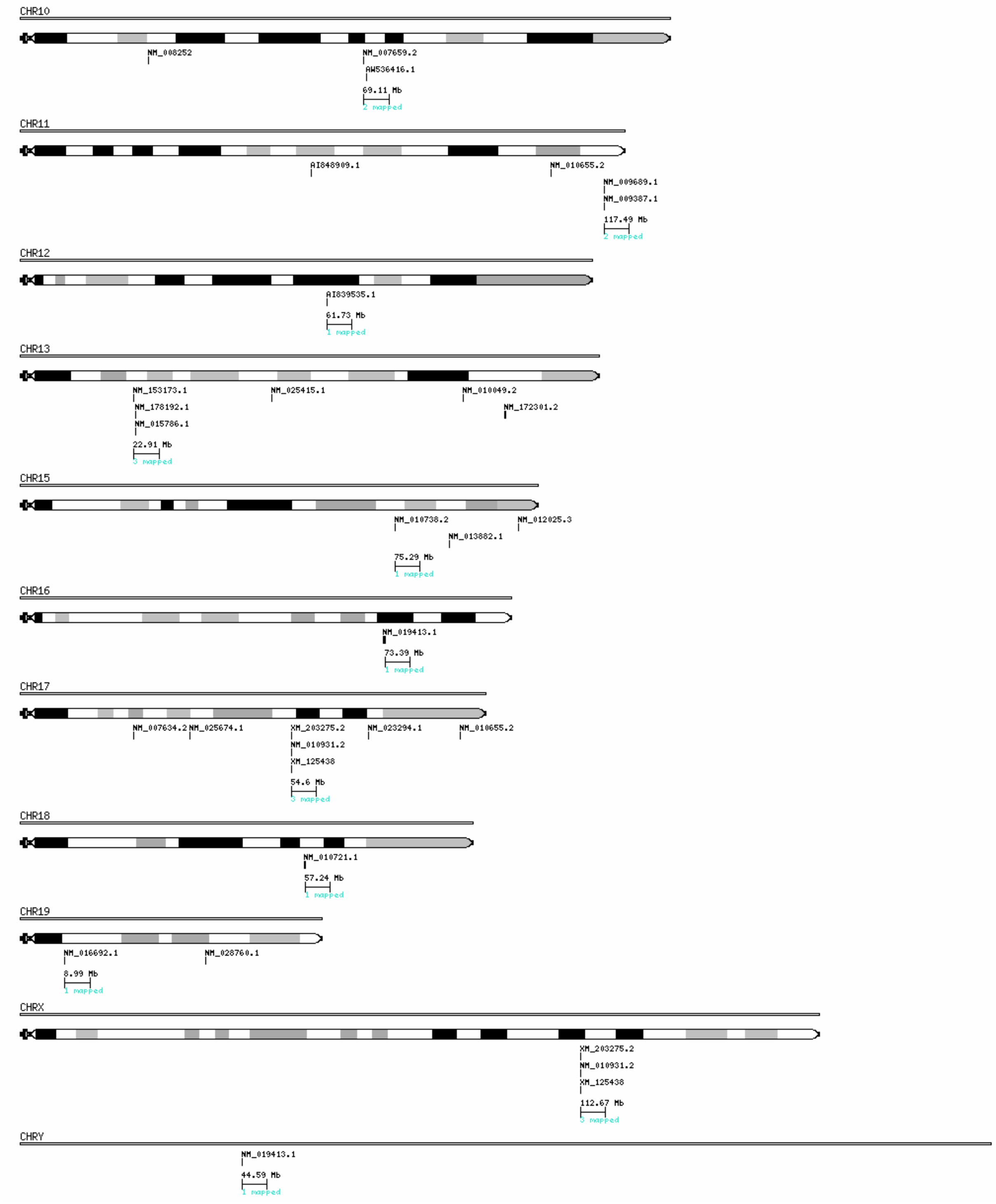
CHR10
NM_008252
NM_007659.2
AW536416.1
69.11 Mb
2 mapped
CHR11
AI848909.1
NM_010655.2
NM_009689.1
NM_009387.1
117.49 Mb
2 mapped
CHR12
AI839535.1
61.73 Mb
1 mapped
CHR13
NM_153173.1
NM_178192.1
NM_015786.1
NM_025415.1
NM_010049.2
NM_172301.2
22.91 Mb
3 mapped
CHR15
NM_010738.2
NM_012025.3
NM_013882.1
75.29 Mb
1 mapped
CHR16
NM_019413.1
73.39 Mb
1 mapped
CHR17
NM_007634.2
NM_025674.1
XM_203275.2
NM_010931.2
XM_125438
NM_023294.1
NM_010655.2
54.6 Mb
3 mapped
CHR18
NM_010721.1
57.24 Mb
1 mapped
CHR19
NM_016692.1
NM_028760.1
8.99 Mb
1 mapped
CHRX
XM_203275.2
NM_010931.2
XM_125438
112.67 Mb
3 mapped
CHRY
NM_019413.1
44.59 Mb
1 mapped

Cluster 6

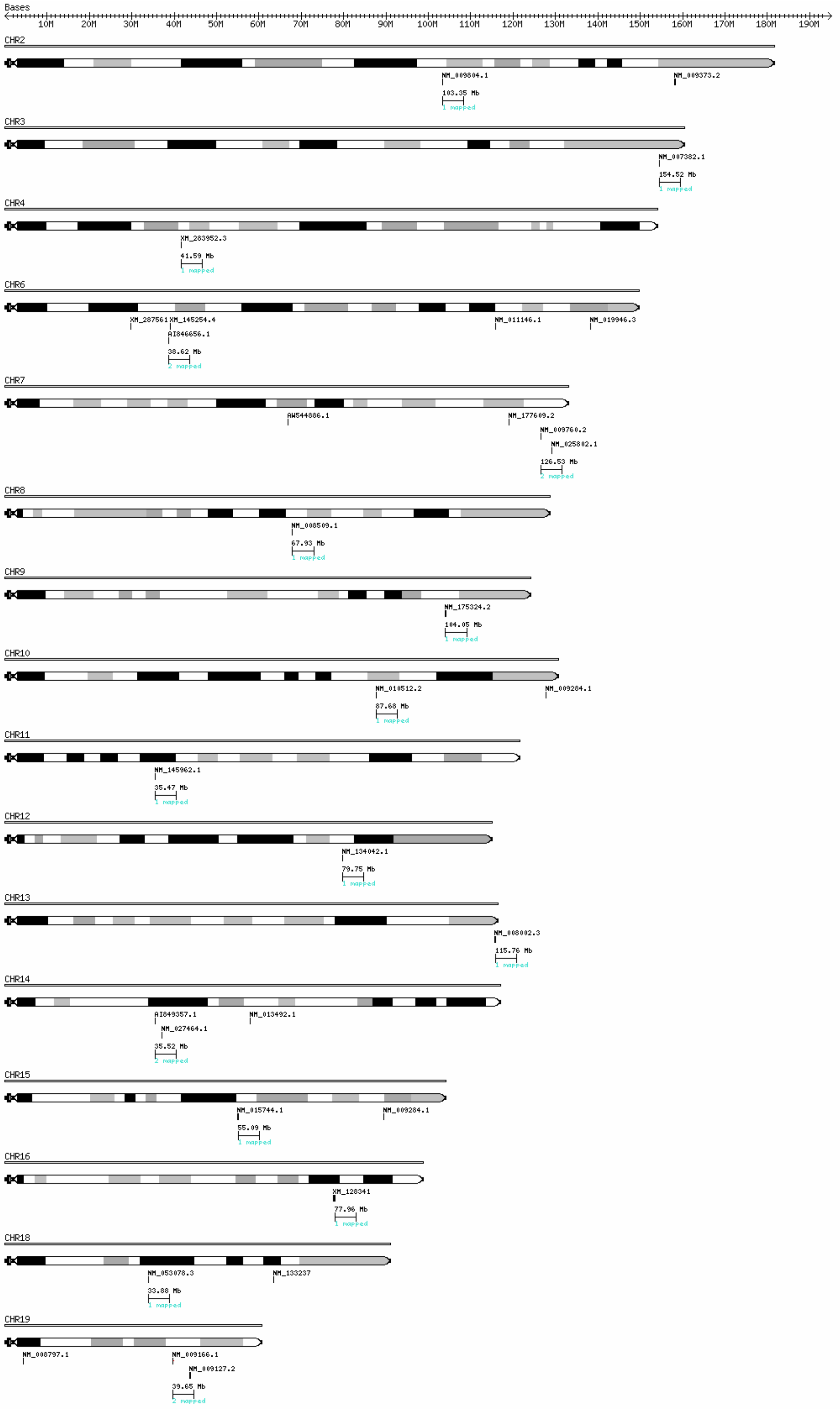

Bases
10M
20M
30M
40M
50M
60M
70M
80M
90M
100M
110M
120M
130M
140M
150M
160M
170M
180M
190M
CHR2
NM_009804.1
NM_009373.2
103.35 Mb
1 mapped
CHR3
NM_007382.1
154.52 Mb
1 mapped
CHR4
XM_283952.3
41.59 Mb
1 mapped
CHR6
XM_287561
XM_145254.4
AI846656.1
NM_011146.1
NM_019946.3
38.62 Mb
2 mapped
CHR7
AW544886.1
NM_177609.2
NM_009760.2
NM_025802.1
126.53 Mb
2 mapped
CHR8
NM_008509.1
67.93 Mb
1 mapped
CHR9
NM_175324.2
104.05 Mb
1 mapped
CHR10
NM_010512.2
NM_009284.1
87.68 Mb
1 mapped
CHR11
NM_145962.1
35.47 Mb
1 mapped
CHR12
NM_134042.1
79.75 Mb
1 mapped
CHR13
NM_008002.3
115.76 Mb
1 mapped
CHR14
AI849357.1
NM_013492.1
NM_027464.1
35.52 Mb
2 mapped
CHR15
NM_015744.1
NM_009284.1
55.09 Mb
1 mapped
CHR16
XM_128341
77.96 Mb
1 mapped
CHR18
NM_053078.3
NM_133237
33.88 Mb
1 mapped
CHR19
NM_008797.1
NM_009166.1
NM_009127.2
39.65 Mb
2 mapped

Cluster 7

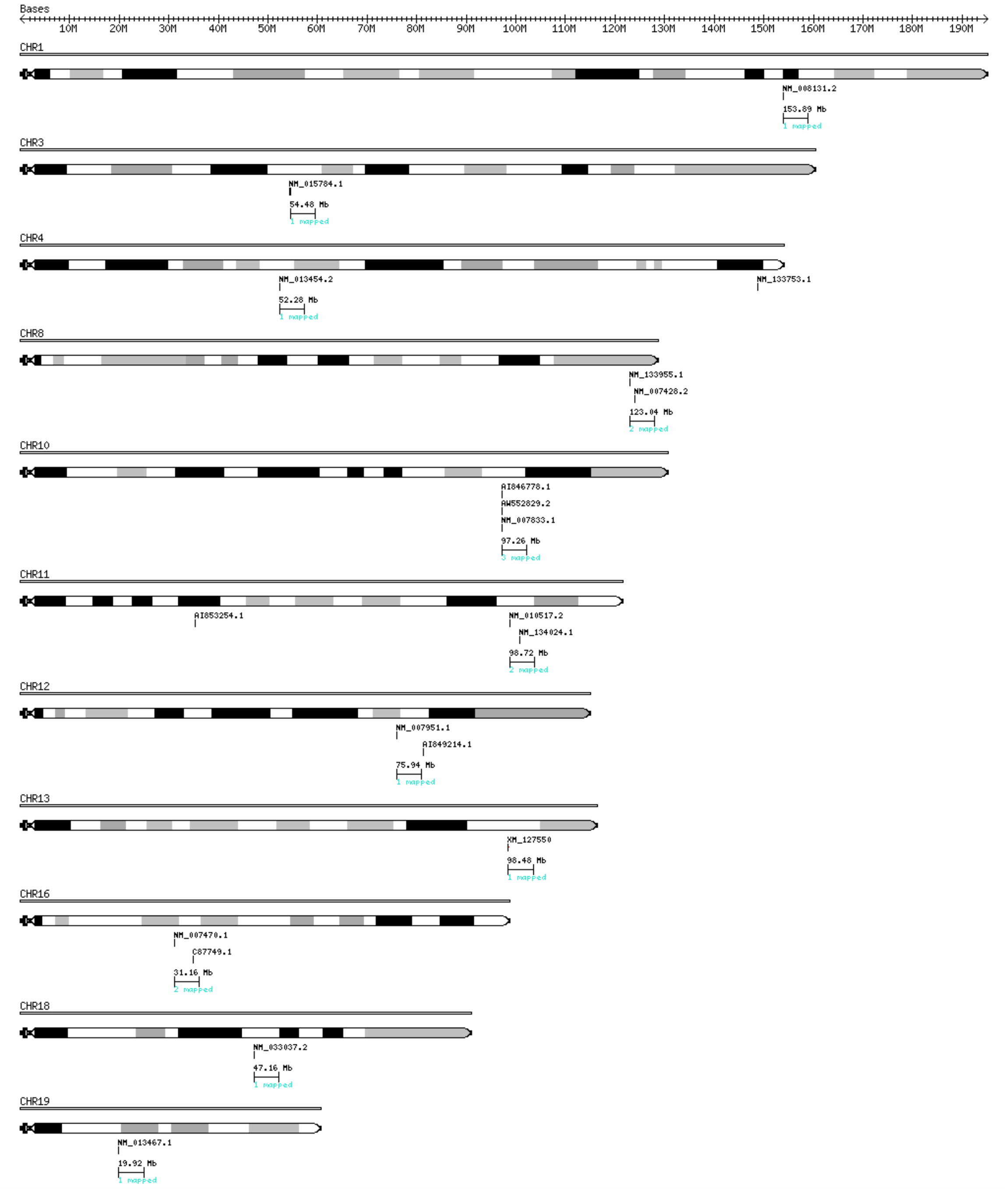
Bases
10M 20M 30M 40M 50M 60M 70M 80M 90M 100M 110M 120M 130M 140M 150M 160M 170M 180M 190M
CHR1
NM_008131.2
153.89 Mb
1 mapped
CHR3
NM_015784.1
54.48 Mb
1 mapped
CHR4
NM_013454.2
52.28 Mb
1 mapped
NM_133753.1
CHR8
NM_133955.1
NM_007428.2
123.04 Mb
2 mapped
CHR10
AI846778.1
AW552829.2
NM_007833.1
97.26 Mb
3 mapped
CHR11
AI853254.1
NM_010517.2
NM_134024.1
98.72 Mb
2 mapped
CHR12
NM_007951.1
AI849214.1
75.94 Mb
1 mapped
CHR13
XM_127550
98.48 Mb
1 mapped
CHR16
NM_007470.1
C87749.1
31.16 Mb
2 mapped
CHR18
NM_033037.2
47.16 Mb
1 mapped
CHR19
NM_013467.1
19.92 Mb
1 mapped

Cluster 8A

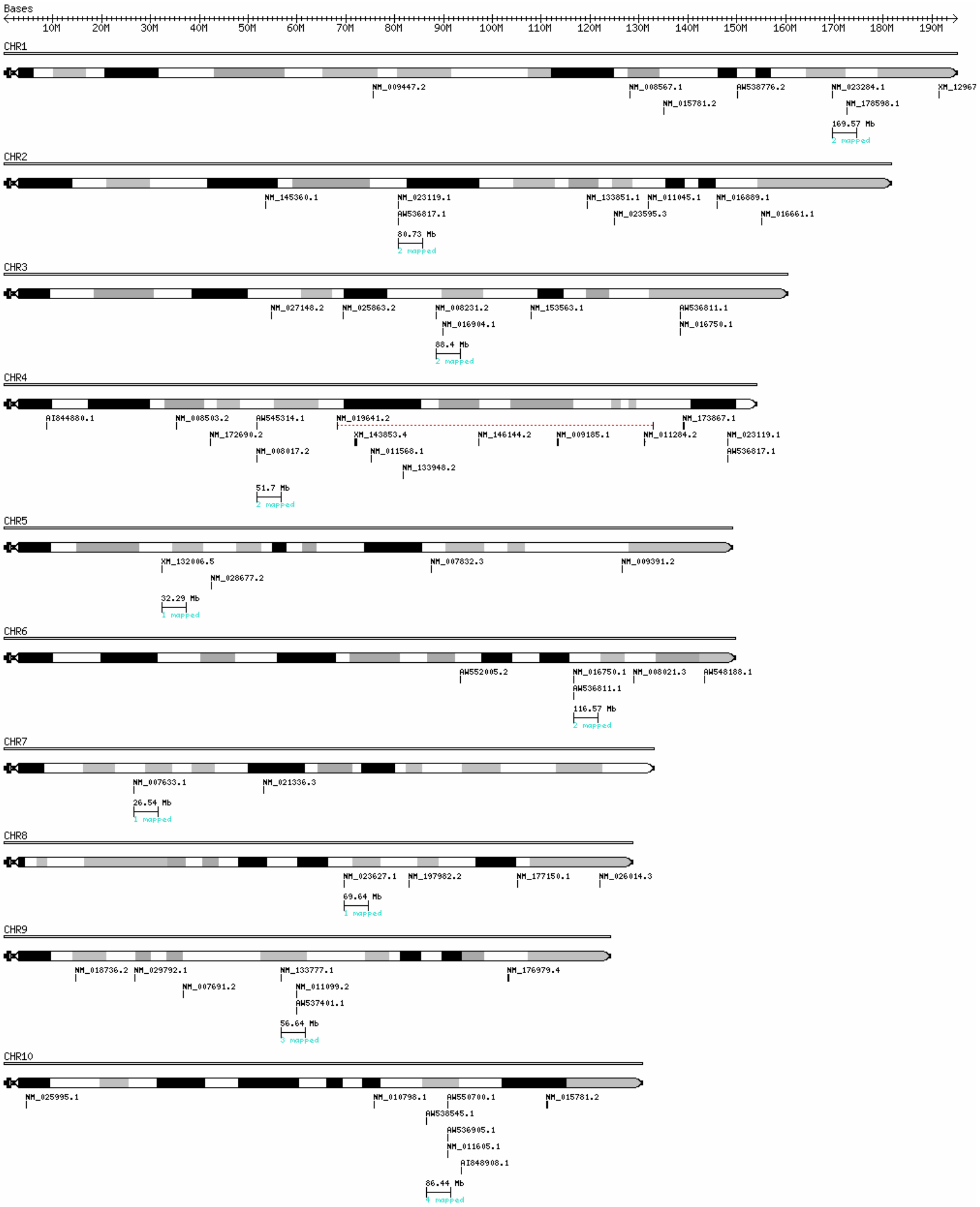

Bases
10M 20M 30M 40M 50M 60M 70M 80M 90M 100M 110M 120M 130M 140M 150M 160M 170M 180M 190M
CHR1
NM_009447.2
NM_008567.1
NM_015781.2
AW538776.2
NM_023284.1
NM_178598.1
XM_12967
169.57 Mb
2 mapped
CHR2
NM_145360.1
NM_023119.1
AW536817.1
80.73 Mb
2 mapped
NM_133851.1
NM_011045.1
NM_023595.3
NM_016889.1
NM_016661.1
CHR3
NM_027148.2
NM_025863.2
NM_008231.2
NM_016904.1
88.4 Mb
2 mapped
NM_153563.1
AW536811.1
NM_016750.1
CHR4
AI844880.1
NM_008503.2
NM_172690.2
AW545314.1
NM_008017.2
51.7 Mb
2 mapped
NM_019641.2
XM_143853.4
NM_011568.1
NM_133948.2
NM_146144.2
NM_009185.1
NM_011284.2
NM_173867.1
NM_023119.1
AW536817.1
CHR5
XM_132006.5
NM_028677.2
32.29 Mb
1 mapped
NM_007832.3
NM_009391.2
CHR6
AW552005.2
NM_016750.1
AW536811.1
116.57 Mb
2 mapped
NM_008021.3
AW548188.1
CHR7
NM_007633.1
26.54 Mb
1 mapped
NM_021336.3
CHR8
NM_023627.1
69.64 Mb
1 mapped
NM_197982.2
NM_177150.1
NM_026014.3
CHR9
NM_018736.2
NM_029792.1
NM_007691.2
NM_133777.1
NM_011099.2
AW537401.1
56.64 Mb
3 mapped
NM_176979.4
CHR10
NM_025995.1
NM_010798.1
AW538545.1
AW550700.1
AW536905.1
NM_011605.1
AI848908.1
86.44 Mb
4 mapped
NM_015781.2

Cluster 8B

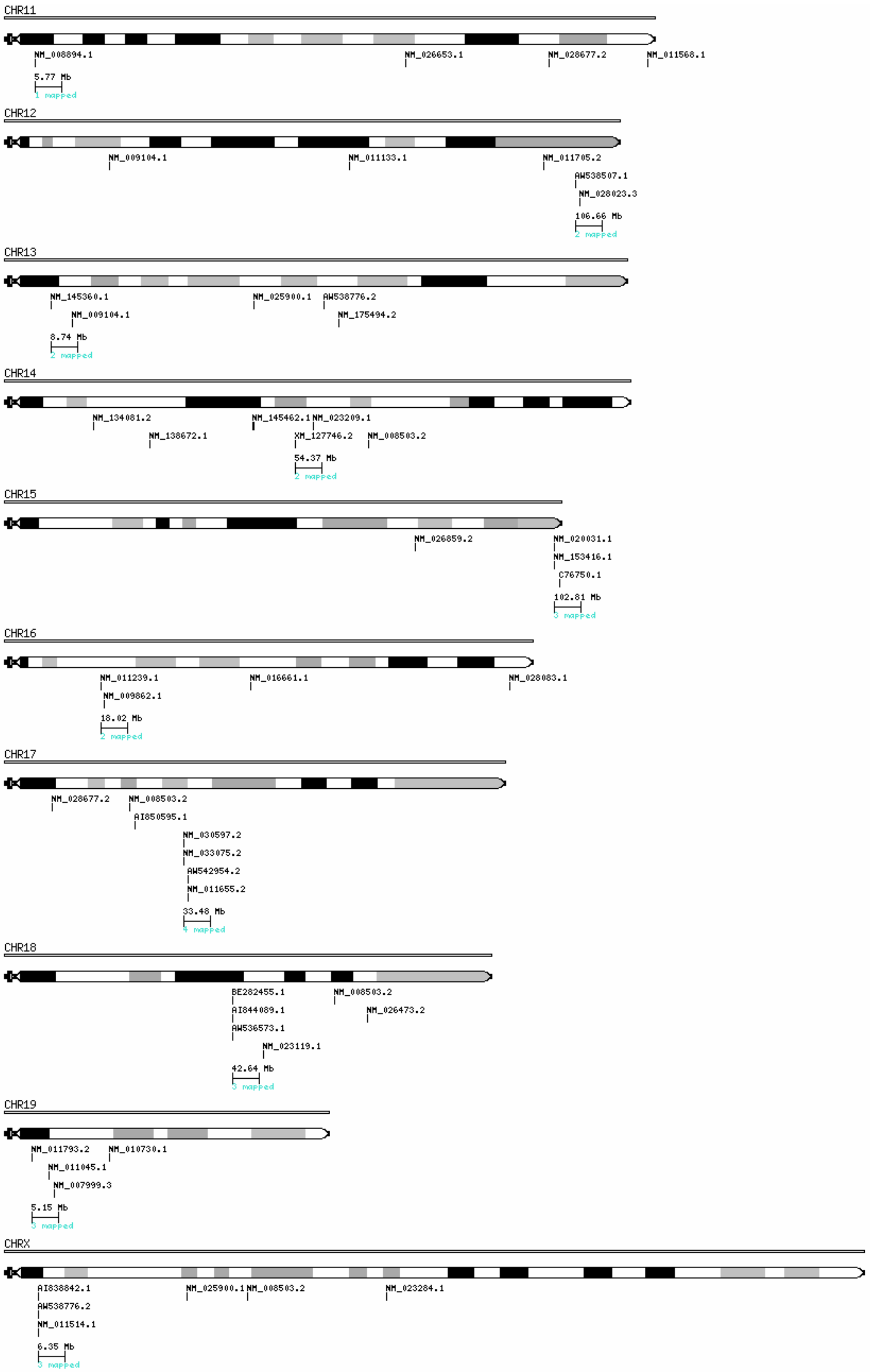
CHR11
NM_008894.1
NM_026653.1
NM_028677.2
NM_011568.1
5.77 Mb
1 mapped
CHR12
NM_009104.1
NM_011133.1
NM_011705.2
AW538507.1
NM_028023.3
106.66 Mb
2 mapped
CHR13
NM_145360.1
NM_025900.1
AW538776.2
NM_009104.1
NM_175494.2
8.74 Mb
2 mapped
CHR14
NM_134081.2
NM_145462.1
NM_023209.1
NM_138672.1
XM_127746.2
NM_008503.2
54.37 Mb
2 mapped
CHR15
NM_026859.2
NM_020031.1
NM_153416.1
C76750.1
102.81 Mb
3 mapped
CHR16
NM_011239.1
NM_016661.1
NM_028083.1
NM_009862.1
18.02 Mb
2 mapped
CHR17
NM_028677.2
NM_008503.2
AI850595.1
NM_030597.2
NM_033075.2
AW542954.2
NM_011655.2
33.48 Mb
4 mapped
CHR18
BE282455.1
NM_008503.2
AI844089.1
NM_026473.2
AW536573.1
NM_023119.1
42.64 Mb
5 mapped
CHR19
NM_011793.2
NM_010730.1
NM_011045.1
NM_007999.3
5.15 Mb
3 mapped
CHRX
AI838842.1
NM_025900.1
NM_008503.2
NM_023284.1
AW538776.2
NM_011514.1
6.35 Mb
3 mapped

Cluster 9A

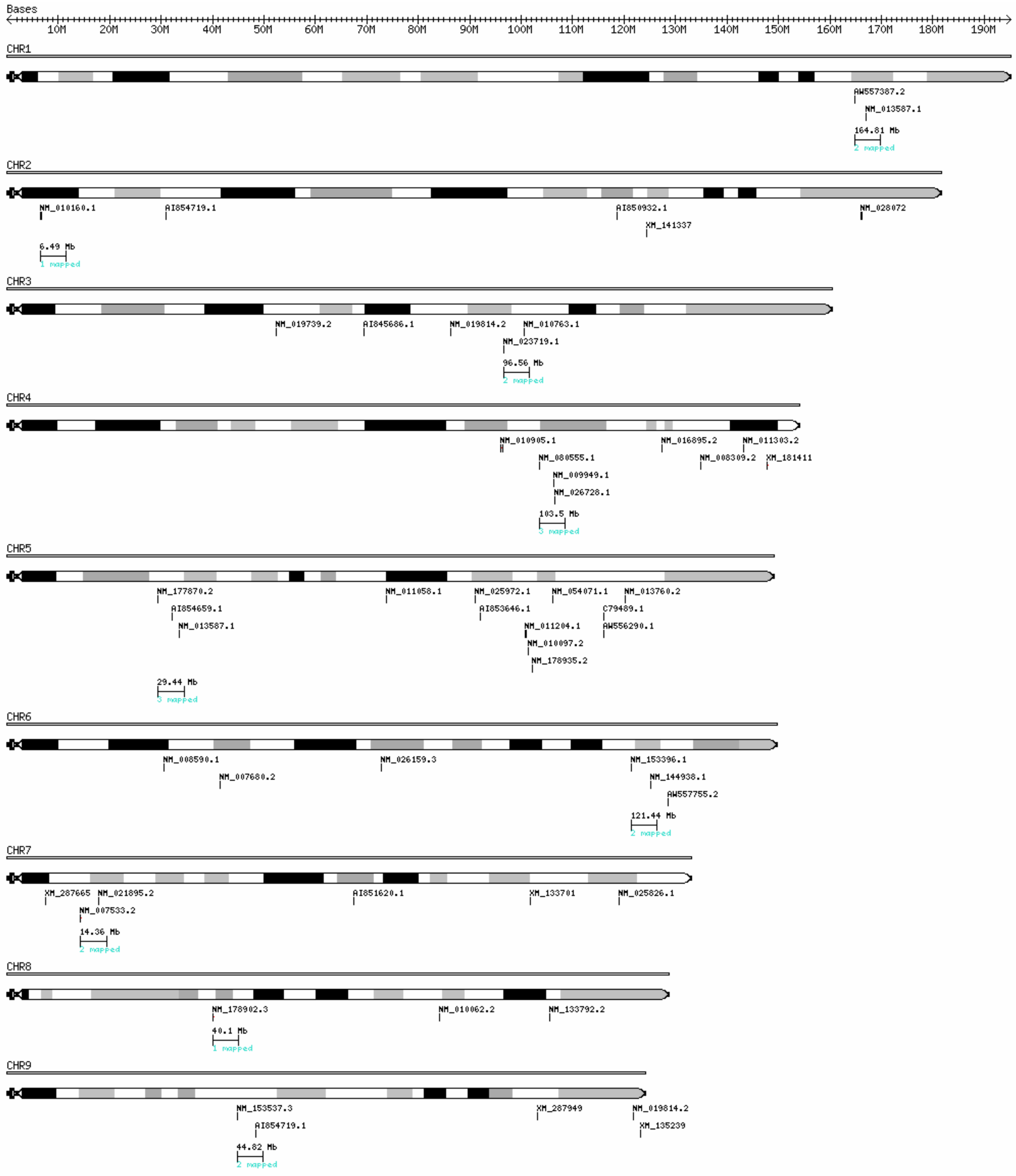
Bases
10M 20M 30M 40M 50M 60M 70M 80M 90M 100M 110M 120M 130M 140M 150M 160M 170M 180M 190M
CHR1
AW557387.2
NM_013587.1
164.81 Mb
2 mapped
CHR2
NM_010160.1
AI854719.1
AI850932.1
XM_141337
NM_028072
6.49 Mb
1 mapped
CHR3
NM_019739.2
AI845686.1
NM_019814.2
NM_010763.1
NM_023719.1
96.56 Mb
2 mapped
CHR4
NM_010905.1
NM_080555.1
NM_009949.1
NM_026728.1
NM_016895.2
NM_008309.2
NM_011303.2
XM_181411
103.5 Mb
3 mapped
CHR5
NM_177870.2
AI854659.1
NM_013587.1
NM_011058.1
NM_025972.1
AI853646.1
NM_054071.1
NM_011204.1
NM_010097.2
NM_178935.2
NM_013760.2
C79489.1
AW556290.1
29.44 Mb
3 mapped
CHR6
NM_008590.1
NM_007680.2
NM_026159.3
NM_153396.1
NM_144938.1
AW557755.2
121.44 Mb
2 mapped
CHR7
XM_287665
NM_021895.2
NM_007533.2
AI851620.1
XM_133701
NM_025826.1
14.36 Mb
2 mapped
CHR8
NM_178902.3
NM_010062.2
NM_133792.2
40.1 Mb
1 mapped
CHR9
NM_153537.3
AI854719.1
XM_287949
NM_019814.2
XM_135239
44.82 Mb
2 mapped

Cluster 9B

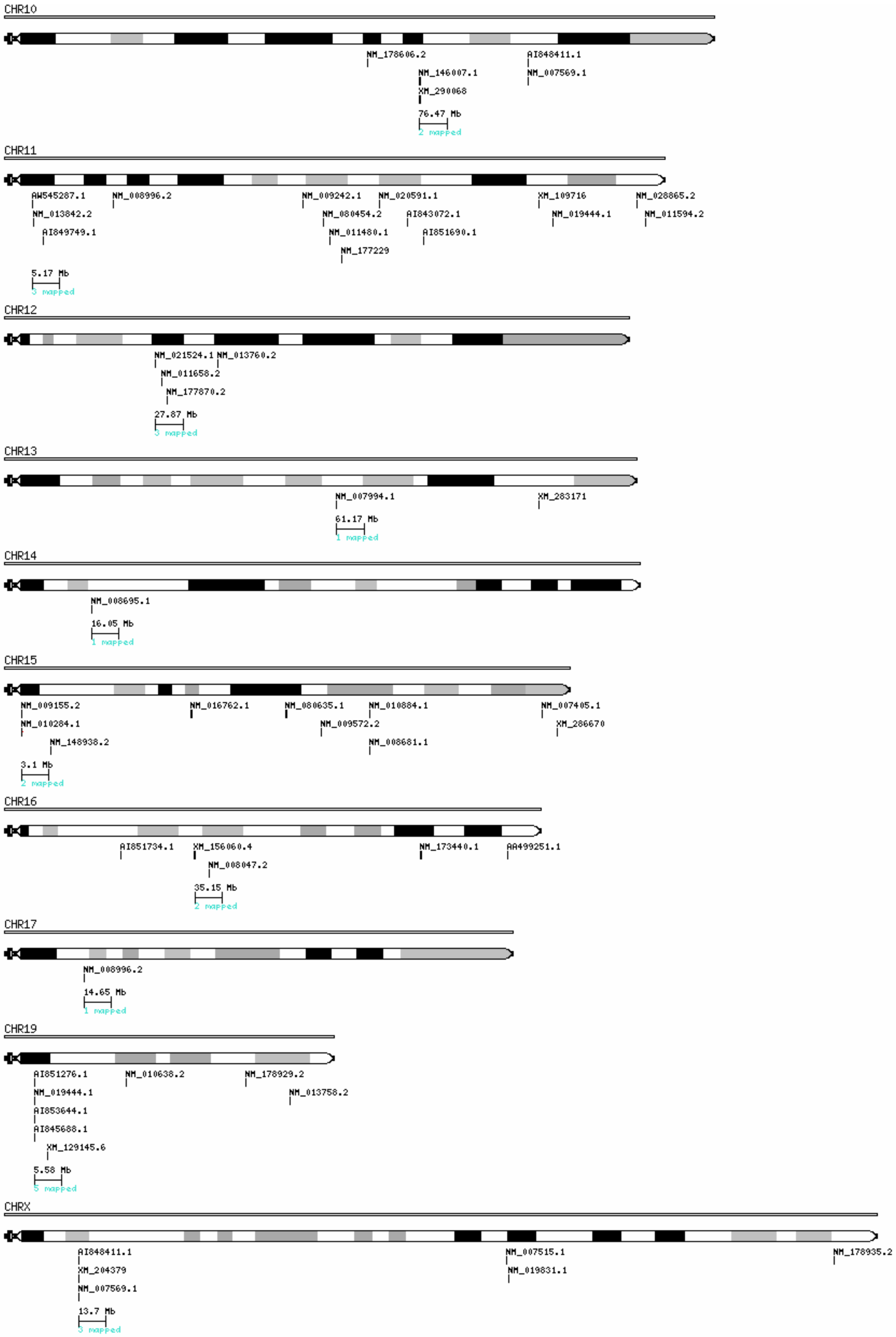

CHR10
NM_178606.2
NM_146007.1
XM_290068
76.47 Mb
2 mapped
AI848411.1
NM_007569.1
CHR11
AW545287.1
NM_013842.2
AI849749.1
NM_008996.2
NM_009242.1
NM_080454.2
NM_011480.1
NM_177229
NM_020591.1
AI843072.1
AI851690.1
XM_109716
NM_019444.1
NM_028865.2
NM_011594.2
5.17 Mb
3 mapped
CHR12
NM_021524.1
NM_013760.2
NM_011658.2
NM_177870.2
27.87 Mb
3 mapped
CHR13
NM_007994.1
XM_283171
61.17 Mb
1 mapped
CHR14
NM_008695.1
16.05 Mb
1 mapped
CHR15
NM_009155.2
NM_010284.1
NM_148938.2
NM_016762.1
NM_080635.1
NM_009572.2
NM_010884.1
NM_008681.1
NM_007405.1
XM_286670
3.1 Mb
2 mapped
CHR16
AI851734.1
XM_156060.4
NM_008047.2
NM_173440.1
AA499251.1
35.15 Mb
2 mapped
CHR17
NM_008996.2
14.65 Mb
1 mapped
CHR19
AI851276.1
NM_019444.1
AI853644.1
AI845688.1
XM_129145.6
NM_010638.2
NM_178929.2
NM_013758.2
5.58 Mb
5 mapped
CHRX
AI848411.1
XM_204379
NM_007569.1
13.7 Mb
3 mapped
NM_007515.1
NM_019831.1
NM_178935.2

Cluster 10A

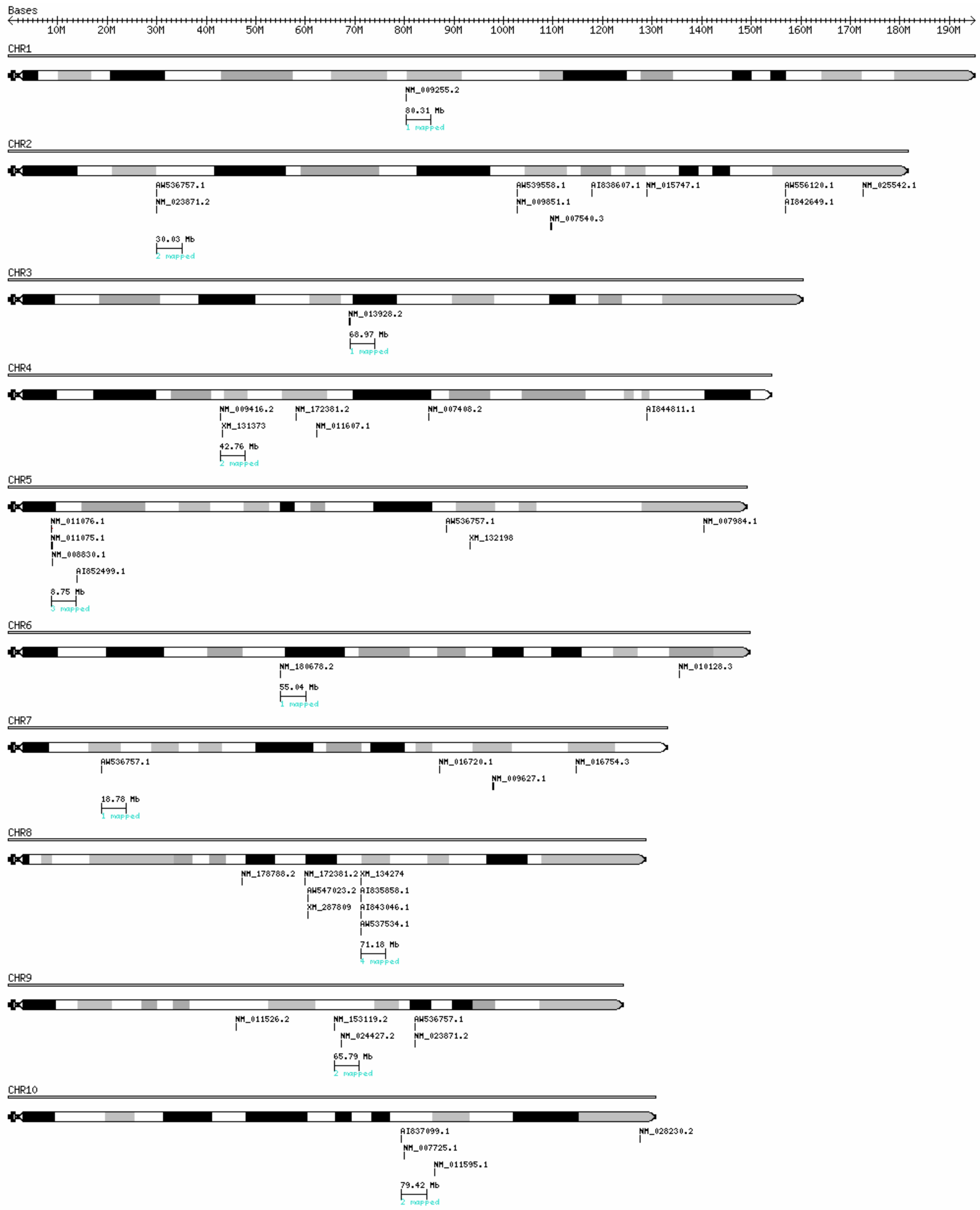
Bases
10M 20M 30M 40M 50M 60M 70M 80M 90M 100M 110M 120M 130M 140M 150M 160M 170M 180M 190M
CHR1
NM_009255.2
80.31 Mb
1 mapped
CHR2
AW536757.1
NM_023871.2
AW539558.1
NM_009851.1
NM_007540.3
AI838607.1
NM_015747.1
AW556120.1
AI842649.1
NM_025542.1
30.03 Mb
2 mapped
CHR3
NM_013928.2
68.97 Mb
1 mapped
CHR4
NM_009416.2
XM_131373
NM_172381.2
NM_011607.1
NM_007408.2
AI844811.1
42.76 Mb
2 mapped
CHR5
NM_011076.1
NM_011075.1
NM_008830.1
AI852499.1
AW536757.1
XM_132198
NM_007984.1
8.75 Mb
3 mapped
CHR6
NM_180678.2
NM_010128.3
55.04 Mb
1 mapped
CHR7
AW536757.1
NM_016720.1
NM_009627.1
NM_016754.3
18.78 Mb
1 mapped
CHR8
NM_178788.2
NM_172381.2
AW547023.2
XM_287809
XM_134274
AI835858.1
AI843046.1
AW537534.1
71.18 Mb
4 mapped
CHR9
NM_011526.2
NM_153119.2
NM_024427.2
AW536757.1
NM_023871.2
65.79 Mb
2 mapped
CHR10
AI837099.1
NM_007725.1
NM_011595.1
NM_028230.2
79.42 Mb
2 mapped

Cluster 10B

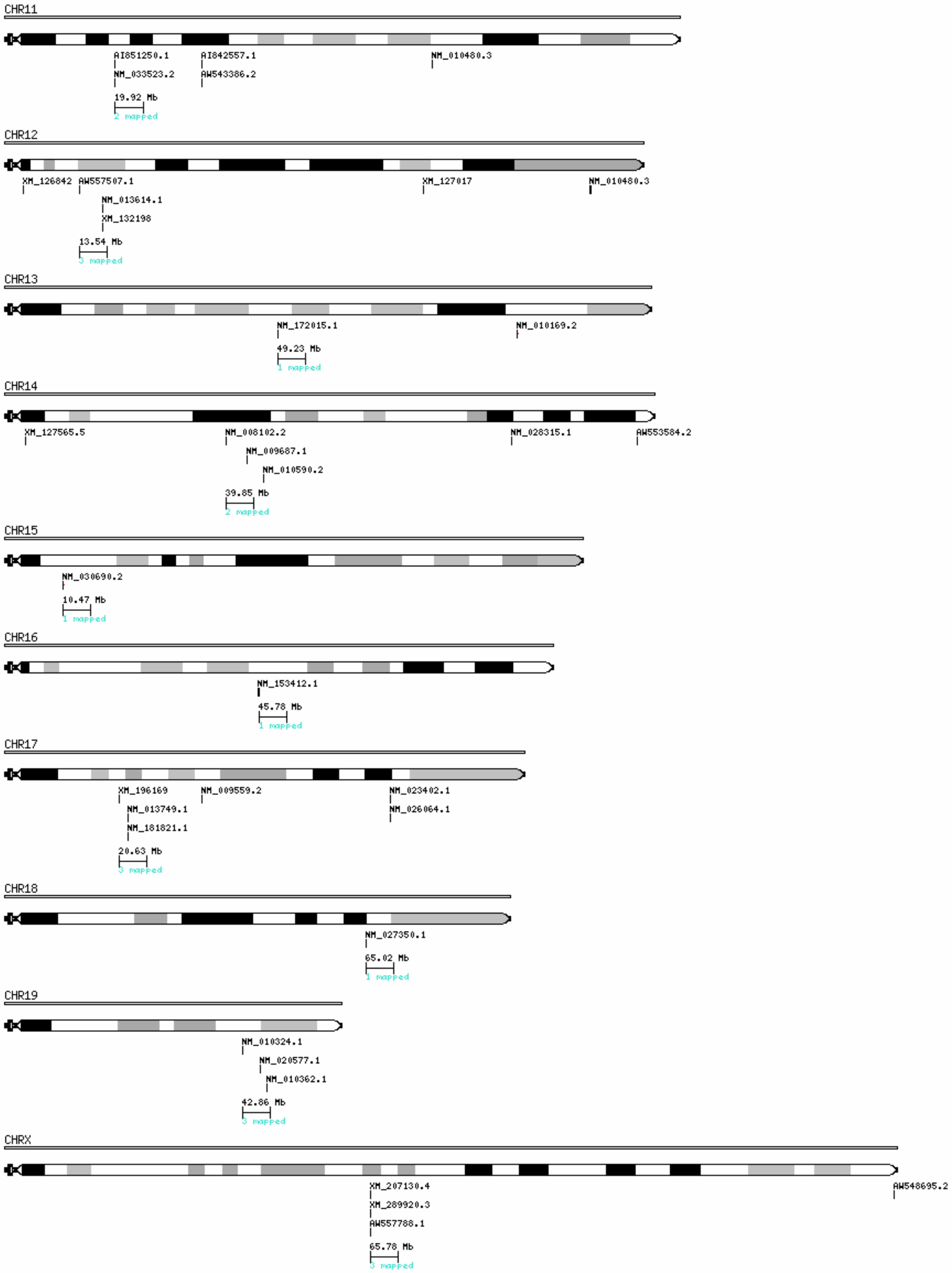
CHR11
AI851250.1
NM_033523.2
AI842557.1
AW543386.2
NM_010480.3
19.92 Mb
2 mapped
CHR12
XM_126842
AW557507.1
NM_013614.1
XM_132198
XM_127017
NM_010480.3
13.54 Mb
3 mapped
CHR13
NM_172015.1
NM_010169.2
49.23 Mb
1 mapped
CHR14
XM_127565.5
NM_008102.2
NM_009687.1
NM_010590.2
NM_028315.1
AW553584.2
39.85 Mb
2 mapped
CHR15
NM_030690.2
10.47 Mb
1 mapped
CHR16
NM_153412.1
45.78 Mb
1 mapped
CHR17
XM_196169
NM_013749.1
NM_181821.1
NM_009559.2
NM_023402.1
NM_026064.1
20.63 Mb
3 mapped
CHR18
NM_027350.1
65.02 Mb
1 mapped
CHR19
NM_010324.1
NM_020577.1
NM_010362.1
42.86 Mb
3 mapped
CHRX
XM_207130.4
XM_289920.3
AW557788.1
AW548695.2
65.78 Mb
3 mapped

Cluster 11

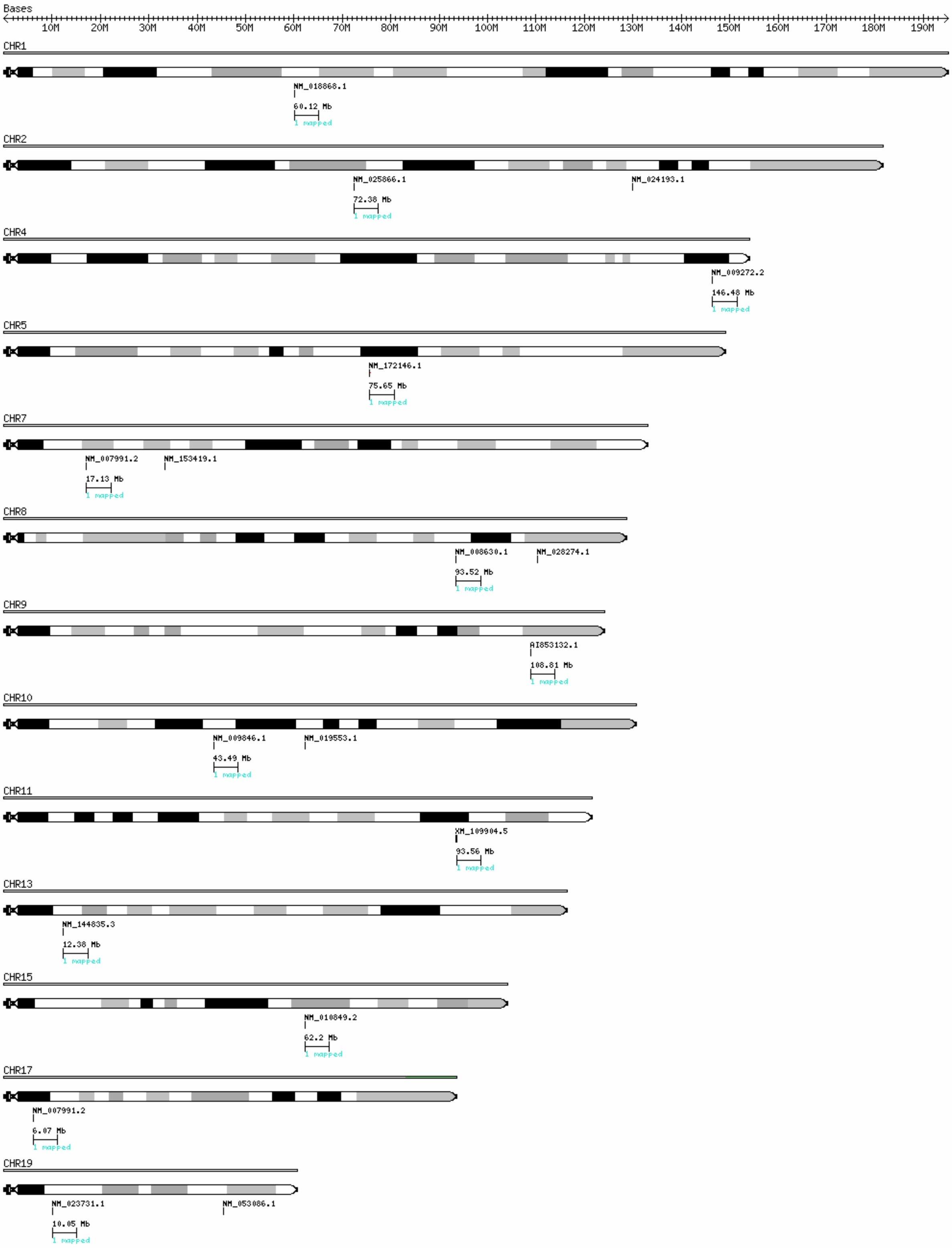

Bases
10M 20M 30M 40M 50M 60M 70M 80M 90M 100M 110M 120M 130M 140M 150M 160M 170M 180M 190M
CHR1
NM_018868.1
60.12 Mb
1 mapped
CHR2
NM_025866.1
NM_024193.1
72.38 Mb
1 mapped
CHR4
NM_009272.2
146.48 Mb
1 mapped
CHR5
NM_172146.1
75.65 Mb
1 mapped
CHR7
NM_007991.2
NM_153419.1
17.13 Mb
1 mapped
CHR8
NM_008630.1
NM_028274.1
93.52 Mb
1 mapped
CHR9
AI853132.1
108.81 Mb
1 mapped
CHR10
NM_009846.1
NM_019553.1
43.49 Mb
1 mapped
CHR11
XM_109904.5
93.56 Mb
1 mapped
CHR13
NM_144835.3
12.38 Mb
1 mapped
CHR15
NM_010849.2
62.2 Mb
1 mapped
CHR17
NM_007991.2
6.07 Mb
1 mapped
CHR19
NM_023731.1
NM_053086.1
10.05 Mb
1 mapped

Cluster 12A

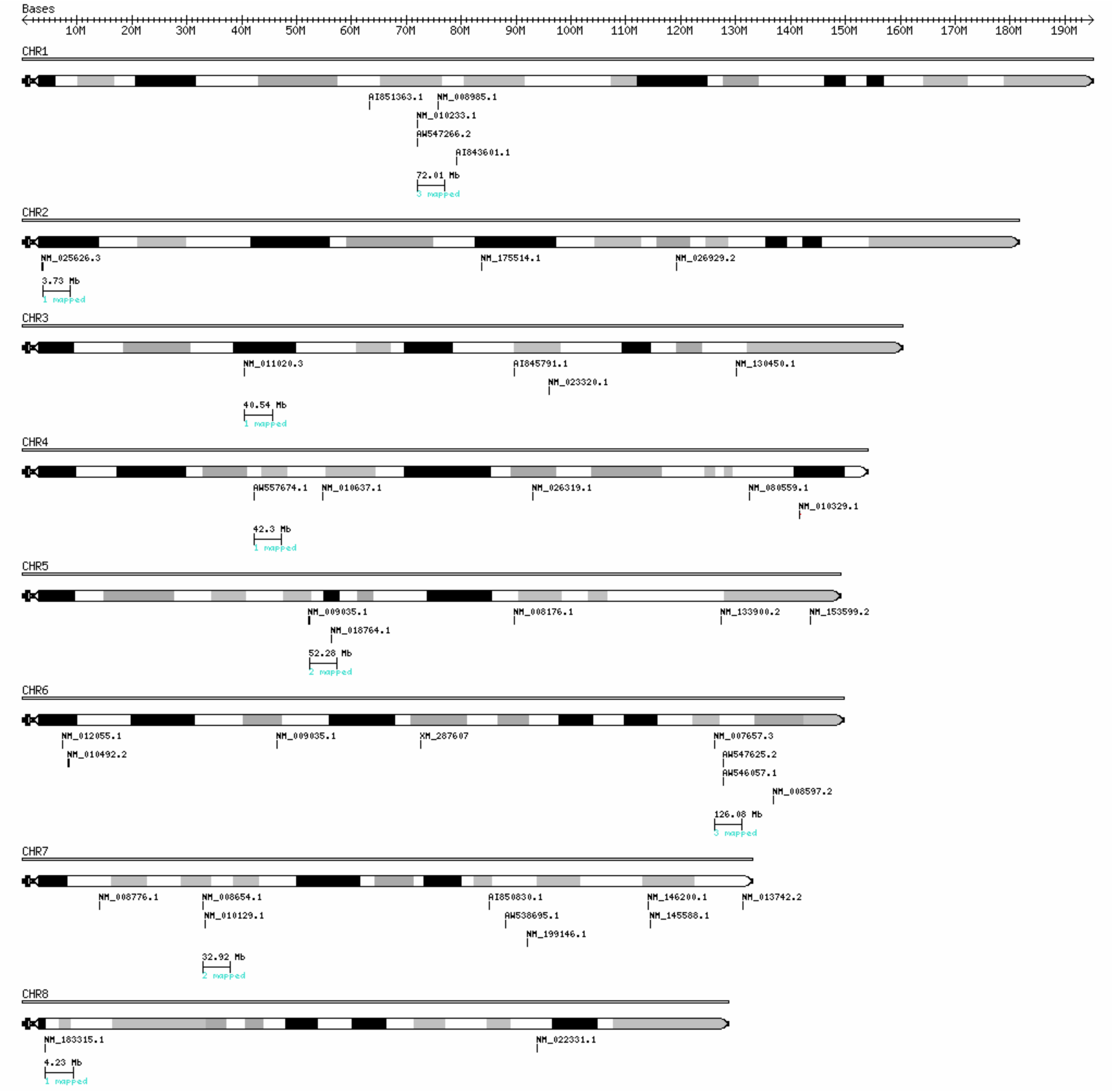
Bases
10M 20M 30M 40M 50M 60M 70M 80M 90M 100M 110M 120M 130M 140M 150M 160M 170M 180M 190M
CHR1
AI851363.1
NM_008985.1
NM_010233.1
AW547266.2
AI843601.1
72.01 Mb
3 mapped
CHR2
NM_025626.3
NM_175514.1
NM_026929.2
3.73 Mb
1 mapped
CHR3
NM_011020.3
AI845791.1
NM_130450.1
NM_023320.1
40.54 Mb
1 mapped
CHR4
AW557674.1
NM_010637.1
NM_026319.1
NM_080559.1
NM_010329.1
42.3 Mb
1 mapped
CHR5
NM_009035.1
NM_008176.1
NM_133900.2
NM_153599.2
NM_018764.1
52.28 Mb
2 mapped
CHR6
NM_012055.1
NM_009035.1
XM_287607
NM_007657.3
NM_010492.2
AW547625.2
AW546057.1
NM_008597.2
126.08 Mb
3 mapped
CHR7
NM_008776.1
NM_008654.1
AI850830.1
NM_146200.1
NM_013742.2
NM_010129.1
AW538695.1
NM_145588.1
NM_199146.1
32.92 Mb
2 mapped
CHR8
NM_183315.1
NM_022331.1
4.23 Mb
1 mapped

Cluster 12B

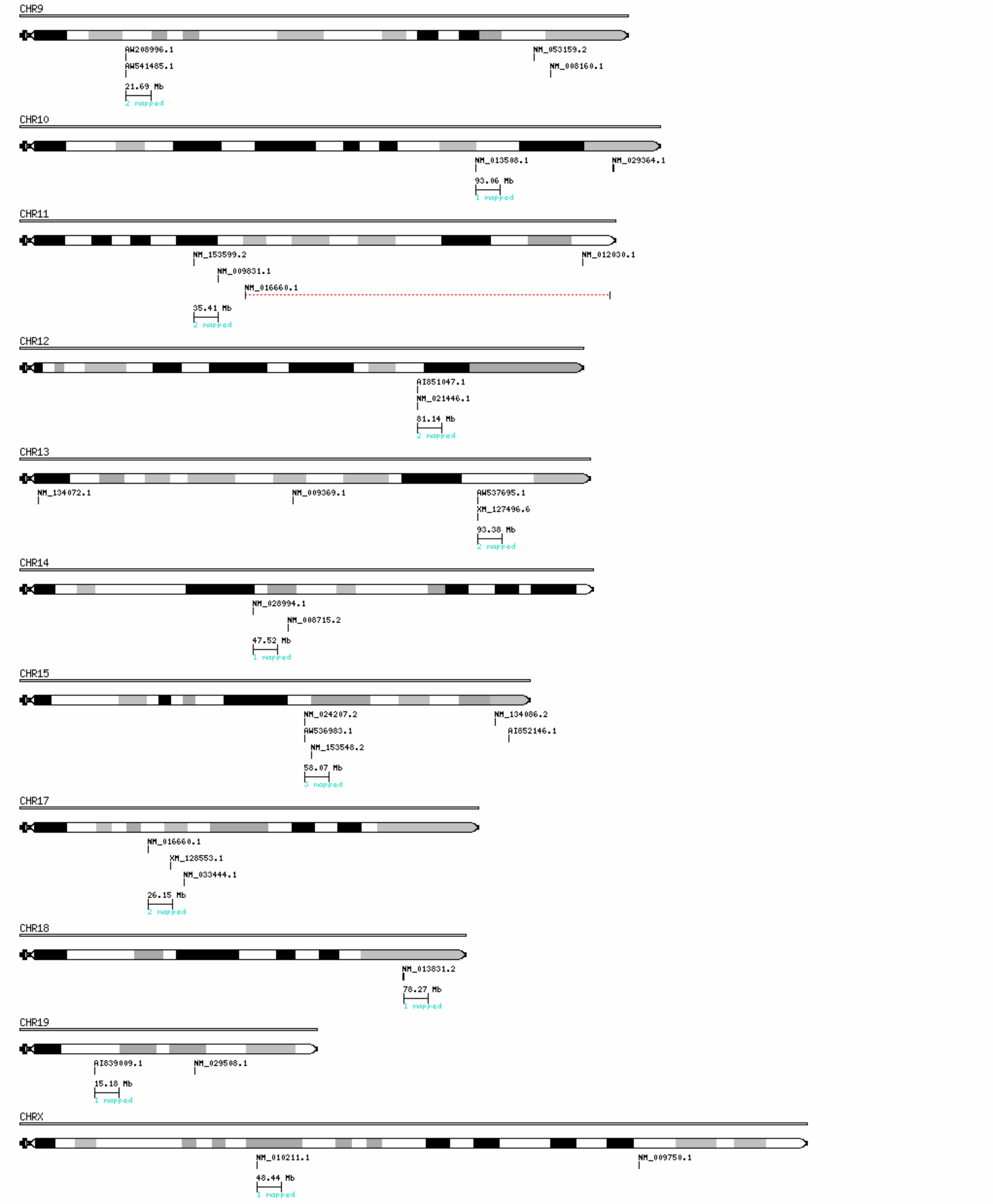
CHR9
AW208996.1
AW541485.1
21.69 Mb
2 mapped
NM_053159.2
NM_008160.1
CHR10
NM_013508.1
NM_029364.1
93.06 Mb
1 mapped
CHR11
NM_153599.2
NM_009831.1
NM_016660.1
35.41 Mb
2 mapped
NM_012030.1
CHR12
AI851047.1
NM_021446.1
81.14 Mb
2 mapped
CHR13
NM_134072.1
NM_009369.1
AW537695.1
XM_127496.6
93.38 Mb
2 mapped
CHR14
NM_028994.1
NM_008715.2
47.52 Mb
1 mapped
CHR15
NM_024207.2
AW536983.1
NM_153548.2
58.07 Mb
3 mapped
NM_134086.2
AI852146.1
CHR17
NM_016660.1
XM_128553.1
NM_033444.1
26.15 Mb
2 mapped
CHR18
NM_013831.2
78.27 Mb
1 mapped
CHR19
AI839009.1
15.18 Mb
1 mapped
NM_029508.1
CHRX
NM_010211.1
48.44 Mb
1 mapped
NM_009750.1
