# Supplementary material for: Molecular processes during fat cell development revealed by gene expression profiling and functional annotation
Source: Genome Biol. 2005 Dec 19;6(13):R108. doi: 10.1186/gb-2005-6-13-r108 (PMC1414107; doi:10.1186/gb-2005-6-13-r108)
Supplement: Additional data file 43 — A figure showing clusterwise mapping of 780 ESTs to all chromosomes [file gb-2005-6-13-r108-S43.pdf]

Chromosome 1

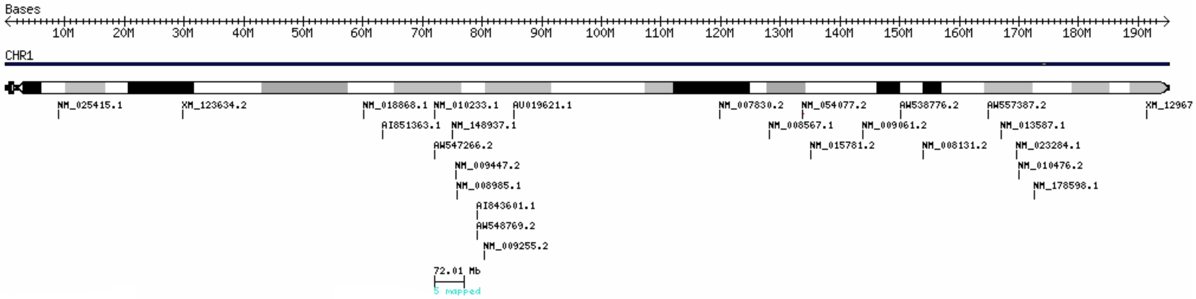

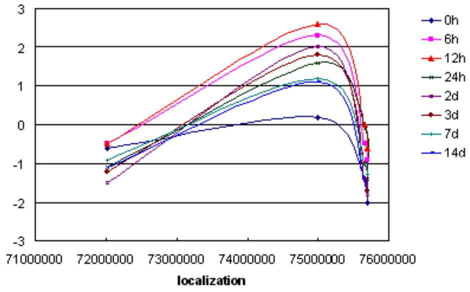

chr	start	end	cluster	RefSeq/AccNr	Name
1	72008933	75660599	12	AW547266	Fibronectin
1	75003019	75023850	2	NM_148937	Phospholipase C, delta 4
1	75659986	75660599	8	NM_009447	Tubulin alpha-1 chain
1	75691210	75708690	12	NM_008985	Protein tyrosine phosphatase, receptor-type, N

Chromosome 2

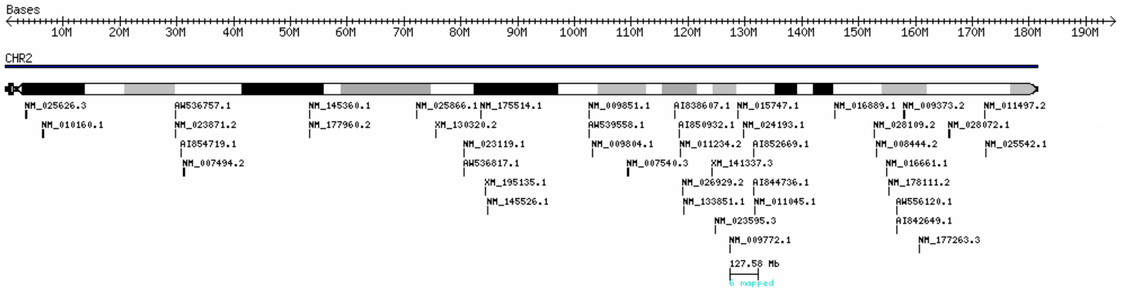

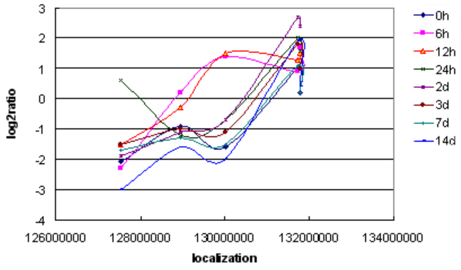

chr	start	end	cluster	RefSeq/AccNr	Name
2	127550463	127578460	5	NM_009772	Mitotic checkpoint serine/threonine-protein kinase BUB1
2	128947823	128960632	10	NM_015747	Solute carrier family 20, member 1
2	130024052	130028335	11	NM_024193	Nuclear protein Nop 56
2	131741950	8383576	2	AI852669	null
2	131801519	130028335	2	AI844736	Solute carrier family 23, member 1

Chromsome 3

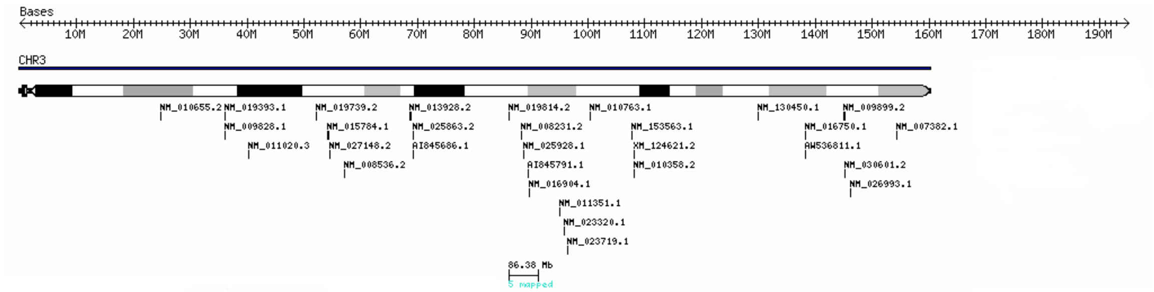

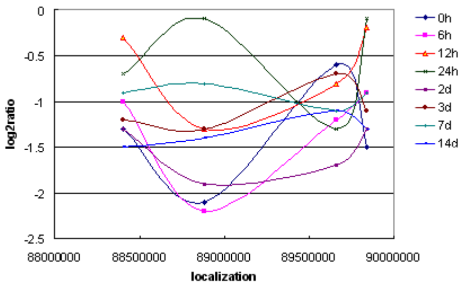

chr	start	end	cluster	RefSeq/AccNr	Name
3	88402897	88405710	8	NM_008231	Hepatoma-derived growth factor
3	88884361	88899944	5	NM_025928	Polyamine modulated factor-1
3	89658743	88405710	12	AI845791	null
3	89840946	89843133	8	NM_016904	Cyclin-dependent kinases regulatory subunit

Chromsome 4

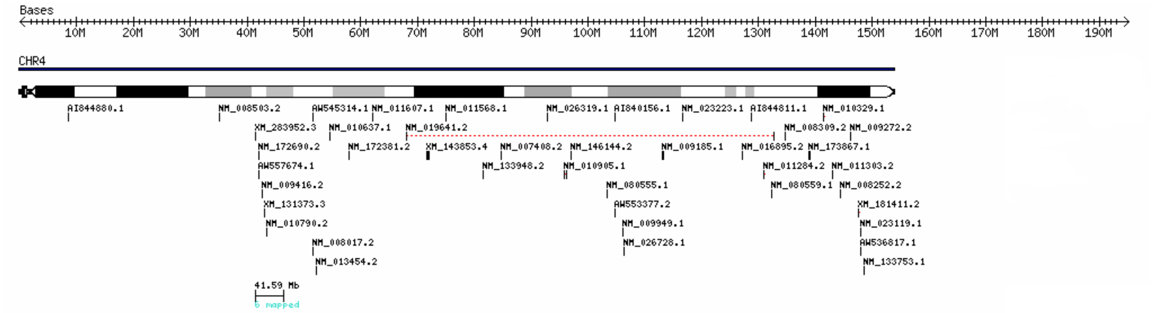

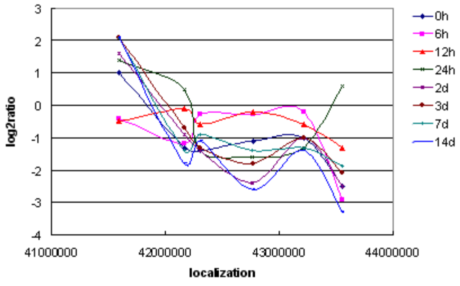

chr	start	end	cluster	RefSeq/AccNr	Name
4	41587607	41588439	6	XM_283952	null
4	42162344	42186700	8	NM_172690	Similar to HIRIP3 protein
4	42300455	43224209	12	AW557674	null
4	42761098	42768334	10	NM_009416	Tropomyosin 2, beta
4	43213202	43224209	10	XM_131373	null
4	43546010	43609768	5	NM_010790	Maternal embryonic leucine zipper kinase; protein kinase PK3

Chromosome 5

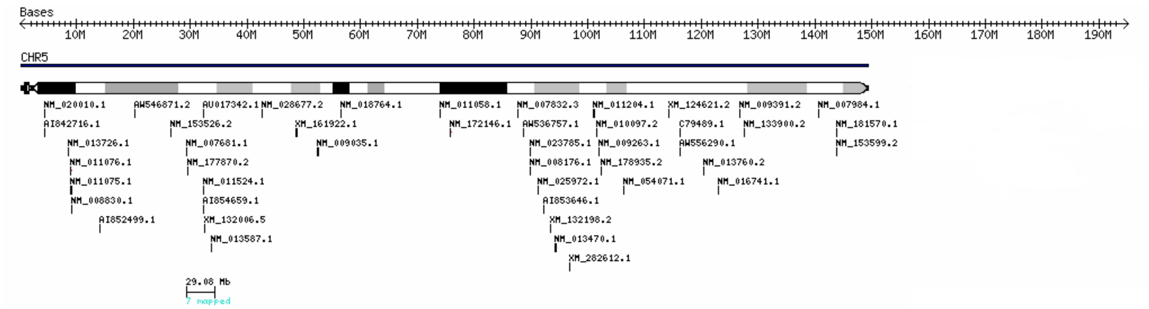

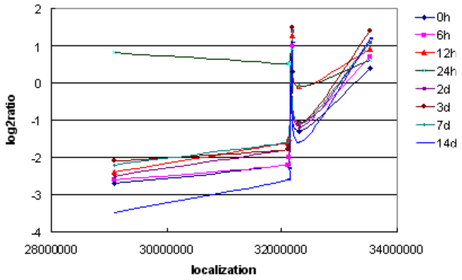

chr	start	end	cluster	RefSeq/AccNr	Name
5	29075085	29082965	5	NM_007681	Centromere protein A; centrosomin A
5	32109566	32120441	5	NM_011524	Transforming acidic coiled-coil containing protein 3
5	32127221	33550594	5	AU017342	null
5	32185531	29082965	9	AI854659	Fibroblast growth factor receptor 3 (FGFR-3)
5	32291162	32343351	8	XM_132006	Nuclear receptor-binding SET-domain protein 1 (NSD-1)
5	33538503	33550594	9	NM_013587	Low density lipoprotein receptor-related protein-associated

Chromosome 6

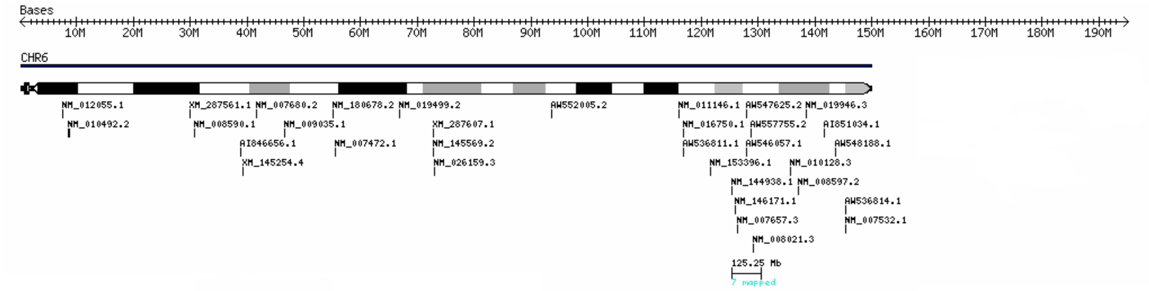

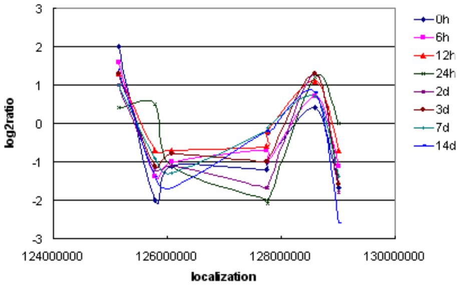

chr	start	end	cluster	RefSeq/AccNr	Name
6	125148881	125253183	9	NM_144938	Complement component 1, s subcomponent
6	125785244	125807071	8	NM_146171	Chromosome condensation-related SMC-associated protein 1
6	126076504	126111167	12	NM_007657	CD9 antigen
6	127756005	127756225	12	AW546057	null
6	127759824	127760179	12	AW547625	RAB2, member RAS oncogene family; GTP-binding protein
6	128611213	127760179	9	AW557755	null
6	129020597	129030829	8	NM_008021	Forkhead box protein M1

Chromosome 7

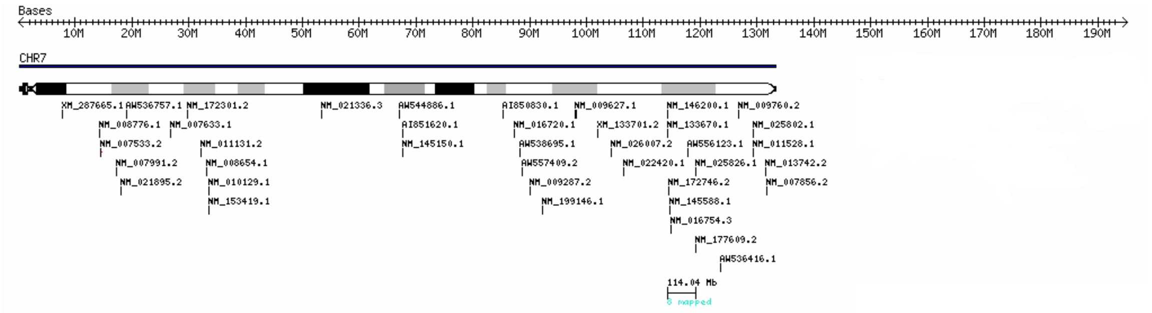

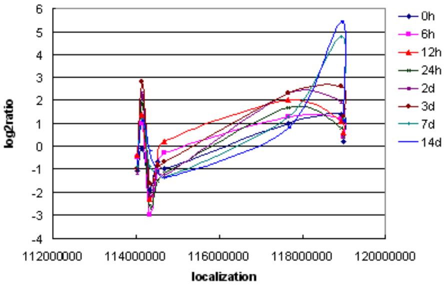

chr	start	end	cluster	RefSeq/AccNi	Name
7	114021798	114040794	12	NM_146200	Similar to eukaryotic translation initiation factor 3
7	114147970	114163271	2	NM_133670	Aryl sulfotransferase
7	114333053	114336203	4	NM_172746	Sterol-C5-desaturase
7	114498923	114506827	12	NM_145588	N-myc downstream regulated 4
7	114682689	114685368	10	NM_016754	Myosin regulatory light chain
7	117666222	114506827	2	AW556123	Fibroblast growth factor receptor
7	118942650	118959829	1	NM_177609	Similar to hypothetical protein MGC35392
7	118997590	119019489	9	NM_025826	Acyl-Coenzyme A dehydrogenase, short/branched chain

Chromosome 8

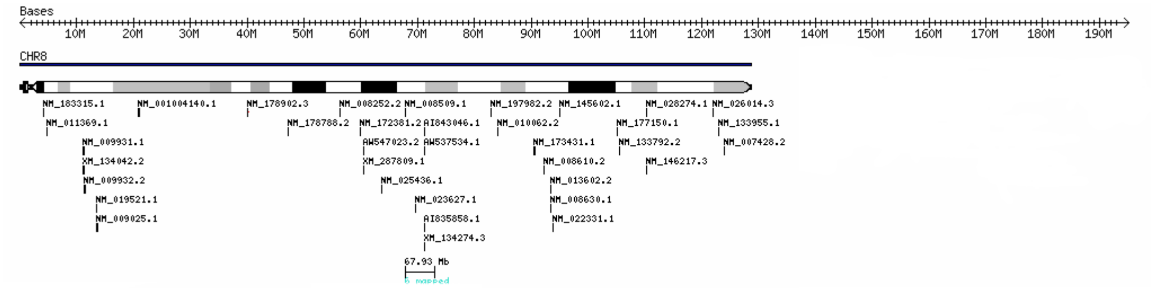

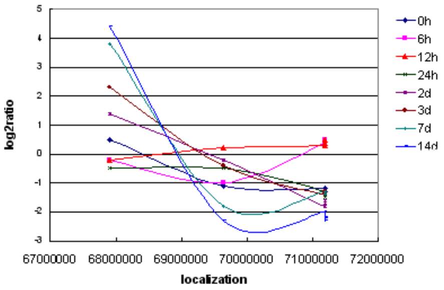

chr	start	end	cluster	RefSeq/AccNi	Name
8	67906773	67933016	6	NM_008509	Lipoptotein lipase
8	69634421	69636837	8	NM_023627	Myo-inositol 1-phosphate synthase A1
8	71176908	71185198	10	AI843046	Tropomyosin 3
8	71176935	71194769	10	XM_134274	Tropomyosin 3
8	71194761	71185198	10	AI835858	Tropomyosin 3
8	71194763	71194769	10	AW537534	Tropomyosin 3

Chromosome 9

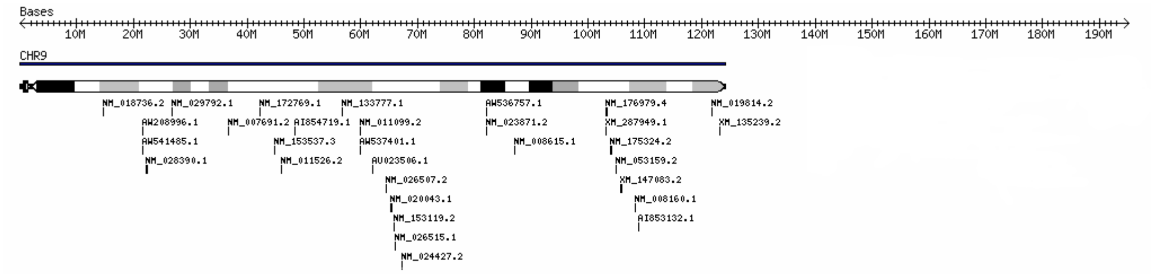

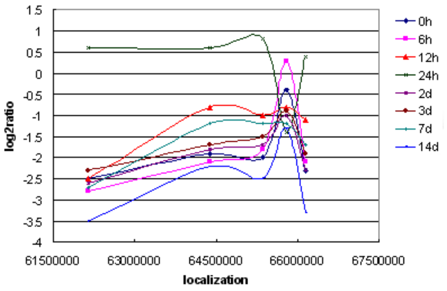

chr	start	end	cluster	RefSeq/AccNu	Name
9	62140376	65376804	5	AU023506	Kinesin family member 23
9	64374865	64402697	5	NM_026507	null
9	65353018	65376804	5	NM_020043	DDM36
9	65796852	65812746	10	NM_153119	Similar to hypothetical protein PP1628
9	66131196	66144184	5	NM_026515	Homolog to hypothetical protein KIAA0101
9	86383617	121856556	9	NM_019814	Hypoxia induced gene 1

Chromosome 10

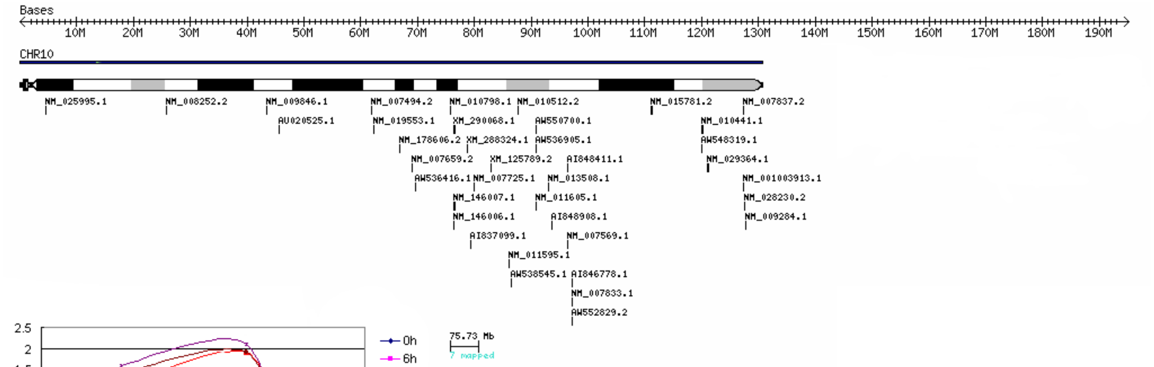

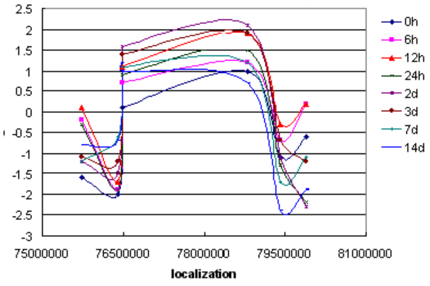

chr	start	end	cluster	RefSeq/AccNu	Name
10	75730640	75730839	8	NM_010798	Macrophage migration inhibitory factor
10	76402870	76424744	4	NM_146006	Lanosterol cyclase; similar to 2,3-oxidosqualene
10	76468310	76475795	9	XM_290068	Collagen alpha 2 (VI) chain
10	76468310	76486786	9	NM_146007	Procollagen, type VI, alpha 2
10	78803510	78845267	2	XM_288324	Similar to olfactory receptor
10	79421061	79421471	10	AI837099	Phosphatidic acid phosphatase type 2c
10	79885176	79891868	10	NM_007725	Calponin H1, smooth muscle

Chromosome 11

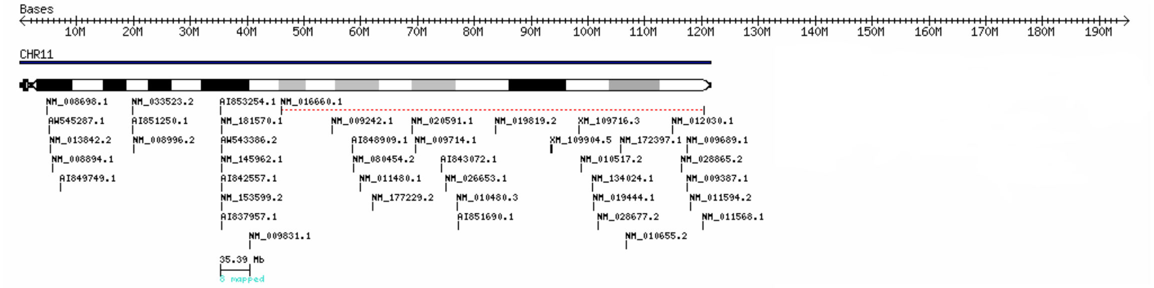

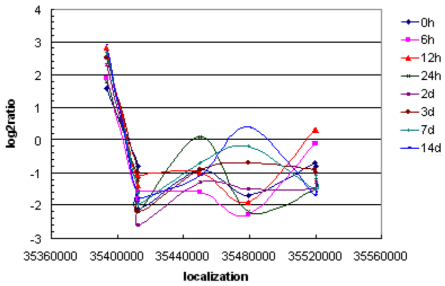

chr	start	end	cluster	RefSeq/AccN	Name
11	35392958	35412214	7	AI853254	ESTs
11	35411975	35412214	12	NM_153599	Hypothetical protein A230025O18
11	35411975	35412214	1	NM_181570	Cyclin-dependent kinase 8
11	35450412	35467437	8	NM_145962	CDK5 activator-binding protein
11	35479348	35412214	4	AI837957	Fibrillarin
11	35519701	35521341	10	AW543386	Similar to BAI1-associated protein 1; WW domain-containing
11	35519933	35521341	10	AI842557	Similar to BAI1-associated protein 1; WW domain-containing

Chromosome 12

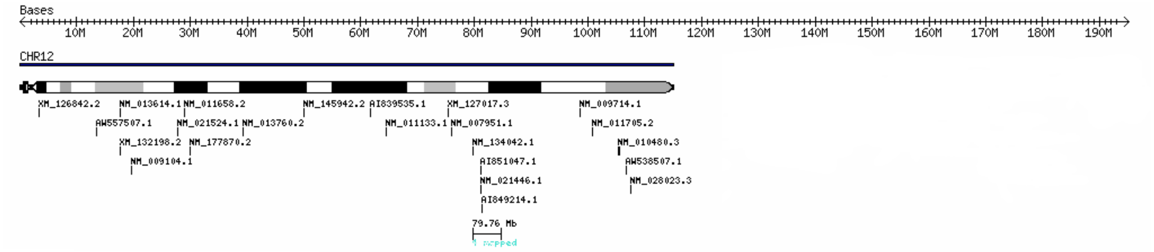

Chromosome 12

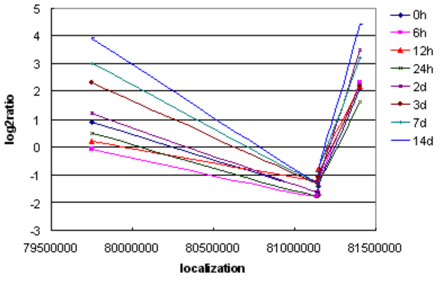

chr	start	end	cluster	RefSeq/AccNr	Name
12	79753005	79759868	6	NM_134042	Methylmalonate-semialdehyde dehydrogenase
12	81140598	81146582	12	NM_021446	null
12	81143556	35521341	12	AI851047	null
12	81402630	35521341	7	AI849214	Transforming growth factor, beta 3

Chromosome 13

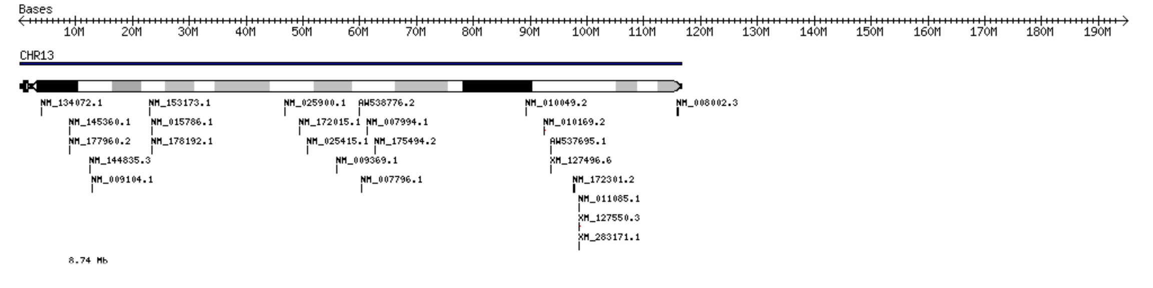

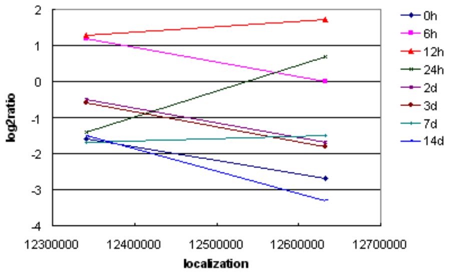

chr	start	end	cluster	RefSeq/AccNr	Name
13	12341658	12384692	11	NM_144835	Hypothetical protein MGC30806
13	12632282	12632169	8	NM_009104	Ribonucleoside-diphosphate reductase M2 chain
13	8741110	8746719	4	NM_177960	Isopentenyl-diphosphate delta isomerase
13	8741110	8746719	8	NM_145360	Similar to hypothetical protein FLJ20424

Chromosome 14

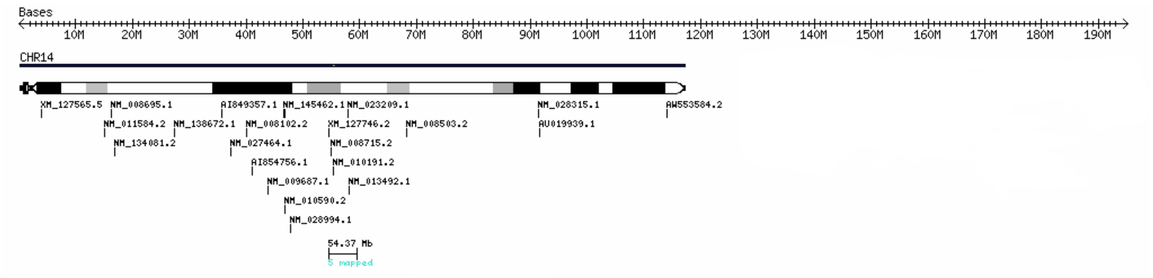

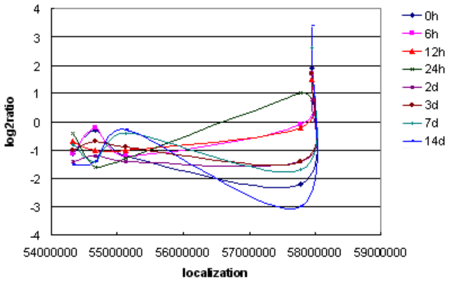

chr	start	end	cluster	RefSeq/AccNr	Name
14	54326868	54367927	8	XM_127746	RIKEN cDNA 2610207P08
14	54667333	54751436	12	NM_008715	EGF repeat transmembrane protein
14	55134053	55166264	12	NM_010191	Farnesyl diphosphate farnesyl transferase 1; squalene synthase
14	57791551	57803462	8	NM_023209	MAPKK-like protein kinase; TOPK
14	57957371	57967188	6	NM_013492	Clusterin; Apolipoprotein J (ApoJ)

Chromosome 15

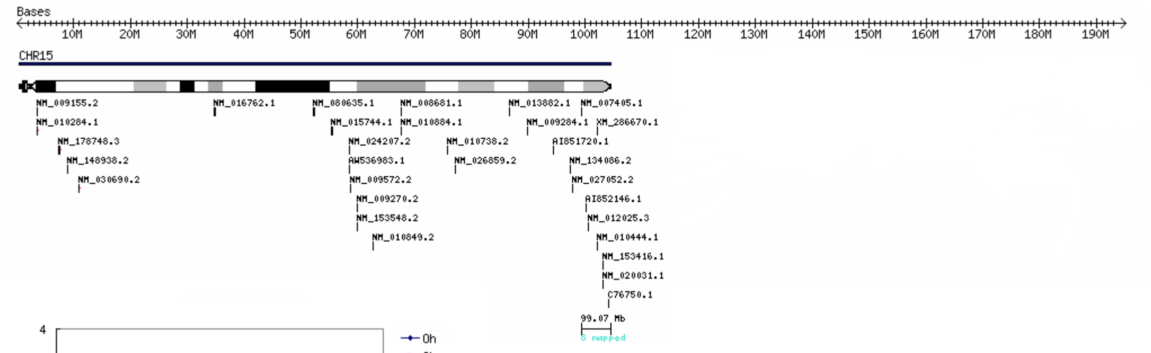

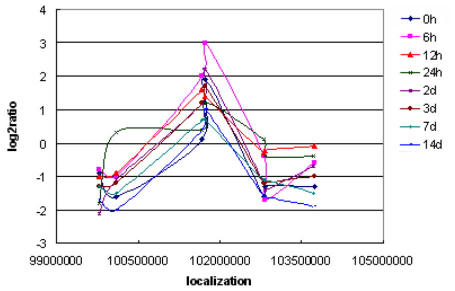

chr	start	end	cluster	RefSeq/AccNr	Name
15	99053155	99065790	9	NM_007405	Adenylate cyclase 6
15	99777728	102810121	12	AI852146	Formin-like protein
15	100082766	100112810	8	NM_012025	Rac GTPase-activating protein 1
15	101678883	101681014	9	XM_286670	Hypothetical protein
15	101734195	101742081	2	NM_010444	Nuclear receptor subfamily 4, group A, member 1 (NUR77)
15	102805078	102810121	8	NM_020031	Prefoldin 5; c-myc binding protein
15	102817154	102829206	8	NM_153416	Aladin (Adracalin)
15	103730071	101681014	8	C76750	Heterogeneous nuclear ribonucleoprotein A1

Chromosome 16

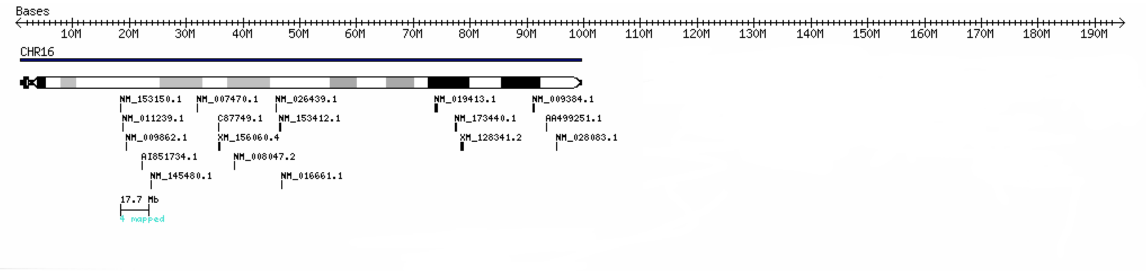

Chromosome 16

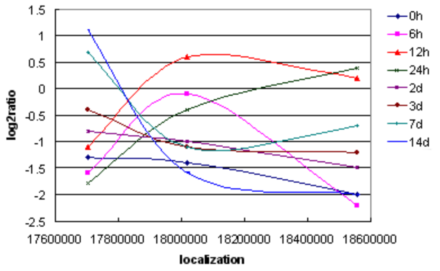

chr	start	end	cluster	RefSeq/AccNr	Name
16	17702844	17704902	3	NM_153150	Citrate transport protein; mitochondrial carrier
16	18018869	18024284	8	NM_011239	RAN binding protein 1
16	18557654	18588883	8	NM_009862	Cell division cycle 45-like protein
16	21482308	21482423	9	AI851734	Hypothetical protein

Chromosome 17

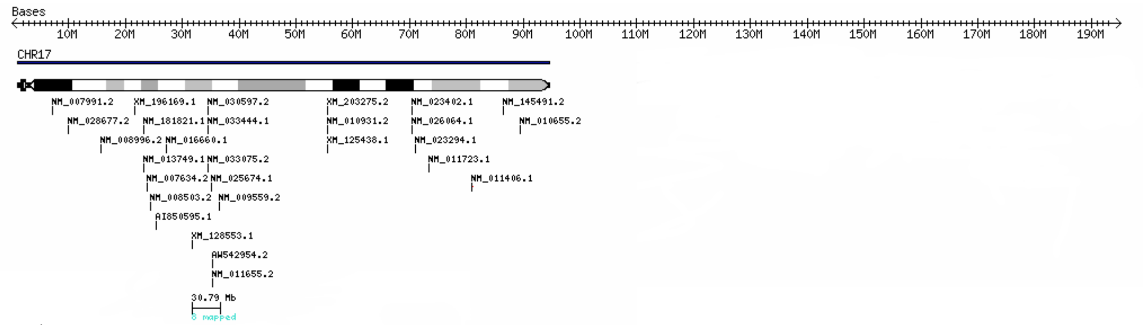

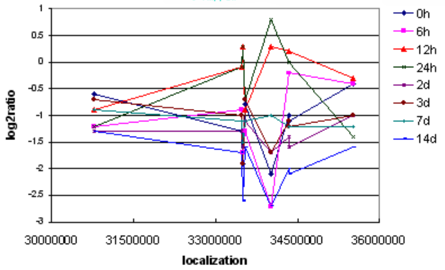

chr	start	end	cluster	RefSeq/AccNr	Name
17	30786556	30790469	12	XM_128553	RIKEN cDNA 2310063B19; epoxide hydrolase 2
17	33478506	33482258	8	NM_030597	SnRNP core protein SMX5
17	33494562	33494932	8	NM_033075	G7e protein
17	33548802	33555096	12	NM_033444	Chloride intracellular channel protein 1
17	34023132	34025825	5	NM_025674	Similar to transcription factor 19
17	34343782	34344160	8	AW542954	null
17	34345896	34347854	8	NM_011655	Tubulin beta-5
17	35527296	35533502	10	NM_009559	Zinc finger protein 57

Chromosome 18

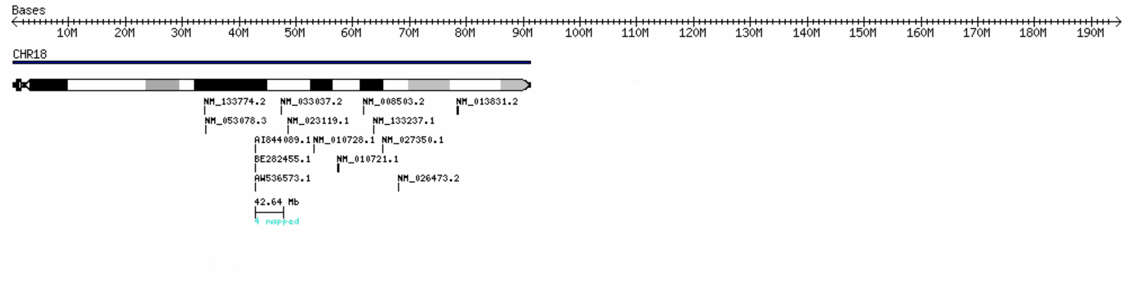

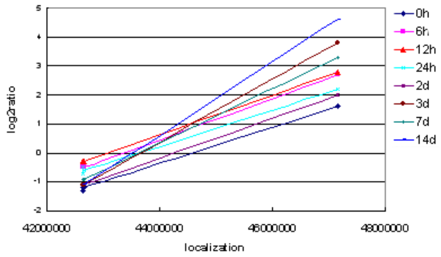

chr	start	end	cluster	RefSeq/AccNr	Name
18	42643277	42651084	8	AW536573	Leucyl-tRNA synthetase
18	42643281	42657783	8	AI844089	Leucyl-tRNA synthetase
18	42653546	42657783	8	BE282455	Leucyl-tRNA synthetase
18	47159021	47173316	7	NM_033037	Cysteine dioxygenase 1, cytosolic

Chromosome 19

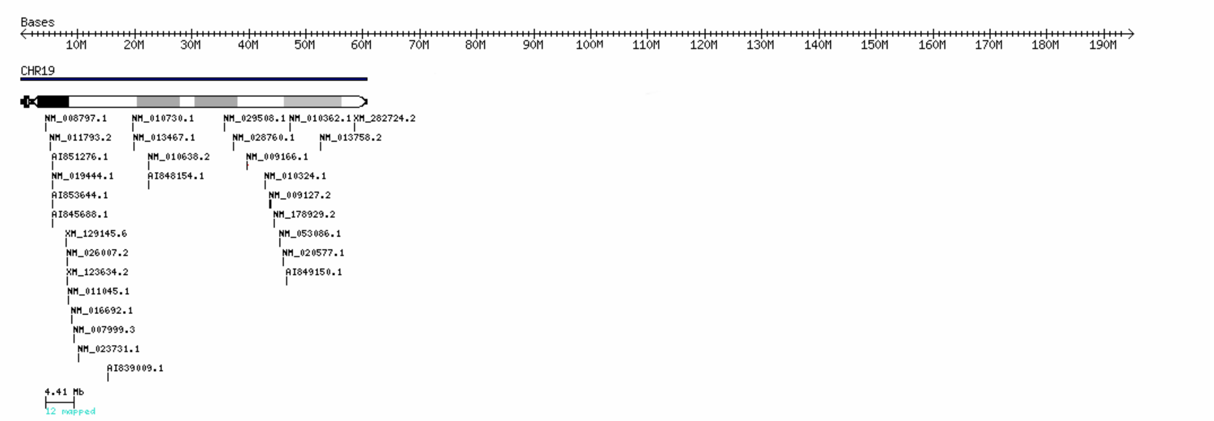

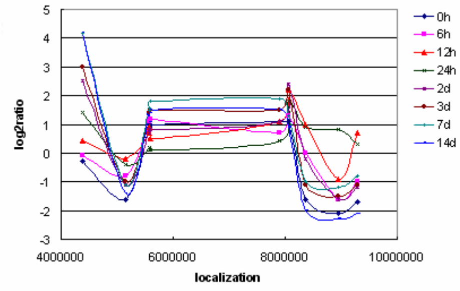

chr	start	end	cluster	RefSeq/AccNr	Name
19	4390068	4410167	6	NM_008797	Pyruvate carboxylase
19	5154179	5154836	8	NM_011793	Barrier to autointegration factor 1; breakpoint cluster region protein
19	5584597	7917517	9	AI853644	null
19	5586640	8383576	9	AI851276	null
19	5588772	5588291	9	AI845688	null
19	5591563	5591343	9	NM_019444	Receptor-activity modifying protein 2
19	7908278	7917517	9	XM_129145	TPR Domain containing protein
19	8067296	8077259	2	NM_026007	Elongation factor 1-gamma
19	8382319	8383576	8	NM_011045	Proliferating cell nuclear antigen (PCNA)
19	8971664	8994402	5	NM_016692	Inner centromere protein
19	9300180	9302769	8	NM_007999	Flap endonuclease-1

Chromosome X

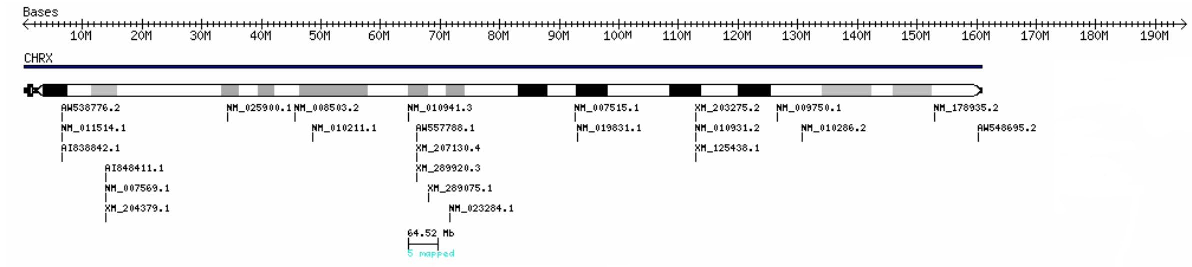

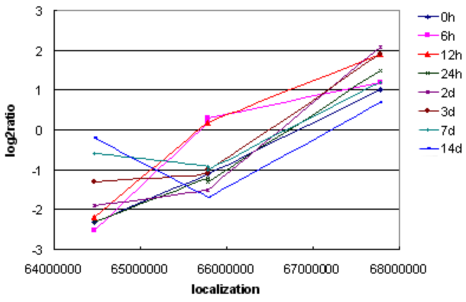

chr	start	end	cluster	RefSeq/AccNr	Name
X	64476905	64516912	4	NM_010941	NAD(P)-dependent steroid dehydrogenase
X	65781847	65783301	10	AW557788	Similar to filamin
X	65782363	65808113	10	XM_207130	Endothelial actin-binding protein
X	65782363	65808113	10	XM_289920	Endothelial actin-binding protein
X	67784147	67817633	2	XM_289075	Hypothetical protein
